# Supplementary material for: Dissecting Toxicity: The Venom Gland Transcriptome and the Venom Proteome of the Highly Venomous Scorpion Centruroides limpidus (Karsch, 1879)
Source: Toxins (Basel). 2019 Apr 30;11(5):247. doi: 10.3390/toxins11050247 (PMC6563264; doi:10.3390/toxins11050247)
Supplement: Supplementary file 1 [file toxins-11-00247-s001.zip › toxins-494793-supplemenraty materials/toxins-494793-supple figures-final.docx]

Supplementary Materials: Dissecting Toxicity: The Venom Gland Transcriptome and the Venom Proteome of the Highly Venomous Scorpion *Centruroides limpidus* (Karsch, 1879)

Jimena I. Cid-Uribe, Erika P. Meneses, Cesar V.F. Batista, Ernesto Ortiz and
Lourival D. Possani


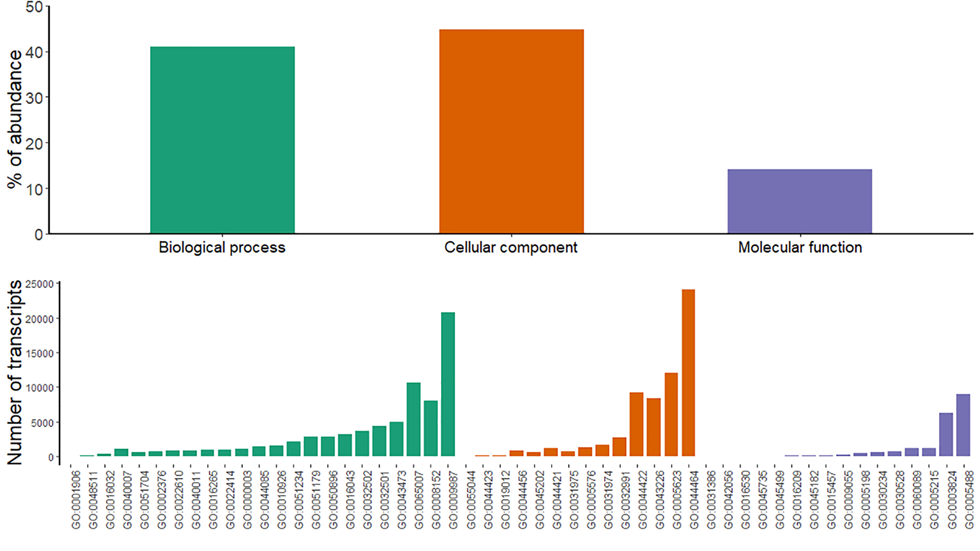


**Figure 1.** Distribution of the annotated transcripts in accordance to Gene Ontology (GO) terms.


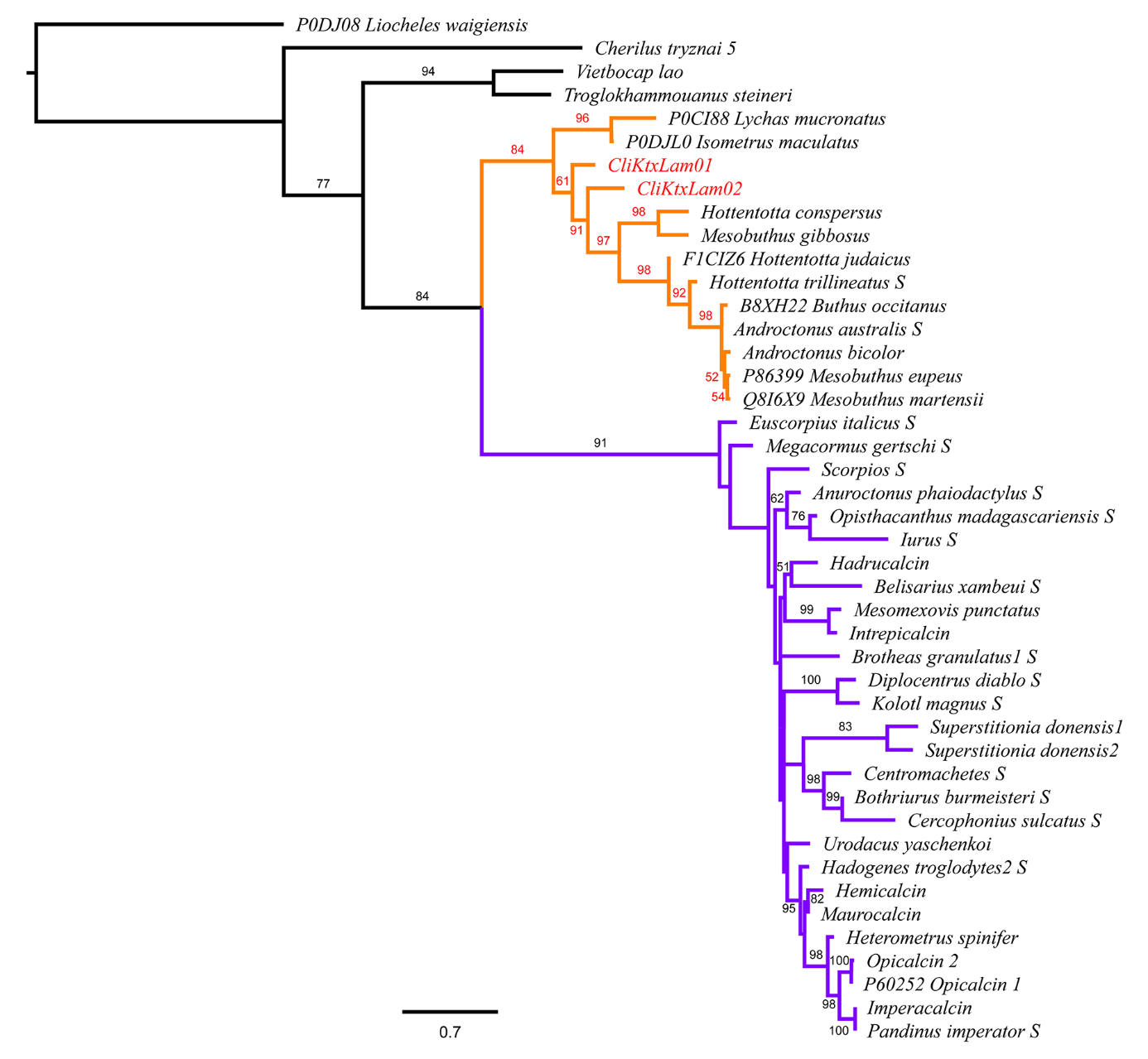


**Figure S2:** Phylogenetic tree of scorpion toxins with the ICK fold
