# Supplementary material for: Dissecting Toxicity: The Venom Gland Transcriptome and the Venom Proteome of the Highly Venomous Scorpion Centruroides limpidus (Karsch, 1879)
Source: Toxins (Basel). 2019 Apr 30;11(5):247. doi: 10.3390/toxins11050247 (PMC6563264; doi:10.3390/toxins11050247)
Supplement: Supplementary file 1 [file toxins-11-00247-s001.zip › toxins-494793-supplemenraty materials/toxins-494793-Supple Table S1.docx]

| **Supplementary Table S1**. Translated sequences of the 192 venom-related transcripts obtained from the transcriptomic analysis of the *C. limpidus* venom gland. Sequences were classified in accordance to Pfam domains present in venomous animals. ID reference protein: UniProt or GenBank accession numbers of the closest match by sequence. The predicted signal peptides are underlined, the predicted mature peptides are indicated in bold typeface, and the predicted propeptides are in italics. With regular font are indicated the sequences for which the predictors could not recognize the former features. | | | | |
| --- | --- | --- | --- | --- |
| TOXINS AFFECTING ION CHANNELS | | | | |
| Toxins active on Na^+^ channels | | | | |
| ID | ID reference protein | E-Value | Sub-classification | Amino acid sequence |
| CliNaTAlp01 | XP_023235403 | 2.00E-22 | α-NaTx | KSFILFICCLMVIDVVIES**KDGYPLDEEDCIINCDIGSCEKLCTSRKANTGHCNSLDLSCYCESLPLDVKVFDSNSKTCKY***KK** |
| CliNaTAlp02 | XP_023235403 | 2.00E-29 | α-NaTx | MKRFILFISCLMVIGVVVES**KDGYPIDSNGCTFRCDILINDCDTVCESRKANNGTCSSSNLACYCEGLPSDADVSDDPTKNDCS*** |
| CliNaTAlp03 | XP_023210703 | 2.00E-36 | α-NaTx | MKVLILIIASVLLIGVEC**KDGYPVDNKGCKISCVISGKFCDTECKMRKASSGYCYSLSCYCEGLPENAKVSGKATSTCG***GK** |
| CliNaTAlp04 | XP_023230636 | 1.00E-60 | α-NaTx | MYLRRISDMNSIFVVALTILLLGIERSES**RDGYPIASNGCKFGCSGLGENNPTCNHVCEKKAGSDYGYCYWWTCYCQHVAEGTVLWGDSGTGPCMS*** |
| CliNaTAlp05 | XP_023242001 | 9.00E-19 | α-NaTx | VLAIALIFVLLGIEGTQA**RNGYLVSKHNCRYGCSSLGKSEKCNELCKEKAGAFYGYCYAFVCYCEGMREDAIQYNRDQTRRDCQYDDDK*** |
| CliNaTAlp06 | XP_023242001 | 2.00E-42 | α-NaTx | VLAIALIFLLLGMEEVQA**KDGYPVNEHRCKIGCSSLGESEYCKKICKKKADTSYGYCYMWACYCESVGKNAVLWNDPTLGPCIPDGKRSLSS*** |
| CliNaTAlp07 | XP_023242920 | 1.00E-22 | α-NaTx | MKYSISIAVFCLLALEAESR**KDGYPVNSGNCKYECWNDDKCVDLCKKRGATKGYCYRRNVSCYCQGLPDNEPIMKSGRCNPGG***GRRK** |
| CliNaTAlp08 | XP_023242920 | 4.00E-32 | α-NaTx | MKFLLLIALTCLLALGVEC**KKDGYIVDSGNCKYECLRDDYCKDMCLKRKADDGYCFLGKFSCYCYGLPDNSPIKTSGKCK***GK** |
| CliNaTAlp09 | XP_023242920 | 4.00E-37 | α-NaTx | MKFLLLIALTCLLALGVEC**KKEGYPVDSGNCMYECLRDKYCNDLCLERKADSGYCYLGKFSCYCYGLPDNSPTKTSDRCNPAK***GK** |
| CliNaTAlp10 | XP_023228500 | 1.00E-48 | α-NaTx | MNCLILVAVSCILVLGVDGE**KDGYPDKGGNCKYECLWDTYCDELCKKKKAKSGYCYWGNVSCYCYGLPDNEPTRGSSGKCRPAL***GRK** |
| CliNaTAlp11 | XP_023238168 | 8.00E-20 | α-NaTx | MNYFILSLVAALLILHVNC**IKNDYPVDENGCKIACQENAPCAAACKFNKAEGGVCPWSYRCWCYGLPDSVTTEENCK***GKRK** |
| CliNaTAlp12 | XP_023238168 | 2.00E-40 | α-NaTx | MNYFILLFVATFLLLDVNC**KKDGYPVDAKNCKFECWKNAYCDKLCKDKRGEGGYCYRLKLSCWCTGLPDKVAIKTNQACKRK***GK** |
| CliNaTAlp13 | XP_023228501 | 6.00E-47 | α-NaTx | MNYFILLFVATFLLLDVNC**KKDGYPVDAKNCKFECWKNAYCDKLCKDKRAESGYCYGWNLSCYCEGLPDDEPIKTSDRCYGT***RR** |
| CliNaTAlp14 | XP_023216724 | 2.00E-53 | α-NaTx | MKLLLLLLIALLIEVNGL**KNGYVLHKNSNCKYSCNITDKWGYCSPLCQKKHGKTGYCYFFACWCEGLPSDTPVYGDEGYTCW*** |
| CliNaTAlp15 | F8UWP3 | 2.00E-12 | α-NaTx | DVNC**KKDGYPVTSDKYSLPCWHSKVCNRICILLKAEKGYCPYSLYCWCYGLPDDAPTSG*** |
| CliNaTAlp16 | ACD11820 | 4.00E-18 | α-NaTx | MVSFILVVTSFLVLDVQG**KKDGYLYDMYDCRYKCWRNAYCDKLCKENKAEGGYCYALNLWCYCTGFFDDADAVSYGECMND***RK** |
| CliNaTBet01 | XP_023241641 | 9.00E-52 | β-NaTx | MKFLILIVASLVIVGVQS**KDGYPMDHKGCKISCVINNQYCETECVKVLKGKKGYCYFWKLACYCEGLPNWAKVWDRATNKCRA*** |
| CliNaTBet02 | XP_023241641 | 1.00E-44 | β-NaTx | MKFLILILASLMITGVQS**KDGYPIRDDGCKIPCVINNRFCEIECVNALKGKKGYCYFWKLACYCEGLPNWAKVWDRATNKCTA*** |
| CliNaTBet03 | XP_023241641 | 8.00E-32 | β-NaTx | MKVFILIIASLMIMGVLS**KDGYPMDRKGCKLFCVINGKRCDTECRIMLKGKTGYCFTPKLACYCEGLHKLVRVSEYPTNKCKA*** |
| CliNaTBet04 | XP_023235256 | 7.00E-20 | β-NaTx | VASLMIIGVQS**KDAYGKRKDGSTIPCLLDSSYCNRECVELKAKSGNCYFLAMTCYCQGLPD*** |
| CliNaTBet05 | XP_023235256 | 2.00E-36 | β-NaTx | MKLLILILASLMRLGVQN**KDGYAMRSDGCTIPCLFDNSFCNRKCIEQKGKSGYCYFLKQSCYCAQLPDDKVYPSATNKCRA*** |
| CliNaTBet06 | XP_023235257 | 4.00E-45 | β-NaTx | MKFLILILASLMITGVQS**KDGYPIRDDGCKIPCVINNRFCEIECVNALKGKKGYCYFWKLACYCEGLPDWAKVWERATNKCRA*** |
| CliNaTBet07 | XP_023235252 | 2.00E-47 | β-NaTx | MKFLILIVASLMIIGVQS**KDGYPMNHEGCKESCLINNRYCEIICVDFMKAKKGYCYFWKLACYCEGLPDWAKVWERATNKCRA*** |
| CliNaTBet08 | XP_023242923 | 1.00E-40 | β-NaTx | MNYFILILIAALLIMDVNC**KKDGYPVDAKQCRYNCWKNTYCDKLCKEKKGESGYCWWNLSCWCIGLPDNTNTKMNPFCQGLD***GK** |
| CliNaTBet09 | XP_023237823 | 2.00E-51 | β-NaTx | MNYFVLIFVAALLMLGVNC**KKDAYPVDGSNCRYPCWRNAYCDELCKERKAESGYCYTWSLWCWCIGLADDTPTQVNAGCAPS***GK** |
| CliNaTBet10 | XP_023237823 | 8.00E-18 | β-NaTx | MNYFLLTLVAALLILHVNC**IKNDYPVRNGCRIKCQENASCDEACKFNKAEGGVCPWSYRCWCYGLPDNIDTEKNCNG*** |
| CliNaTBet11 | XP_023237823 | 8.00E-18 | β-NaTx | MNYFILSLVAALLILQVNC**IINDYIVYQNGCRSVCTANKYCDEFCKANKAEGGVCPWSYYCWCYGLPDNIDTEKNCNG*** |
| CliNaTBet12 | XP_023229318 | 7.00E-45 | β-NaTx | **KGYLVNEDTGCKYVCLLSGPSRYCESECKAKKHGGSCGYCRRFACWCAGLPKSTPTWPLPNKSCSIKHPLEDRED***KKIQKRDFSLYQQIYCENY** |
| CliNaTBet13 | XP_023223307 | 3.00E-51 | β-NaTx | MKSFILFICCLMVIDVVVES**KDGYPMDNKGCKVWCVINSKSCDNTCKAMKGKSGYCYTLGLACWCEGLPSDAKVWDRATNKC***GKK** |
| CliNaTBet14 | XP_023209741 | 2.00E-49 | β-NaTx | MNVALLFLMTLSLVLIHNGES**KKKIPGAYPINENKCTYGCFHADKDKIRCSNFCKKLGGSSGYCYWMTCYCEYLPAEVPQINKIELISCGASIIGVNDTVYL*** |
| CliNaTBet15 | XP_023209741 | 3.00E-09 | β-NaTx | VGA**VVNGFLVSKNRCLYTCKLDDSGHKKCRDRCGALGASWGYCELYSCYCLGLPDDVETVNSIADSPCTTGA*** |
| CliNaTBet16 | XP_023209741 | 6.00E-07 | β-NaTx | VGA**VVNGFLVSKNRCLYTCKLDDSGHKKCRDRCNALGASWGHCELYVCCCIGLPDDVETVNSLADSPCTTRA*** |
| CliNaTBet17 | XP_023243062 | 8.00E-12 | β-NaTx | MFKILILAAIFLMFVAIEVEA**DVNGFLVNKNGCVYPCKSDESGNKKCHKRCYILGGSRGYCKLYNCYCEGLPVDVKTVKSFTYSPCTTSGN*** |
| CliNaTBet18 | XP_023240702 | 4.00E-50 | β-NaTx | MKNLILIVFSVAAAIHEMEC**LLEYAYPIAKNGCRISCVPLEDDDLCIAYCQELGAEDAVCSAPASACLCYDAPNTMMTWNAASSKCPYWNENSVNFLV*** |
| CliNaTBet19 | XP_023211611 | 2.00E-32 | β-NaTx | MKVLILIIASVLLIGVEC**KDGYPMDSKGCKISCVINDKYCENECKMLKGSGYCYSRGLACWCEGLPEDAEVWDS** |
| CliNaTBet20 | XP_023211611 | 3.00E-34 | β-NaTx | MKVLILIIAFVLLIDVEC**KDGYPVDNKGCKISCVINDTFCDNECKARKGKSGSCYSLKLACWCEGLPEDAEVWDS** |
| CliNaTBet21 | XP_023211611 | 1.00E-37 | β-NaTx | MKVLILIIASVLLIGVEC**KEGYPMNSKGCKIGCVIGNTFCDTECKMLKASSGSCWTFGLACWCKGLPEDVEVWDSATNKCG***GK* |
| CliNaTBet22 | XP_023209523 | 8.00E-69 | β-NaTx | MLNTLISLFLVVAVSLLTYVNVEG**TEVPGGYPVNRFNCTYPCYYGEDEEQCQQFCTLLKGGFGYCYMYTCYCERLPESVKQIKKFKISGCSNGSWDITSVTKLYGSSR*** |
| CliNaTBet23 | XP_023209523 | 8.00E-36 | β-NaTx | MEIVYKILMLGALLLMCVKVEG**SDVPSAYPVNRWNCTYPCYYGEDNDKCRKFCLILGGQHGYCYWYTCYCENLPESVKHIKHLGVFGCSNGSWVVSSTTAKP*** |
| CliNaTBet24 | XP_023209523 | 2.00E-12 | β-NaTx | MFKSLLVVAAFLLTLINA**LEDGYPVNENGCTYPCYHTASNEKLCQERCGSLGAVFGYCRMMICKCELLPEHVPHVTSM*** |
| CliNaTBet25 | XP_023240701 | 1.00E-60 | β-NaTx | MKNLILIVFSVAAAIHEMKC**SRTGYPIRENGCRISCVAGIEDNLCDTYCKNNGAEKGVCKEPASSCYCEKGPFHMATWNVDYVNCEAWKENSVRFYVDAN*** |
| CliNaTBet26 | XP_023209923 | 3.00E-27 | β-NaTx | MNSLLMIIACLVLIGIVKA**KDGYPVDISTGCKFRCTNKTGERNCNMLCKHRNRGSRGYCYNRECWCEGMPESTPTWPIP***GKKCK** |
| CliNaTBet27 | XP_023213214 | 3.00E-47 | β-NaTx | MKIVVLFFCLVGTVVA**VRRHGYILMDGKPMECPMEEERFLEQGTDYCDLKCKERGAKTGVCCYGGCYCYDIEDDEKVVDVSRFRSKLCKYLTEV*** |
| CliNaTBet28 | Q95WD3 | 6.00E-14 | β-NaTx | MKTATIVILTVLLIGVQA**KDGYLINEDYCRYRCWNLGKNTYCNNLCLTNGGKFGYCYRFSCYCEKLPDTYTIWGEPGTPTCGHASD*** |
| CliNaTBet29 | P01491 | 3.00E-42 | β-NaTx | LIITACLALIGTVWA**KKDGYLVDKTGCKYTCWKLGENKYCNRECTWKHRGGNYGYCYGFGCYCEGLADSTQTWPLPNKRC***GKK** |
| CliNaTBet30 | AAL23432 | 1.00E-23 | β-NaTx | MKTFVLALCLVLIGMVYA**KDGYLVSRHNGCKLSCAPMIGDRYCHIECTSMNHRGDEGYCYLLGCYCRGMPENAEVYPLPNKSC***GK** |
| CliNaTBet31 | P59899 | 4.00E-53 | β-NaTx | LIITACLALIGTVWA**KEGYLVNHSTGCKYECYKLGDNDYCLRECKQQYGKGAGGYCYAFGCWCTHLYEQAVVWPLPKKTCN***GK** |
| CliNaTBet32 | P59899 | 3.00E-39 | β-NaTx | A**KEGYIVNLSTGCKYECFKLGDNDYCLRECKQQYGKGAGGYCYAFGCWCTHLYEQAVVWPLPNKRCK***GK** |
| CliNaTBet33 | Q7YT61 | 3.00E-55 | β-NaTx | MNSLLIITACLVLFGTVWA**KEGYLVNKSTGCKYGCFWLGKNENCDKECKAKNQGGSYGYCYSFACWCEGLPDSTPTYPLPNKSCS***KK** |
| CliNaTBet34 | Q7Z1K5 | 2.00E-49 | β-NaTx | MNSLLMIIGCLVLIETVWT**KEGYLVNMKTGCKYGCYKSNDNDYCDRKCKAESGNYGYCYAVGCWCEGLPNSKPTWPLPGKSCS***GK** |
| CliNaTBet35 | P59865 | 2.00E-34 | β-NaTx | TACLVLIGTVWA**KKDGYLVDKTGCKYTCWKLGENKYCNSECKEVGAGYGYCYAFGCYCEGLPESVPTWPLSDKTCGTK*** |
| CliNaTBet36 | Q8WRY4 | 7.00E-53 | β-NaTx | MNSLLMIIGCLVLIGTVWA**EDGYLFDKRKRCTLACIDKTGDKNCDRNCKKEGGSFGHCSYSACWCKGLPGSTPISRTPGKTC***KK** |
| CliNaTBet37 | C0HK69 | 5.00E-39 | β-NaTx | **EGYLVDYHTGCKYTCAKLGDNDYCVRECRLRYYQSAHGYCYAFACWCTHLYEQAVVWPLPNKRCK***GK** |
| CliNaTBet38 | P58296 | 8.00E-34 | β-NaTx | MKIFIAFAFISALLLLGVES**ARDGYPVDEKGCKLSCVINDKWCNSACHYRGAKYGYCYSGKLACYCEAVPDNVKVWTYET** |
| CliNaTBet39 | P58296 | 1.00E-36 | β-NaTx | MKIFIAFAFISALLLLGVES**ARDGYPVDEKGCKLSCVINNKWCNSACHSRGAKYGYCYTGGLACYCESVPDDVKVWTYETNTC***GKK** |
| CliNaTBet40 | P58296 | 2.00E-27 | β-NaTx | MKIFIAFAFISALLLLGVES**ARDGYPVDEKGCKLSCFMNHEPCRKACVSRGGKYGYCYVAGRECYCEGVPDNVKLWTSETNTC***GKK** |
| CliNaTBet41 | ACD11838 | 4.00E-07 | β-NaTx | MHYPGLYLLILFIILPPAFL*SEFEERR***SGFLLDRNHCKIECSLLGTNNMCETRCTSLGATNGYCKYFACFCTNLRNEIKIWGDDVQCKW*** |
| CliNaTBet42 | P0C1B5 | 2.00E-08 | β-NaTx | SPVEGNSYKHNASRRSAEYIDKTGTEITY**GEAKGAYPISVDCTYYKCLNLGVNDHCKDVCKHHFGGYGYCYAFKCYCEELNPKYAHYYMRFMQRDR** |
| CliNaTBet43 | D9U299 | 2.00E-12 | β-NaTx | LTVFLIGRR**TVLGQGQYPRYVDNSYYKCGNLGENDYCVSVCKQHTVRYGYCYLFKCYCEGLPDTHIYHADLKRIRGNQVDQSLTKKGIKA*** |
| Toxins active on K^+^ channels | | | | |
| ID | ID reference protein | E-value | Sub-classification | Amino acid sequence |
| CliKTxAlp01 | AIL48749 | 1.00E-04 | α-KTx | MKDFYVILIILSICPMFDFA**VKFTPIDKTCVIKFECWVACWNEKGKPFGICSDRKCKCLV*** |
| CliKTxAlp02 | AIL48797 | 5.00E-27 | α-KTx | MKAVTILLLLAFILCAQEIANVEA**ERCVFWKCNSDCIRRRYRGGICSGILNNTYCTCLR*** |
| CliKTxAlp03 | O46028 | 3.00E-15 | α-KTx | IHSPFFHLFVILKSSENTFANWNVE**AVACVHRTCDSNCKEMGYKSGKCINRTCKCYP***RRK** |
| CliKTxAlp04 | O46028 | 2.00E-24 | α-KTx | HTFANWNAEA**AVCVYRTCDKDCKRRGYRSGKCINNACKCYPY***GK** |
| CliKTxAlp05 | O46028 | 1.00E-26 | α-KTx | MAGIAKITLILLFLFVTMDTFA**DWNAEANVACVHRTCDSNCKRNGYKSGKCINRKCNCYPH***GK** |
| CliKTxAlp06 | P0C182 | 1.00E-15 | α-KTx | MKAFYGILIILLFCFMFDLNES**IFIDVDCTVSKECWAPCKAAVGVDRGKCMGKKCKCYP*** |
| CliKTxAlp07 | Q9TXD1 | 4.00E-17 | α-KTx | MKVFYGILLIFVFCSLYNLSQS**TIINEKCFATSQCFTPCKKAIGSLQSKCMNGKCKCYNIG*** |
| CliKTxAlp08 | Q9TXD1 | 4.00E-17 | α-KTx | MKVFYDNLSQS**TIINEKCFATSQCFTPCKKAIGSLQSKCMNGKCKCYNIG*** |
| CliKTxAlp09 | P0C165 | 1.00E-19 | α-KTx | MKAFYGILIILLFCSMFNLNES**TVINVKCTSPKQCLPPCKEIYGRHAGAKCMNGKCHCSKIG*** |
| CliKTxAlp10 | P45629 | 2.00E-18 | α-KTx | MKAFYGILIILLFCSMFNLNES**TVINVKCTSPQQCLRPCKDRFGQHAGGKCINGKCKCYP*** |
| CliKTxAlp11 | XP_023238900 | 7.00E-36 | α-KTx | MNKVYLVAFLVLSVLLVANVSPVEG**IPTGGCPVSDALCTKYCKSNKYGKSGKCTGPNKTTCKCSL*** |
| CliKTxAlp12 | Q95NJ8 | 6.00E-13 | α-KTx | MKFVFALLLLSILTITTVPTLVRG**SGQCQRDSDCQRECMTVDKCQYGTCYCKTT***GK** |
| CliKTxAlp13 | P56219 | 5.00E-16 | α-KTx | MKGFYGIFIILLFCSMFILSQQ**VFIKHKCTKSPQCKKACKKAVGKAAGKCVKGKCKCNARCIII*** |
| CliKTxAlp14 | Q5G8B6 | 2.00E-20 | α-KTx | MKAVYGILIILLFCSMFDLSQQ**TFINHKCKSSSECLPACKAAIGRASGKCINSTCKCYY*** |
| CliKTxAlp15 | Q5G8B6 | 3.00E-19 | α-KTx | MKAFYGILIIVLFCSMFNLSEE**VFIDKKCSSSSECWPACKKAVGTFQGKCMNGGCKCYP*** |
| CliKTxAlp16 | Q5G8B6 | 1.00E-16 | α-KTx | MKVFYGILIILLFCSMFNLSQQ**TFIDVKCGSSKECWPPCKAAFGKARGRCMNKQCNCS*** |
| CliKTxBet01 | XP_023220228 | 3.00E-46 | β-KTx | MVAAGRRWIWALLASLLLLHSLAEA**GRGKEIMNKIKKKLADAKVTVKGAWDKLTSKSEYACPVIEKFCEDHCAAKETVGKCEDFKCLCLKPE*** |
| CliKTxBet02 | XP_023220230 | 1.00E-54 | β-KTx | MERKLAVLLLLLGMVALASC**GLREKHVQKLLSLVVPEGQLRKILQMVVHKAAKSQFGCPLYEGYCETHCQDITNKDGDCHGMKCKCE*** |
| CliKTxGam01 | Q86QT3 | 2.00E-34 | γ-KTx | MKVLILILIIVSVMIMGVEM**DRDSCVDKSRCSKYGYYQECQDCCKKAGHNGGTCMFFKCKCA*** |
| CliKTxGam02 | Q86QT3 | 2.00E-25 | γ-KTx | MKVLILILIIVSVMIMGVEM**DRDSCVDKSKCSKYGYYGQCDECCKKAGDRAGNCVYFKCKCNP*** |
| CliKTxGam03 | XP_023241648 | 9.00E-35 | γ-KTx | MKNILLMILILSAFILNEVEL**AKDSCTHLKRCGKYGFYKNCSECCREFGHEGGYCTMFKCKCKT*** |
| CliKTxDel01 | P0DJ50 | 3.00E-19 | δ-KTx | MKFVVTSLVVILFFHSFCTS**DDDVDCSLPPDSGLCLALFPRYYYNAKSGKCESFTYGGCGGNSNNFENKNECCKACGDDRC*** |
| CliKTxDel02 | XP_023217495 | 5.00E-44 | δ-KTx | MNIKFASTIILILCMYSDA**QEVDCNLPAETGPCKAAFRQYYHNSGSGKCEIFIYGGCRGNSNRFSSLDECCSHCFAKNC*** |
| CliKTxDel03 | XP_015905918 | 2.00E-18 | δ-KTx | MKLMLVSVALLLVQSFVKS**EDVDCSLPPRNGMCLAYFRRYYYDPKFDKCKVFIFGGCNGNSNNFGSMEECCKACGKGQCKTG*** |
| CliKTxLam01 | ADY39527 | 3.00E-14 | λ-KTx | MNLRFIIILGVLLASGVCSFARIDDEQAR**CNRLGKSCDSDSDCCRYGERCLSSGRKYVCKMDQGP*** |
| CliKTxLam02 | AIL48798 | 1.00E-10 | λ-KTx | MNLRFIIILGVLLANGVCSFVDEQAR**CNNLGKPCDSDNDCCTYGERCLSSNGKDYFCSVDPGP*** |
| Host Defense Peptides | | | | |
| ID | ID reference protein | E-value | Sub-classification | Amino acid sequence |
| CliHDPDef01 | Q6GU94 | 7.00E-32 | Defensin | MKAIVVLLILALILCLYAMTTVEG**ACQFWSCNSSCISRGYRQGYCWGIQYKYCQCQ*** |
| CliHDPDef02 | AIX87626 | 1.00E-22 | Defensin | MKIVAVLFLLAFVFCTLEIATVEAG**FGCPFNQGACHKHCQSIRKRGGYCDGFLKHRCRCY*** |
| CliHDPDef03 | AIX87626 | 3.00E-23 | Defensin | MKAIVLFFFLAFTFCTLEIMPVEAG**FGCPLNRYECHNHCRSIGRRGGYCKGTFRTTCTCYNSK*** |
| CliHDPDef04 | AIX87626 | 1.00E-14 | Defensin | MKVIVVLLLLAYVFC**TLEVMSLETEFGCPSIEIRCYIHCRSVNRKRGRCTGTSKQICTCYD*** |
| CliHDPDef05 | AIX87626 | 1.00E-07 | Defensin | MKSIIIIFLLAFVFSTQA*LEGYVKTDR***GCPFLSIECDGYCVNQGYKRGYCDGPFNTKCTCFNK*** |
| CliHDPDef06 | AIX87626 | 6.00E-12 | Defensin | MKAIVLFFLLAFVFCTLEVPVDA**DRGCPSDRRCNAYCRKIGSRGGFCAGPRKLRCACLR*** |
| CliHDPND201 | AMX81493 | 7.00E-29 | NDBP2 | MKGKTLLVVLLVALLIAEEVNG**FKFGGFLKKMWKSKLAKKLRAKGREMIKDYANRVLEGPQEEAPPAE***RRR****** |
| CliHDPND401 | SBQ16531 | 1.00E-35 | NDBP4 | MQFKKQLLVIFFAYFLVINESEA**FLGSLFSLGSKLLPSVFKLFQ***RKKSRSINKRDLEDLYDPYQRNLEMERFLKQLPMY** |
| CliHDPND402 | S6D3A7 | 5.00E-35 | NDBP4 | MQIKHLIVVFFVVLIVADHCHA**FFGLIPSLVGGLISAFK***GRRKRDLTAQLNQYRHLQKREAEFREFLDNLPIY** |
| CliHDPAni01 | XP_023227050 | 4.00E-40 | Anionic peptide | MVRKSLIVLLLVSVLVSTFLTTDA**YPASFDDDFDALDDMNDLDLDSLLDLEPADLVLLDMWANMLENSDFEDDFE*** |
| Enzymes | | | | |
| Proteases | | | | |
| ID | ID reference protein | E-value | Sub-classification | Amino acid sequence |
| CliEnzMtp01 | XP_023221573 | 0 | Metalloprotease | VNIIVNRIILLKEKQPNLLISHHAGQTLSSFCKWQESIKRNENAILSYSHNHHDNAILITRYDICFDKDEPCDTLGLAQVSGMCERGRSCSINQDIGLTSGFTIAHELGHNFGMQHDGSGNDCLVKINEPGYIMSTQLSRLVYPVKWSRCSRQYITDFFDSGQGDCLQNVPPMNDYAYTDLLPGQRYNADQQCRSQFGPHSSVCQDIQREVCGTLWCSKHKGRCVSNNTPLLDGTVCNSTTVPNGWCYGGECVDYGTIPSKVDGGWGQWSEWGVCSRKCGGGIETSERQCDKPRPSHGGRFCVGKRKRYRSCNVKECPSNSEDFRAMQCRKFNNIKFRGKYYQWIPFTADQVNSCSLYCQAVGYNFYVEQAVKVIDGTRCNQDSLDVCINGRCMPVGCDGILNSKVT |
| CliEnzMtp02 | XP_023210991 | 0 | Metalloprotease | MPYFFLGLAPVSGMC**NPLNSCTISEGTSFQTVLVVGHEFGHSMGMEHDGRQDDNHCDPEKYIMSHTLGPGKTSWSTCSREYLEKFLRSSQSRCLQYTNSGVNMLKKLSPVLLPGQEYDANKQCQLRYGEGSRRSTIQSSEDICYMLRCDVGFGGKDVSFTAHPALEGTVCGRNKMCQAGKCTTANWRRNRAGPVDGGWSAWSNFGPCSSDCSTSRNTDAVGVMISTRRCNNPRPVNGGRYCQGADRRVRICDANRICSAAANAVTLSEYISETCQQASRLDTTLDPSGTQFPSHDNSHSCYVWCHKKQGGYITHGWKLPDGTPCWRGYHPQNQFCFQGKCNTFDCNGYTIETSNETPCPNRPTLATRHITEWSPWKKISECKHSCIVAGKGFTLVSRKCNTVTGCTGVRDSFQLCDTNSKCTVMKSAEVYATDLCKKYRNRYPTVLSGKGRQLAPRSGNPHAACTVACQDQVWQGTHYQMDAFEDGKFPSGTDCSGGNYRKKAYCLNGKCVEFDENNVPKVEDNNSILQVKKLFKYYRHRR***DTSEQENSVENLFVNNKYTIDQNKRKKSIKYKLLTQNFHKYTYRWAIEMSECSEPCGGGIRNISMFCVRENVPVESAYCESIIKPMESGIQECNMHPCAGKWQIRHWTPCSVTCGNGTRTRYAICVQQLTEDLYSFISHINCPKTEEIVEKCEMPKCS** |
| CliEnzMtp03 | XP_023217439 | 0 | Metalloprotease | MSIDGRRRTSFRMDRTTATLLLAACLCAAVRG*HRTPPGTSDRPEVVVPERIDGAGRSVGHGLARHFGRGKRSAEEAVHYRLPAGGKDLLLTLRPNRGLLAPAFVVERRRGPNSTFHR***SRKPSEDGLCYYRGNVRNDTGSSVALSTCDGLMGVIRTKGEELLIQPMPGGSEKSGRRPHLLYKRSTTEVHGREAKCGNRDDVARALKYRAEWEKKKKRKKKGRSRSKRSVSVERNVETLVVADKAMVDYYSDEDIETYILTVMNVVSSLYHDASIGNAINVILVRLILLESEEQEKEDLYISHHADDTLKSFCKWQKYVNPRDENHPHHHDVAVLLTRYNICTRVNEPCSTLGLAEVAGMCQPHRSCNVNEDTGLALAYTIAHELGHNFGMSHDGPHNGCQAPLGERQHVMSPHLNSDASPFVWSNCSRSEITKFLDRDWGSCLDDEPSDHEFTFPELPPGAMYNSDHQCRLQYGPEAEYCEGIEDVCQTLWCRQDNKCVTRLEPAAEGTLCDKNKWCYMGKCTVIGERLETINGEWGSWSPWSECSRSCGAGVMHSERHCDNPMPGNGGQYCIGERKRYRICQTEPCAENSPSFRSVQCSEFDNIPYKEQLYTWLPVSTPLTPCQLHCKPKGKFFSVMLSDTAKDGTPCNPGTNDMCINGKCRNVACDWGIDSNAQEDRCGICHGDGTQCQTIHGNFLQKKGLGYVEVLKIPTGARNFRIEELGDANNYIAIQDSSREFQLNGQWFIQWSGEYPVAGTIFYYSREGEKESLHAPGPTKEPVYILLLFQTENPGLSYEYTIPNKNITRKPEFHWQYTDWSVCSATCNGGIQISRARCLEKEAGLVEDKYCSDLEKPVDISRICNRHQCPARWWAGPWQHCSVSCGSNGIRKRTVICVRSLGPDEQIALLDEDCYAEDRPSDSEPCRHKHPCLMHAHWETGQWTDVCHGDPCNYQSRHVYCNIPNGFCNEKEKPISRRKCGNITCGVWVIGNWSECSKTCGTGYRRRNITCQGGEACQQAIKPVKETTCNEQPCLPFDDTQIFGVVHKSNDHKNNKDGSDHKHRHHHGEDKHEENENEERRSDKVAKDVIVVPLYEVPDNRVEDVKVDNEIDEGIHQIRREPPTVPEGGRSNPKQDSQKYEWQVTMWEKCSKPCDGGVRNRRALCFDAVTKHMVVSELCDPFEMPSTEESCNLEPCLDWILTEWSECSSSCDRGLQYRKVYCPEEDKCDPEAKPSETRVCLIKSCLQWIAGPWSQCSASCGEGFRRRHVKCVDLSTQASSSRCSADDKPSHLQQCVNQECAEHNSAFSQCRDKLEVALCQSLRHMCDTWYFKAKCCHTCNKRINSRRRTYGSRKVGSS*** |
| CliEnzMtp04 | XP_023216256 | 0 | Metalloprotease | ILWEENLVTSKENNVTLGDTFYPVIYSIPKNETDISNNEHEKLVIIKTGKVVFYVELYPNYNLLSNNFEESDIFNHSNAPCLFQGPIVSHKGGMAAISICHGDGILHGTLMLTEDTYFIKPLSIVRTDMKLSANQKSPVPHILSKVLRTETDICGTNESGREIITRTKILNRNKRSVKKYTIETAVFIDEKLKHHYTEKSFKTNAVVLSILNQMQLVFKYGSLNFPIRITIVELNKLNYRKKISRKVDAGKYLKEFCKWQNDKKKESYTKWDLAILLTGFDIYRGFKKNIVGLAYSNQMCTESACVIAEGFQFDAGLILAHEIGHSLSMKHDGAGNSCDPEKYIMSSSIGGGKTKWSKCSNKYLRDFLRTKTSKCLRKTSTRRNYLKKENQFPGEKYPPKSQCHLSTGKDSFPFVENSSDDICSLLWCMNKKWRISSHPALDGSECGSNKICFEGNCIPRNNIKWYNYY* |
| CliEnzMtp05 | XP_023227092 | 0 | Metalloprotease | *NTNDTDEGNLTETARTRR***NIQELIKYIEVGIVIDKTMFNMKNGIHSAVIGVIIQSVNYVDLFYKKMNTRLSIVYVETWNDNDYVDTESPLQSIMNNFINYATDKLSTINRDVMLFVTGHEIQEEKLGICTPRSICTDQSAIILQGRTDTHIIAGTTAHLLGHTLGINHDSADCICEDWWRGCIMEETLTNSTRSHFSSCNLETYLNILQNNEAICLFNKPYPLENIPFCGNGIIETDEDCDCGSIQDCLDNEPCCNPITCKLKHGAECSLGECCYNCKLKPKGHLCRKSVSECDIPEYCDGVHGKCPTDIYQKNGKLCGDGENYCYLGTCPSPRSLCEHIWGYNSYPADDICFERLNVQGGLGGHCGRDGHGGYKKCLPENVKCGALQCQKGLYNKPLMEPDTEFTSAEFTNNGSISKY*** |
| CliEnzMtp06 | XP_023216275 | 2.00E-173 | Metalloprotease | MRASKLHLFFSIIQVIQIRNCFLLKQIKISDDIVYPLLYHTPRDKRDISDNTKEKLLIIRTSNTTFYIELIPNNELLSEDFEFEGFTYENHSSNPCLFHGKILSQSGGMAAISTCEGDGKMHGLLVIPEGKYTMQPASSVQPHLDIPIERESVPHVLLKNTEDIEKFCAADHIAPPVDIEDNEIPNLHVRRRRSKPRYTIRMAVFADKSLIDHYSDKKVNVKFAILNIINQVQFIYKYDSLKTQIDIRVVKLDLNVAEKDVPDNANGDIDRYLDNFCLWQREKKRRSKENWNLAVQLTGINLFKLVGSSLNKKVLGLAWVNGMCRDRYSCTICEARSFEAAYVMSHEMGHCLSMMHDGSNNSCNPDKFIMSPKTGPGKTNWSPCSNRYLEDFLRMPQSSCLLDDKNARDLTSEMDPRLPGQKYPPRIQCEYALGKGYKIYNNSKAPYNNICRELWCVQGYWATPAHPALEGSSCGQDKICLQGDCIRKLKSIPKRPSSTERSPVHSKVANNNENQSPTFLDKIKGFVRNVFDKYFSGK* |
| CliEnzMtp07 | XP_023243712 | 0 | Metalloprotease | MFIQTVLIILSVLWPIGHVGA**TPQIHKVMTRGELLRTFQVSSANDVPVYEVVNIRSLTKREISDGEIKRIYLSAFGKKMKLNLKKNKDFEERLKKFKVLLAETTNNGLKYRDVTAQQGGSSDLGSTYHDDEKMAAILMRQADDGGIEMEGTIGNDFVIKPIPKTLYDSSYVDDEMFLDEDEQIELNKTRANFNNSQNIGGNHIVFKRKFGGLHDHSDYLNMESASLLNTSKLHQATSSRRKRAAPDTVWPEVLMVADYDTFLLHGSNSRDVKRYFVSFFNGVDLRYKLLSFPRVRISLAGMIVAKDRDATPYLERNRLRHPNHDAVDAAGALTDMGKYLYRENRLPTYDLAVVITKLDMCRRRFEGGRCNRGTAGFAYVGGACVVNKRLEKVNSVAIIEDSGGFSGVIVAAHEIGHLLGCVHDGSPPPSYLGGPGATHCPWEDGF** |
| CliEnzMtp08 | XP_023234770 | 0 | Metalloprotease | MSFAVIFFGLATLWICGES**KFEKFHERLSQEELRKIFHVDLHNNVPEYDVVHVRSLSKRSIPSDDTKRVHLSAFGQNFHLNLKRNHELEDRLKSMKLFAAETKGNELRYKEMEPQEHAISDMGVSYHDENQMAALLVNHAEDGSLRLEGTIGNHLVIKPIPKQLIVIEDNYVDDEMFLDEDGNTSTTTPNTQRHLPTLSSVNHVVYKRKQNVPLSHSDYMELEAGYANNSDWTIRSRRTKRKAPTTVWPEVLVVIDYNTFLLHGSDSRALKRYFVSFWNGVDLRYKVLANPKVKISLAGMVVAKDKDATPYLEKNRLRPPNYDAVDAAGALSDMGKYLYREDRLPTFDLAVVITKLDMCRRQFNGGRCSRGTAGFAYVGGACVVNKRLEKVNSVAIIEDSGGFSGIIVAAHEVGHLLGCVHDGSQPPSYLGGPGASNCPWEDGFIMSDLRHTDRGFQWSACSVQQFKHFLQGETAVCLYNYPHDNQILSRVLPGTTMSLDDQCRKDRGTTACFKDARVCAQLFCYDTSSGFCVSFRPAAEGSTCGHGQVCRDGRCVGETENIIPDYTHYTPTYAGRHPNFVYRRTDRRGTTPYISRTTREIITRTTPSQSPSSCEDNAQQLAGGLTCTEFLQRYGFRYCKHRYMKQKCCKSQLKLCSHS*** |
| CliEnzMtp09 | XP_023234515 | 0 | Metalloprotease | MLIYLISFSLCVVISA*IPSGRIDVVYPSVETLRSGIKMIKFRTFDRDIELKLEPASEVISNDFAVVSDDQKQKQINIEFLKRHLYRNKEKGAALHINEEGPLSINGILSSDLRIKPLDKENIDKYGNRAHRIIEVLQDQR***SRVISKIVTPEIENRTYNRKFSRISEDECVELKYIFLTESRFTKAFENDTVTLEEYLSVTLIQVQNFMDTLDLRIKVRLVKAISFTENSEPEFMKNSAIKGHENYIDPNKLIGGARDYLCQSRENFNTGEADIIFVISKRRFGQMASDGSISTGIAGIAYVGVICDECYKTGCVFDDTNIYERSDTIAHESCHILGSPHDGQGPVSHVPNSPGSENCPSSYGYIMGSRNEANGTKFSPCTRNNIANLLKLGGRDCVKTKCTEE*** |
| CliEnzMtp10 | XP_023213460 | 2.00E-123 | Metalloprotease | MNCLITFAVVFSIIIVDTYG**KSFGLKHLKKLSVTEKRTVDVADAEDFEIDSTPPCPVIECVVVTDSFVYKHLSEEGIRPYLNELIRVSQEKYLDELGLNLTLRLIGHISYTNETEPGFIKGSLHSPTMIVGGSIIYSMHAINDNPLVSRADVVVLITGRTLINPFPKPGEEPQYIAGLAFLGTACNPSYKFAILQREGDQLYIDYTASVFIHEVGHLINIPDDWNGKDCSTALLPGKKHFNYCAKKDVIGFLDTHSCFLDHCDNGQE*** |
| CliEnzMtp11 | XP_023216482 | 9.00E-143 | Metalloprotease | MNCLITFAVVFSIIIVDT**YEKSFGLKQLRKLSVTEKRTVEDVTDVEYYEIDSTQPCPVLECVVITDGYVSQQFSEERIKSDVKETIRLIQEMYIDELELNLTLRLIGHIFYTNETEPDYIKKSLYNPKTIAGNIIYNMNAIKDHPLVSRADVVVLITGRTLINPFPKPGEEPQYIAGLAFLGTACNPSYKFAILQREGDQLYIDYTASVFIHEVGHLINIPDDWNGKDCSTALLPGKKHFNYCAKKDVIGFLDTHSCFLDHCDNGQE*** |
| CliEnzMtp12 | XP_023234498 | 0 | Metalloprotease | TKAVIKAISSMELRGLKIICILFYFVTISAVLS**GGIDVIYPSVITLRSGEKILKFRLLEENVELRLEPAGKVISDNFTVVNGKGERDESIDVKQLKSKLYKNEKIGAALHIDEGESLKVHGIINSYLRIEPFGPEEMVRKGKRAHHIIELKKDKNVYIKDQVIPKKLEKLLNPKARILNSNECLIIKYVFITESTFTESFIERNIGLTDYVATMFVQVQNLFNTLELNIEICMVGILAYTKLDEASFIQQSVIPGYEEYLNCEDLLDNMAIYFYSNPIEYRHVEWADIVILLVKRKWGDLINGDVYANTAGISYVGGVCDGRYKYGVCEDDANFMERADTIAHESGHLLGCPHDGEGPAPGIPHSPGAKECPESQGYLMSSNQSKENGQKFSPCCRKNVQNLLLWIETAQSIIQICKLDGDYNDNYYYNDEDYHVDNYDADND*** |
| CliEnzMtp13 | XP_023234503 | 0 | Metalloprotease | DAALYINEEGPLEIKGLIDSRMKIEPCSSNEENKEGRKAHCITRRSEDDKRIHDAVIPPSIHQIYSSESLMMMQDDQCVGLDYIFVTESNFTRHFPNSEEIKVYLTMALVGVQNIMDTLNLRIKVRLIGVTEYNEENEPNYIKQSEMPGHKGIISVNQLIGNMMDYFCQHQNDKGFEEYKKANIIMLITRRTFGNLYPDGNLFTGITGVAPLGAAACDSCGKCGAVTVGDDPLYRDEIIAHESAHLIGCPHDGEGPELSLPGSPAAKDCPGSDGFVMGDQKGENKRKFSNCSRENIKYFLSLPTSQCIIRDCKK* |
| CliEnzMtp14 | XP_023234510 | 0 | Metalloprotease | FCSPVLAIPAGRTEVTYPSLETLRSGEKILTLRAFGNDMELKLESAGDVIADDFTVMDGDGKIHKTDVKNLKSKLFRNKEKNAALYINEEGLLEIKGMLTSKLRIEPYESQDTLRDGIKAHRITEILVETQRFNDAVFSSNLNLYSDYEAREFNEDQCVEIEYLFVTESTLTKRYPNLEKLEIYLASMFVKAQNMMDTLNLNMKLRLIGVKAYTDENEPAFVKESLFPGKDIFQYNRILRKFSNYYCVLRDDELVNRADIIMFSTRRAIGTLKDEDSINDSILGIGYLGGSCFRCTKFGVIKDLDDFLNTADTVAHESAHLIGSPHDGVNSSLSGSPGAEKCPRSDGFIMGDNSGNNRNKFSTCSLENIKFFLNKKQASCIIADCKKTF* |
| CliEnzMtp15 | XP_023234510 | 0 | Metalloprotease | FCSPVLAIPAGRTEVTYPSLETLRSGEKILTLRAFGNDMELKLESAGDVIADDFTVMDGDGKIHKTDVKNLKSKLFRNKEKNAALYINEEGLLEIKGMLTSKLRIEPYESQDTLRDGIKAHRITEILVETQRFNDAVFSSNLNLYSDYEAREFNEDQCVEIEYLFVTDSTVTNWYKDAEKLLAYLFPMFVEVQNIMDTLNLSMKVELIGVRTYTDENEPAFVKESLFSGTDTFEKKAIINNFSNYYCKRQYDSFANRSDTIMFITRRAIGEKYYGQIIENILGLAFVGGACDPCKKYGVIKDSDNPLNMSIPAIVAHESAHLIGSPHDGEGTFSSLPGSPTGRKCPASDGFIMGDISGNNRNKFSTCSLENIKFFLNKKQASCIIADCKKTF* |
| CliEnzMtp16 | XP_023234510 | 0 | Metalloprotease | FCSPVLAIPAGRTEVTYPSLETLRSGEKILTLRAFGNDMELKLESAGDVIADDFTVMDGDGKIHKTDVKNLKSKLFRNKEKNAALYINEEGLLEIKGMLTSKLRIEPYESQDTLRDGIKAHRITEILVETKQFNDAVFSSNLNLFSDHEAREFNADQCVEIEYLFVTESTLTKRYPNLEKLEIYLASMFVKAQNMMDTLNLNMKLRLIGVKAYTDENEPAFVKESLLPGKDIFHVDYIFKNFSNYYCRRQDDELVKRADIIMLSTRRVLGKPNKDGSIFYGIVGVAHLGGTCSPCAKYGLIADLNNFLETADTLAHESAHLLGSPHDGAGFFSSLPDSPTGEKCPASDGFIMGDISGNNRNKFSTCSLENIKFFLNKKQASCIIADCKKTF* |
| CliEnzMtp17 | XP_023228637 | 0 | Metalloprotease | MNILRILTMLIFLLTVSIFIVVSG*RLTGREEIVFPSIETLRSGEKRLSFRALNEDIELKLESAGNVFGEGFFVESIHDGRSTIPELNLHDIKQNLYRDKRTGASLHIEEKEHTLIRGIINHKLRIEPHESEARIRGGVRSHRIIEYIEEDGFHENDAVKPGANFHANINKSADDLR***SGEPIYVEICIVTDTAFTKLFSNDYAIVLYVAILINGAQCRYDNLKNIRVILVFSGLKIVRYARDEPYIQNNLVEGHLLKGFDALNDFGTYMGRAGYTCDVIKLLTKLDLVDEVDGKLASGLLGLAYIGTVCDTSSEPHFSYKVGISEDDAHCFAGVRTVAHELAHSLGCGHDGDSPLSYIEGHPGSKNCPWDDGYIMSYVQESLNRYYFSSCCEDCIRYSAMIFDCLRNNNTGYNLGVSSEIPGVQMENTLGYSASKYENEKVAYYDHRCRNLKRGDFFLLSFKYPDGTCSYACRTEVNEENQYYYWIMDCLDGDMCDTGKRCFHGQCI*** |
| CliEnzMtp18 | XP_023240574 | 0 | Metalloprotease | MIYLASIFLFAIASA*IPSGRVDVVFPSVETSRSGVKTVKFRALNEEIELKLEPAGEILAENFVLFDANNRILPSIEVQDLKSKMYRNSTNGAALLIDEEGPVKIEGIVNSNLKIEPYESGRAVIDGRIPHHIVEVTSDKNSFGHDGVMPMGVKETTKKVERMAR***DNQCIVVEYLILAESNFTKHFKTNRGLVNHISRLFIQVQNMIDTLELGIKLSVLGITMFTKKTDPSYIQESVIPGYEQYLHAMELLSNMGRYYCHHATGLAVDADIIMLFVHRKMGSLNPDNTVKLNVLGVAHTTGVCKKCRKVGIVSLDPLNYHRANVVAHESAHLLGSPHDGEDAKEEGSPGGLNCPKSDGYIMGSRRNGNENKFSKCSKESIKYTLSKSSCVIEDCKF*** |
| CliEnzMtp19 | XP_023234508 | 1.00E-179 | Metalloprotease | MVFYFAIYFLFGVVSA*IPTGRVDRVFPLVETSRSGVKTVKFRALNEEIELKLEPAGEILAENFALLDANNRIRHSIDVPDLKRKLYRDSTNGAALLIDEEGPVTIQGIVNSNLRIEPYESGRTDRDGRIPHQIVEVISDKTSFGNDGIMPVDVKKTIEKVKRMAR***DDECIVVEYLVLTESNVTKRFKTDKSLTEYISVTYTAVQNIIDTLELGIKVRVLGITSFTEETEPDFIEKSAIPGYEQHLDPREVIYNMRMYYLEPATGLAADADIIMLLVDRKMGILHDDNTLSLNYYGMAYLGSVCSSRYKVGVTRHFSDHIERAAIIAHESGHLLGSQHDGEEPKGVDCPGSDGYVMGGRSTDKKNTFSSCSKKAIKDTLSLSRSSCVIEDCKF*** |
| CliEnzMtp20 | XP_023234500 | 0 | Metalloprotease | MIYLASIFLFAIASA*IPSGRVDVVFPSVETSRSGVKTVKFRALNEEIELKLEPAGEILAKNFALVDQQPNTIDVEDLKRRLYRDSDKGAALLIDEDEPLTIQGIVNSNMRIAPYESGRMVKDGRIAHQIVEVISDKNSFVNDVVTTDINRKLENVEGR***ADKKKCIVIEYLCVTESNFTKRFNTDKALTEYVTLLFTGVQNLLETLNLGIKVRLLGIQTFKIGTEPSFIKDSAIPGHEKHLDPVDLVVNMGKYYCKNDTGLAKQADIIMLIITRKLGELEDDGTVAFSTAGISQGGGVCIQCHKVGVAQDDSDYNERVDTVAHETAHLIGSPHDGEGPEEMSIPKNPGAKNCPFNDGYIMGSDDHKVNKFKFSQCTKKCIEHLLSLPRAACLYKDCK*** |
| CliEnzMtp21 | XP_023214049 | 0 | Metalloprotease | MIYLASIFLFAIASA*IPSGRVDVVFPSVETSRSGVKTVKFRALNEEIELKLEPAGEILAKNFALVDQQPNTIDVEDLKRRIYRDSNNGAALLIDEDGPVTIQGIVNLKFRIEPYESGR***TVKDGQVPHKIVEVINDGKSFLNDAVMQMDVNEEMEKVATMARDDQCIVVECLVVTESAFTKRFETTKALTEYVTVMYTGVQNLIDTLEMGIQVRLLGIEPFTEKTEPPYIEESEIPGHPQNLNPYDLVEHMGKYYCSHATGLAKDADMIMLLVTRKLGELQDDGTVAFTLAGIAYVGTVCNECYKVGISLDDSELIMLIITRKMGELKKDGTVKFTAMGLAYLASVCKKCYKVGVSMDDSRYNERVDTVAHESNHLLGSLHDGERSDEGGPAGNPGAEDCPQTDGYIMGDRSDIVNRYKFSECSKKCVKYALSLPGASCVYESCGF*** |
| CliEnzMtp22 | XP_023214049 | 0 | Metalloprotease | MIYLASIFLFAIASA*IPSGRVDVVFPSVETSRSGVKTVKFRALNEEIELKLEPAGEILAKNFALVDQQPNTIDVEDLKRRIYRDSNNGAALLIDEDGPVTIQGIVNLKFRIEPYESGR***TVKDGQVPHKIVEVINDGKSFLNDAVMQMDVNEEMEKVATMARDDQCIVVECLVVTESAFTKRFETTKALTEYVTVMYTGVQNLIDTLEMGIQVRLLGIEPFTEKTEPPYIEESEIPGHPQNLNPYDLVEHMGKYYCSHATGLAKDADMIMLLVTRKLGELQDDGTVAFTLAGIAYVGTVCNECYKVGISLDDSEYNERVDTVAHESNHLLGSLHDGERSDEGGPAGNPGAEDCPQTDGYIMGDRSDIVNRYKFSECSKKCVKYALSLPGASCVYESCGF*** |
| CliEnzMtp23 | XP_023229533 | 0 | Metalloprotease | MIYFAIFFPFVVVSA*ISSGRVDLLVFPSVETSRSGVKTVKFRALNEEIELKLEPAGEILAKNFALVDQQPNTIDVEDLKRRIYRDSNNGAALLIDEDGPVTIQGIVNLKFRIEPYESGR***TVKDGQVPHKIVEVINDGKSFLNDAVMQMDVNEEMEKVATMARDDQCIVVECLVVTESAFTKRFETTKALTEYVTVMYTGVQNLIDTLEMGIKVRLLGIDPFTKETEPAYIEESAIPGHPNYLYADHIVDRMGKYYCSHATGLAKDADLIMLIITRKMGELKKDGTVKFTAMGLAYLASVCKKCYKVGVSMDDSRYNERVDTVAHETAHLLGSPHDGEPKAEDCPDTDGYIMGNRRNATNKFKFSECSKNRVKYTLSLPGASCVYESCGF*** |
| CliEnzMtp24 | XP_023209358 | 0 | Metalloprotease | MLSAFLLCLSSINSC*KYLITEEQIASRFIR***NVNQINERQCHLFQLASWNYETNITKYNKNKMIEQEMLNSKITKENWKKLMKFDWKHFSDPQLRRQFHHLSHLGDEALSVTKLRKKSSLEADMVDIYSTTVICDFKNKNKCNLALDPDLTNILAKSNDYEELKHVWKEWRNKVGRKIKPLYWKFVHLSNEAALLNGFKDAGEFQRDRYESPTFLQDLENLWKQIQPLYQQLHAYVRRKLIQKYGKDKIKHDGPIPAHLFGNMWSQEWQNILNLVIPYRNKPSLDVTPQMKAKKMKPIQIAKLAEEFFVSLGLRPMTKEFWNNSLFEKPKDRKVVCHASAWDLCNKGDFRVKLCMEPTMEFLITTHHEMGHVQYYMQYAKQPHIFRTGANPGFHEAIGDVMGLSVYTPNHLQSIGLLNDIISDRESDINFLMEMALKKIAFLPFGYILDKWRWGVYSGEIPLEEWNTKWWEMRFKYQGICPPVRRTEIDFDPGAKFHVPANTPYIRYFISFIIQFQFHEALCRVSGYLGPLHKCDIYNSKEAGKLISEMMQMGSSRPWPEALEKLTGTREMNAHSLLEYFKPLYEWLKETNKEEIIGWETIDPNVCP*** |
| CliEnzSeP01 | XP_023238707 | 0 | Serine protease | MKRCLQFFLLLLLLFPAIPG*TRRGASRRGRNCRFRR***PGDGKCVSADFCGRIGKAGTCPGSGRNSPVCCPRVHVRRRTASGVTPACGLNLPNASRLLTRIRQQVVGGRDSDPAFWPWMVSLHKKTSNSTRYLCGGALIDRRRLITAAHCFGRKDPKASDYLVKVGGRDEGRERRVESVRVHPGFSNARYYHDIALVTLEEDREWKTMPACLPDRNEDYSGKEAVLIGWGDTSFGGRPGRVLQRVTLRVFANRRCQADYRKLKSAPFPRGIVPQLLCAGHRREAR***DACQGDSGSPLMLRDSSSGTWKVIGLVSFGYQCAVSAYPGVYTRVDHYRKWIAENGKSS** |
| CliEnzSeP02 | XP_023239597 | 0 | Serine protease | MFKLDRRKTLLLMFAVSAVRA**QQSNDAQEGHSGHQCDCMEYWECISAGGKPYSYCVYSNKVCCFIDQNAKTVGILPRRGKSTSCGQKGVDNRKDGFSEPGEWAWHAAILEKPRDLYVCGASLVDEYWVMTAAHCVDDFVDSKSLKVRLGEYDVLRSSEPQKHEEFQVSRVIIHPGFVNTTLLHDIALLRLSTPAKRRSHINTVCMPGNELSEKVLTASKCIVTGWGKRSERGNHSVVLKEIIVPVWKNNDCERSMKLQFGPNYQLPSSVVCAGTTGRDACDGDGGGPLVCEKNNHWYQIGIVSFGIGCGRPNVPGVYTRVHSYKQWIHDVVLNS*** |
| CliEnzSep03 | XP_023233218 | 0 | Serine protease | LISEISLSLRSFAIYFDFNTMKFLGWQLLCCCGFVFT**LTIEDENCGKRNISDEELDFKIVGGLPARPGEWPWQVSVQLTHPQYGKIGHWCGGVLVDKRWVLTAAHCIINSLFALPQPVFWKIRLGDYHLKKTEGTESTIAVSDVYYNRWYLGYQNDLALMRLSEPAKIDYYVRPICLPTSEDGFEDMTCTATGWGKADFNRTGSSTLQKVSVKVMENSICLNAYMKNFNISILPSHLCAGDLAGGKGTCLGDSGGPLQCLIKDKWYLAGLTSFGSGCAKPGFPDVYTKVTYFVDWIKHIQHTHIARF*** |
| CliEnzSep04 | XP_023227283 | 3.00E-172 | Serine protease | MLRPFQSSYQCRTPDGLPGVCSVVTQCIYLIFDLNKLRQSVCFRPFSMPGVCCPVIRDVTSTVATATTTDKLDPITTTTITYDNFTSSLPSTTVIWPTDVETTSTTVTTTTTDTTTVAVNETLSDLSLFKQPIIAESTRGCGHSGRDGRIVGGFESEAGNWPWMAAIYLITHRGREYWCGGTLIDNRHVITAAHCLVHKSGRKYHPRSIIVRLAAHRIYNHYDRKNTVDVYVTKTIPHAKFMRHGFYNDIGIIQLASEVKYNIDISPVCLPSIKMADNNLEGYMATVLGWGVISYGGRNAETLQQVSVPIWNNVDCQKRYVQKITKGMLCAGYHEGLKDACQGDSGGPLMVPNNSRRWILVGIVSFGSKCGEKEQPGVYTRVTNYLDWIHEVTKA* |
| CliEnzSep05 | XP_023210062 | 0 | Serine protease | MKFHLILICLLSSKKISG**IEVNFPEETKRCHEKLGEEGICIEFGKCSIAKELIKQRKIPVPCGFSGIVPLVCCAISSLPGQLVRESECGIKQLEEEERNSGISFPSTESSEYGDSIIQPRKLNRTLENKHEFQHGNLPWIVSVVRKNDSAVICLGFLIDEFRVITAAHCYSEASTAEHYSMYILKNGSKEWYEIDEVRIYEEFNGYYDDIAILKSSSAISGIPICLPKTINEINLIGRAAVFIGWLNGDDDDVKMVSEEMQIADVEVCEKDYSKFIVSPFPSGIKEESICTYSLYPDADTCKNNSGGLLMLEDEGRWFVIGIQTFSAPCDLQNIPIVYTNVSHYLNWIQ*** |
| CliEnzSep06 | XP_023209481 | 0 | Serine protease | MNITLVFALVTFKQAWS**VSIKPKPCFNGKRQRGTCMFVWECIRAEGKHLGTCTDSFLVGSCCWRRGNAEEQPPAQWTASHFHNSSSENGVRPTETDRRKKPRPECGVPFDTHRSKVVGGSDAAFGSWPWQVSIRKVRGSYGFASTHRCGGVILNRLWIATAAHCVEDLYPSRMRIRVGEHDFGSTNEPFPEMEKNVGGKVLHPGYNVFTYEHDLALVKLADPLVFRPHVFPICLPSTDDAFAGREAVITGWGRLREGGILPSVLQEVKVPIISNQRCREMFRRAGKLEVVPETFLCAGFAEGGRDSCQGDSGGPLQIKDDDGKWILAGIISWGVGCAQPNMPGVCVRITKYRDWITGIIA*** |
| CliEnzSep07 | XP_023237211 | 0 | Serine protease | MADVLQIFLIAVFFLIEYSMQEA**DFENGTPCGRQFVRNGKIVGGRNAEEGEVPWIASIYLRGIYSCVGSIIAKRWILTAAHCFMSTSKPRNYYIRVGNLYMNGEGNDLDIESIYKHPNYRNPRRYNNDIALLKLKNDIVYNKYTWPICLDRNNVSLNDKIATIAGWGKLEEGGAYHPEILQLVRLPIVNNEMCQQWFRNNGKKWVIVEDNQICAGYEEGGKDACQGDSGGPQYIKQGNSHILIGVVSSGIGCARPQTPGLYTRVSSYISWIDETIKNNS*** |
| CliEnzSep08 | XP_023230910 | 0 | Serine protease | ERSISSFPRGLVNFSLLKPVRAALFSDCTTGSQCQLTLTCWLSGGVVTTPCGPFFTCCSSAAEQKIQPAYYGPVRNDPYCGRNSENTRRIVGGSDAAFGQFPWQVFIQIGGSRCGGALVAWRHVVTAGHCVAKSQFNPSNIRVTVGDYILNSDMESIPSESFGVERVKLHPNFRFTPQADRYDVSVLILDRPVSYKYNMKPICLPEKNADFLGRIGYAAGWGALEAGSKLRPKVLQYVPVPVINNQICETWHRRRGINIRIYDEMLCAGYEFGGRDSCQGDSGGPLMINSYGVWYLIGIVSAGYSCAKQYQPGIYHRVSSSSDWISSNLY* |
| CliEnzSep09 | XP_023228456 | 0 | Serine protease | MKILFSSRHSLDMKYLTIFLAIGYAIA**QDEQFGGSEGGTDVSPDKLGGADYESAIPNPGLSTNPDPIPDQSADCLCVPYYQCKDGEIINDGSGIIDARKKPPLKEELPLDKHFEPPFCGAFHVCCKAPESSTARPYEHRCGVRNPSGINSRILSPGKKGESDFGEWPWQAAVLKVEGKVNLFQCGGVLIDKRHVLTVAHCVFHYKGLNQYPLKVRLGEWDTQNTDEFLAHDDYNVEKIITHPEFRNNSLWNDIAVLTLDRDVIFVPHIDTICLPNYKDIYEGQTCVVTGWGKDAYKGGTYSNILKEVNIPVLDNPKCQELLRETRLGIFYKLHEGFICAGGEKGLDSCKGDGGGPLVCYRPDGTYALAGLVSWGIDCGLAGVPGVYVRVLKYVDWIVQNTGGRLEDYWPIGS*** |
| CliEnzSep10 | XP_023228456 | 0 | Serine protease | MKTFTAVVVLSVLSFLHA**QNENNYKRRARVFLEPLTTENTTEKFQPIISSNNETDIETSVSATNDTEGRSIDLDLFDFKGSSRQGRIINDRKKVKVSGFIPLVTLDDVDKSEETPQESIYDYPIYKPEKSALPPIINAHKQFISQGDSYPSDVQANFDNRKLSPIYPGTPGKYPSSSIDRPLYQEKVVDTHGPIYPHPQSHHFPDKGPKYIEHVHRPNINQLIPKRDQCICVPFYLCKNGFLQDFGRNKDLVDERSANVGAVYFDQKNHTQNLEDVAQERIVNDKTNATKHQTDLADYASDVMARFIGLPDDGCGYMRICCKIPPVISPVGSFPPLHPLHPNPIHQVIPEIHHPHPNPVNKPLPPIVRPPLFPNPNIGLPKPKPVYPGEIGIHPGHFHKTCGVRNAVGIHGRVQNLQYYDDSAEFGEYPWHVAILKKTGPSESLYVCGGVLIHPQWIATAAHCLKKHGPDDIVIRLGEWDVHRGDEFYPYKEKYVADIIVHPHYFPGNFANDIALIKMDSPMDPKLPHITPACLPEPHELFVGQRCWVTGWGKSAFGHKGEYQSVLKEVDVPILSHKDCEHRLHQTRLGPYYRLHPSFVCAGGEPGKDACTGDGGSPMVCEVHGIWKVVGLVSWGIGCGIPGIPGVYVNMANLRPWIENIIHKIG*** |
| CliEnzSep11 | XP_023230909 | 0 | Serine protease | MRRIIGDAKIYILLVYLVYINQRSNA*QLFSFRRR***ADTDTCHRNGEVHQCQFFLFCLLSGGTSAGNCRGNLLSTCCVKPNLRRSRPSNFNQRTLRKETNSVCGRSAVKPRSRIVGGQDAFYGEFPWQAHIKIIQQQCGGVLVSPYFVVTAAHCVYRARLPQITVVLGAYDIHDHSYQLQPAHYLKVAEKRLHPKFKFSPSHPDRYDVALLRLNRKVNYQENILPICLPPYGWNFRGWRAVVTGWGKTDPALRNRYGTRLLQKVDVPIISNQECEYWHRSRGIQLKIYPEMICAGYENGKKDACVGDSGGPLMVNMNGKWTLVGITSAGFGCAQWRQPGIYHSVSTTVDWINSNIR*** |
| CliEnzSep12 | XP_023239874 | 2.00E-125 | Serine protease | EEEYLPYVERRVQLIASHPKFDRRTFEYDLALLRFYEPVPFQQNILPICVPTGNSTYIGEFATVAGWGRLYEDGPLPEKLQEVEVPIVANKECEDMYRKAGYVEDIPDIFICAGLAKGGRDSCEGDSGGPLVIKEDDGRWVLAGIISWGIGCALPNQPGVYTRITKFSDWINQIIIF* |
| CliEnzSep13 | XP_023243443 | 0 | Serine protease | MIEFLLCILCLSSVLKTSLS*LDTCEVSSEVSGMCRRRSDCSTLRRGSVVKSCPERRDLICCPLWRKTTWPVQTTKRSRAQSKTTAIPRTENVCGQRSVTRRRSNR***GVDLEEEEESFNLTTDAIVNGRTAVKGSWPWMAALLSIRTTSFGQSASFFCGGSIITSRVVLTAAHCLYPYMHNRRQISSNMKKLKVRVGAHTKSDGISHDISQMNYHKSYGRNYANDIGYVKLSKDIIFNAHVRPICLPTLIESYSDTFYITGWGTTRSGGSASSTLREAKVKMVSLSTCKSSYSRINLKITDKQVCAGRGEADTCQGDSGGPMVMYSETKKRWILVGITSFGNGCGNRRYPGVYTSVKAYRSWIDTIS*** |
| CliEnzSep14 | XP_023239572 | 0 | Serine protease | *FRKRR*LLLVFLGTMSSAFVLTFFSLLICVDLSAVQDEKRIFGGRFANPGEFPWMVFIRLTDELNCSGFLISPSYVLTAAHCMIRPLEEMWAKIGTVDREEGQEYRFQSSRVHPDYSNLTYHGDIALLKLTSPVVFDQNINRICLANDRNFYRGNTPVLQMGWGRFSNETAEVTRILKVTEEGYIFDHGDCSDMFAFFNYTLWDGTVCIKNSGSEGVCEGDSGGPLVTRNGNSYTAIGLESIGFYENCTVDNSFAEVFTDLLYHRQWIVDNVDETICQQ* |
| Phospholipases | | | | |
| ID | ID reference protein | E-value | Sub-classification | Amino acid sequence |
| CliEnzPA201 | XP_023236869 | 7.00E-81 | PA2 | MDKCCRTHHFCKDVIPVLSDRYGLTNTDIFPKLSCDCEEKFFKCLHKANTVVSNKMGETYFNQEKSQCFMKDYPIVKCLIRKAISQYESICVKYELDATKSEIYQLFDTPFYKVPNFHRE* |
| CliEnzPA202 | XP_023234362 | 2.00E-161 | PA2 | IVLEFSMILLLLLCQTTHF**VVVLQSEIYYKHDWSKISVEFPSSPCEENGLKCSENKSWKFSNSISNKQNDIRRKHLRQIPYENIKKRYKRSQDDFIKTFAILPGTKWCGAGNKSDGGDLGYFNDTDRCCRRHDSCPNSIQSKATKYGLRNNESSTMSHCDCDDEFYHCLKEVNSVVSNKVGIMFFNVLQKSCFRKDYPIVGCKNKSWFARRCKEYEFDRSKRKIWQIFDAKEY*** |
| CliEnzPA203 | XP_023234380 | 1.00E-166 | PA2 | MLRSLLYILVLVEISISES*QVYVYPYGSKLILVEPNHDPTKTSRNCYFYEDGKIGNSIGENSKDVEEEIIRFLKCETATTESKIRSKR***TINKVLRKLVIFPGTNWCGAGNVSNNARDLGTFEKTDDCCRNHDQCFDVINANQTKYGLYNAGLVTLSHCDCDDEFYECLKEVNSPASFSIGNLFFNVLKMKCFREDYPIKGCIKETGIIKVCQEYEFDVEKPKEWQMFDPKIY*** |
| CliEnzPA204 | XP_023231606 | 3.00E-105 | PA2 | MLSIIKNFILVVLLLSTCQICEF**RSLGRKERSLIDLAEMVKITTGRNGTDFVPYGNWCGMGGSGKALDPIDDCCRRHDLCYIDKLGKECNSIFNLYVANYKWNNAGGKISCSIEDTNPCNRATCACDREVVFCLAKNIKEYKEEHRYVRSALKT*** |
| CliEnzPA205 | XP_023242353 | 3.00E-102 | PA2 | NIHFKEIALHYVGTKWCGAGDVADDYDDLGPAAETDMCCRTHDHCNDSITGFETKYKLKNKDFYTKSHCDCDNGFHQCLLEGETLISDAVGHLFFNILQTQCFKNEYPIVKCLKKWGIPIVRDICQEYELDENKPKKYQFFDGKMYQGKHEPSFLKNILSH* |
| CliEnzPA206 | XP_023242353 | 0 | PA2 | MYFLIIFGGTMMSILTTTTVAG**GILDIVDDILPVTTSFYREKDGHRMVEIIEVNTYIGGKKLVDCFLYGDNYIIEKMLELVPKKLVKVVPKKDISKLVNQCNELLYRKIRENAFDIIKTPFDFARKIFKSFLIFPGTKWCGAGDVADDYDDLGPAAETDMCCRTHDHCNDSITGFETKYKLKNKDFYTKSHCDCDNGFHQCLLEGETLISDAVGHLFFNILQTQCFKNEYPIVKCLKKWGIPIVRDICQEYELDENKPKKYQFFDGKMYQGKHEPSFLKNILSH*** |
| CliEnzPLD01 | XP_023237656 | 0.00E+00 | PD | *MNSSEGRLTSLSSSDYDDYEDLKPPDSEDEIDHNESREGPPYIPFAHIHAPSLGFEDLRLTMLIPNRPIRLKIIDAFRVPGSSIMNPNLYVIHLQHGNFEWIIKKRYKHFQRLHQQLLLFRASLSLPIPTRRYRERRKSFKHRKTLPLFPRRPEALIQPDQLSHRAEQLEKYLRNLLRIPLYKTHYETMNFLEIGPLSFINDLGQKGKEGLVLKR***SGGHLTKAVCLKLRRAITECCGFWRKRWLVVKDSFVAYIRPKDGKIKSVLLMDSYFNVECGLAATGIHHGLFITNLSRQLLVKCWTKRKAREWMQHIVETSNTLARDFTQPNRFDSFAPVRFCVDCRWFIDGGTYFEAVADAVERAKVEIFIADWWLSPEIYLKRPVIQGELWRLDRVLQRKAEEGVKIFVLLYKEVELALGINSYYSKKQLAQLHPNIKVLRHPDHVTGGVLLWAHHEKIVVIDQTYAFLGGIDLCYGRWDDYLHRLTDLGGIHKPVQNKSGYTPPPRRCCSTSDLTMAAHDSEKFMYQAKEMQYNKLHRHDSAESLPNFDVTITAPRIEIDEDIKNVTFGDNFNTQEDDKYKKKEKDVPDGSAISRPRFTTKLKTQRVMQAVARFQALKHRLHHKGHSIDSLRMGARTDSLGIPSFELRRTASEVALNQMGLQGSCKLWFGKDYSNFITKDFVNLDRPYQDLVDRTVTPRMPWHDIGVLVQGSAARDVARHFIQRWNFTKLEKAKNYDAYPYLLPKNYENVTNIPPLPLSSVGILYTTNCQILRSVSTWSAGIRTTERSIHSAYVNLINNAKHFIYIENQFFITQAAGHKDVFNEIGEALYQRIMKAHKNNETFRVYVVMPLLPAFEGEIGTTTGTAIQAITHWNYASICRGPGSLIQRLSTEIDDPISYISFYGLRNYSTLNDKLVTELVYVHSKLMIVDDQAVIIGSANINDRSLLGKRDSEIAVVIEDIEFEKSLMNEKPYNSGLFAGSLRRSLFKEHLTSLNKDKSLDIDVRDPISEHFFKDVWMKTAGVNTSIFEKVFRCIPADEIHTYSQLRQYVSQSGLCETDPDSAKELLEKVKGYLVLFPLYFLCSENLTPAHGTKEALMPVSLWT*** |
| 5´ nucleotidase | | | | |
| ID | ID reference protein | E-value | Sub-classification | Amino acid sequence |
| CliEnz5Nuc01 | XP_023233208 | 0 | 5´nucleotidase | MFYKSEMWFAVLTCMFTLLSS**IESYNLTVLHTNDFHSRYEEINNKGGKCKPEKSCYGGIARQVTAVRNIRNAEENVIFLNAGDYYQGTVWYTVHRWRAVAEFTNRLHHDAMALGNHEFDDGVAGLVPFLDNVTFPIISSNINISGIPELEGKIRKSVILDVGGEKIGVIGYTTKDTPELAKTGPVTFFDEVESIQEEANNLQQSGITIIIAVGHAGFLKDKEIAEKVPLVDVVVGGHTNTFLYTGEPPIPQEKEGPYPVVIERSDGSKALVVQDYAFGLYLGHLKVTFDETGNVISWEGNPILMDETIPQDNETAEVVETYKEVVDRRGNVIVGQTNVYLNADRPYCRMHECNFGNVITDAVLRFYLKKPTENQWNTIAISIFNSGGIRDSISEKEHDGNILMRDVMNVLPYLNTLDVVDIYGKYLIEILERSVYDYGNNPEDPPGRFLQVSGVRVTYNISQPPGQRVHKALVHCTFCRVPRYLPINETKIYRVVMPTYLTQGGDDYKMIPENTLRLLNTGSLDIDIVVDYLNKSSPIVTGIEGRINFVDPYAPCNGAALSTINNNSLCIYAFLTFSILLFPGVHKNLPVIATI*** |
| CliEnz5Nuc03 | XP_013794928 | 0 | 5´nucleotidase | MNILELSKLNSGNENMVTILHFNDVYNVEPRDIEPVGGAARFSTAMKSFSHLDPLIIFSGDVLSPSVLSTFTHGEHMIDVLNILGVHCSVYGNHEFDFGVDRLLEFAERTKFPWLMSNVIDKETNEPLGSGNITYVLEHHGKKFGFIGLVEKEWLVTLATVDVDDVIYLDFVDVGEKLAKQLKENDQVDFVIAITHMRFPNDCHLAEKVEEIDLILGGHDHVYDIKVVNGKYIIKSGSDFQKFSKITLTLGNPVTSISIDEITVTSKYEEDPELKEILKNYEGVVAVKMDEVLAHFSVDLDGRFSSVRTMETNIGNFVCDVMLAATHADLTILNSGTIRCDRIYPKGPFKMRDLITVLPLMDPMVVLKCTGYQVWKALENGVSQYPRLDGRFPQVAGISFKFDPMKPPGERIDPADIKVSFEPLDMNQVYLVATKKYCALGRDGYEALKKCEIITDEENTPELCISVQNHFRSIKFLTGAARQRSHHRQSLFCVSRRASLRHFDEIPSFSGLMRSMSCDTIAPDIPLVRTVSIDEIEHEQCRLAPKVEGRIQILTEEFKEKLEREKSQLSILNDVIEEVSE |
| CliEnz5Nuc02 | XP_023236967 | 0 | 5´nucleotidase | MFIAAVLAVLLPGHIWT**YQLTILHTNDVHARFEEFNKYGGRCTESLSQKGECFGGVARQMTKVREIRETNDNVLFLNAGDYYQGTFMYTVHKWKIVADFMNRLGHDAMALGNHEFDDGVEGLLPFLENVKFPVLGCNIDVGKVPFLKGKILSSIERDVGGEKIGIIGYVTPETSFLARPGNVSFKTESDCLKEQAEQLNSKGIKIIIALGHSGFTRDLEIARTIPYLDVVVGGHTDTFLYTGNPPSIEEPQGKYPVVIDKEDGSKTLVVQDYTYGKYLGFLQVEFTDDGKVQSWTGNPILLDEKVEEDPLIQNALKPYVEKVEKISKEVVGKSRVLLLGERSTCRMRECNMGNMLTDAVVSEFTKMPKDGGWTSVSVALWNSGGIRSSIDERYADGNITMEDLMNVIPFAGIFLIVELKGSDLITAMEEAVENYDVTGVDPPGAFLQVSGMKIRYNLKKNPGERVEKILIKCSNCRVPKYFPLNTTSYYKVAITDFIYNKGDGFKTFMEKSTSVISTGLEDNDIIKSYLRTSSPLVAGVEDRISFQDENRCLKSTNFQPSNRKVNILLIILSVVYSYLR*** |
| Hyaluronidase | | | | |
| ID | ID reference protein | E-value | Sub-classification | Amino acid sequence |
| CliEnzHya01 | XP_023226974 | 0 | Hyaluronidase | MHSISIFSIFISIIYSAQA**DFKVYWEVPSFLCSKKYKINITQDLTSHKVLVNQGEGFNGDKIVIFYENQLGKYPYIDPTKGDVNGGLLQVADLKEHLKVSKDDITKFIPNPKFDGIGVIDWESWRPSWDFNWGKMKVYRERSIDLVKSKHPDWSSKKIEETAIKEWEDSAKEWMVKTLKLAEDMRPDAGWCYYYFPDCYNYNGKDQPSQYTCNARVREQNSRLSWLWNQSTALCPSIYTQESHIKKYNMSQRAWWIDARLRETMRLANPNTPIYPYINYVLPGTNETIPSMDFKRMLGQIASLGLDGAIIWGSSYHVLTKSQCELTATYVKDVMAPTIATVVLNTNRCSQAICKGRGHCYWPDEPFTSWKYLIDPKMPVFKPTNISCKCKGYTGRYCQIAP*** |
| Protease inhibitors | | | | |
| Ascaris-type | | | | |
| ID | ID reference protein | E-value | Sub-classification | Amino acid sequence |
| CliPInTIL01 | XP_023235660 | 3.00E-60 | TIL | MKTYIATIICIFFLCFSQDSA**QPLICGEGQELIDCITPCGPRRCSTYLENTIHPERCASILLPLCTIGCQCKDGKFLNDEGECVELINCSLDFS** |
| CliPInTIL02 | XP_023229070 | 1.00E-47 | TIL | MKAFLIIALLIIVASTVNSA**ITTPQCGENEEFDSCGTACPVDCTNYMNEPEPCTRQCVIGCACKRGFVRSATKKCIHPSEC*** |
| CliPInTIL03 | ABY26681 | 3.00E-23 | TIL | MSSKIICLTVLSLLVMNVAA**QRGFCGPNEELKGCGACDGSCRTPNVACTADCRPPSCGCIRDYVRDPSGRCIPLESCYRN*** |
| CliPInTIL04 | XP_023217168 | 1.00E-95 | TIL | MKMSNAWKIILLVCGINNIFG**KEIHTSEDNCEKNEIYMKAGCEPLCDNILKGPPFCLNNETRQGCFCKTGFIRNSSDHQQRNQCISIEECGIRVCTRPNTELNFEGRISFCTKDGIVGSIKYPLGFLSICNCKRGFVWKDYICIPEDECRKSL*** |
| CliPInTIL05 | XP_023224657 | 7.00E-95 | TIL | MLVNYGTLLLFLVLAIDGAKT**QRCGVNEVFYDARCEPYCDNALDDPCYQQVERSGCFCQTGYIRDQRTKKCIRLEQCSSRVCRQPYTELDLNGRFTICSGPRQSYTGYPYRRKPACACISEYAESREGCIPISQCRRLERN*** |
| CliPInTIL06 | XP_023217141 | 1.00E-112 | TIL | MLLHSIYSLDLSIYAYCKRVRIMWKLLKLIIVVTAVNG**VPINQLCGKNEEFRHQSCEPTCNRVHDQPCSVVEKYDGCFCKTDFIRDEKNKCIPVGECSKKMCNMPNEELNLNGDLRFCNGSGTYYTTKFKFDVIKICFCKDGYAKKHDACVPIKECT*** |
| CliPInTIL07 | XP_023238824 | 5.00E-99 | TIL | MFKVVLSCGICLIFIHWAVDG**SGITEGVCGVDEVYVKAGCESSCKNLLKEPCLGRPTHPGCICKAGLIREESTNHCIPIKECGQRICQQPNRELNLDGRFTICTGPGQAYTGHPFRPHPVCTCKRGFARSEGICIPVSECKAPQLNRNSE*** |
| CliPInTIL08 | XP_023217773 | 8.00E-35 | TIL | MSLFKFILVSVFFVDVLNIVA**QRVCTGPNEELKEACLKLNEELKPTIRNDSNICIPVKECCPEPNVRNDYNICIPVKQCCPKPNEELKPCGACDGTCTNPNPICPFICKSSSCGCIKGTVRNKYNKCIFCSEECENMQYFISYMP*** |
| CliPInTIL09 | XP_023217773 | 7.00E-35 | TIL | MSLFKFILVSVFFVDVLNIVA**QKACLKLNEELKPTVRNDYNICIPVKQCCPKPNEELKPCGACDGTCTNPNPICPFICKSSSCGCIKGTVRNKYNKCILEHAVFHILYALKTPLNADQLRVDASYMLRFMQTSFVWMHRGNCQKRL*** |
| CliPInTIL10 | XP_023217773 | 8.00E-24 | TIL | MSLFKFILVSVFFVDVLNIVA**QKACLKLNEELKPTVRNDYNICIPVKQCCPKPNEELKPCGACDGTCTNPNPDIPKNMQYFISYMP*** |
| CliPInTIL11 | XP_023217773 | 5.00E-31 | TIL | MSLFKFILVSVFFVDVLNIVA**QKACLKLNEELKPTIRNDSNICIPVKECCPEPNVRNDYNICIPVKQCCPKPNEELKPCGACDGTCTNPNPRTCSISYPICPEDTLECGPASCGCVLYAPIYADQLRVDASRELSETIIIYVSQ*** |
| CliPInTIL12 | XP_023217773 | 5.00E-37 | TIL | MSLFKFILVSVFFVDVLNIVA**QKACLKLNEELKPTIRNDSNICIPVKECCPEPNVRNDYNICIPVKQCCPKPNEELKPCGACDGTCTNPNPICPFICKSSSCGCIKGTVRNKYNKCIEECENMQYFISYMP*** |
| CliPInTIL13 | XP_023227691 | 3.00E-57 | TIL | MLLILLLVFLGNVFA**YNQECFSPFEEYSECTGNCDRYCEWSNGTIMAPRCSEDCQEGCVCQNGYVRIHKEWFAYCIPESDCKKCPDNEHFDICTHHCQKNCENKDELVPCASICIPGCICNSGYVREVDEDSPCIPAEDCNN*** |
| CliPInTIL14 | XP_023210698 | 0 | TIL | MDQHKLFVLILFLSLYQLAQS**KSKRKLGFHGPKPGPIASKNLCDIPKPPANSIDHCEDKTHQRECTYRCLEGYTFPSGTLEITYTCVFREGWTSVVDIPDCTPVCKPPCENNGECIEQKCICPAEYRGNACEYPISLCQPSFQNTTGIIQCNHDRNVSRCTFVCPQQTEPWDLPEKQYTCNLRGEWSKSFPDCIPFNGWIGEIVEEEASAFPNWFKFDYFKRTNNIVSRTKKVIKHREDVYLSQNFYSSGVCATWGQFNYKTFDGYIYSFHGPCTYVLVEECKSNSFSIHLKNDPQCLTESECVRLISVIIENKKFEVTKNEGIMMIRREYQNLTIPGRVDNLQFYQSNEFLVLESSFGFRLRWDGKETVLVTVESFLRNKTCGLCGQFIGRSGRYMLKANGEVDNDVVEFANSWKVMYDENEKCNSLQVGQHVCKYRTDTERMLFKKSRETCEKIFNHPIFSTCQKLVSDELFERACRLEFCACKIENEKCICSTLAEYLRECVRHGGQVSGDWRSITNCTIECPEGMIPAKCGRECPKTCQGTTYLCSDQTCVDSCICPEGKVLDTINNRCVLQEECPCIFEDAEYSPGGRRIQDCNECDCIKGKWLCTNRPCEARCVVSGNKHYTTFDGQRYEIMGSCSYYLVYNKEFYVLQQKTKCSDLSSNIVSDNRATCTKAIVIGYKKDVFVLKRFEIYVNNEEETIPYIGQEVLIDKLSHFIKVDMPNGLTVFWDKMNRIYIDATPDLYNRLYGLCGVLNGDISDDLFSRYGEKETNIEIFAGSWKDPTVECREIMLNVKDFCKSDEKRLQLAEKACYPIHMDTFAACHISVDPHVYYRECVEDMCSCLTGDCYCSAIANYGVACARKGKTVKWTEDIPSCASQCHLRQTFDDCTDPCEYSCTSLAMSKAVRACTKKCVQGCTCPKGNTLNVNGTCIPFQQCPCLYNGKQYHEGESIQVEGASCICLAERWECVGEKQSVVAFADAVECNDDMKIPNNSTSCGSEELKYLYCFGVATTEECMREPCRCKDTYSMDRETKKCLPPTKCTGCMFYGRKLQREATIYKHSDCEIWKCSEDKSMGVSVKDVTDEYSETVCFNNPCRVWGQENILTFKKELFKFHKSTCFYYLLKHDKYSIAYKNIRCPKSDIVCSIEVEFKVENETVHLPKDLNSTEQKREHFIVYEKGIWIVVHSDKQIILAWDRGTRVYIYVNSETLKSDTVNGLCISKKSEGTKIDAYCILVSKNRIDEDNIDITRKSTREQCRILDENIFRDCKKNLLKFSDLKEICEKEAGICSTMDFCKCHCHTISVIADICSDMGMEMNWRNSTFCPLENVDVCNYTGRISVCPEDVCESEATIKNYCKYLPPVEGTEKAHCPEGKVRVSYSNRTCISKDDCKKRVQCIVNGTAYEEDEIIPFDVATFCKNCICRDMQLQNCTECVTECKDGIQYFEVNTTALDFANNVSCGREYITKVDCRGPDILDNGKLKSDECIIENVLDCFQTGKEGKSEICYKHGFHIQCTCVEIPPGKKNETSPTTPLTTTTSTSPATPTSPATPIHVTKCNQPIGMWDNNTIKDSQIIVSSTAKGTNLTKGDKIRYNNDKPQVISGLGSSIIIKVDLGEVYFLVGMMYRGKHDPNVTESFKVFSSLNKEISTTPVPPGEDYFVDTNMENKSAYFSHSIEAGYIVFTVQHHFLQSRPDPFNFILQIELLGCKNESSSTVEVTETETEFTSVSTVPPCNSSLNITTESFEEYDTKYISVNFTTPGIYYVTTVYIKNNFCNTKYIEIYVIINLKPSDNWEENESMHIQKIFTSLQDIWIIPVDKSEVEFLYLNFLDEESCNYTKTEVTVYGCEITTPPTTLSTTLSSPSTGPTT** |
| Kunitz-type | | | | |
| ID | ID reference protein | E-value | Sub-classification | Amino acid sequence |
| CliPInKun01 | XP_023217494 | 6.00E-51 | Kunitz | MKTFLAICVSLFFCINVVLE**AKHETKALQPGKEYHQFFFHNYEKNKCEAFIYDPCYGMYNPDNRFETQQQCCEKCGGNNCS*** |
| CliPInKun02 | XP_023217492 | 2.00E-61 | Kunitz | EVVCIVILIICINTDA**TRYNIPIKCHLQPDSGCLAYLLRYYYNISTDKCELFSYGGCPGNGNNFETLQECCDECSATNLDCEQSKKYMQDMIDSLPRPSGKSIEMW*** |
| Serpins | | | | |
| ID | ID reference protein | E-value | Sub-classification | Amino acid sequence |
| CliPInSrp01 | XP_023234887 | 0 | Serpin | MNTLLFLILAGLSTVYA**ECIEENDALDPNWDNILKSFEIGNMEFGLELFRNLNSDSKNDSTNLFYSPLSIWSALSSLYIGARGQTAKELENVLGLDFTKKFFLPKLFDKFLEICSQCGDNENASFKMANRIYIDKKVELKLCEDVLKNIVKKIDFSENPKISGEEINKWVEEKTNGKIHDVIPVSAVTSETQMVVINAIYFKEHWKTQFNPEMTRNSRFYMNRDTIYRVDMMNTHGTFIYGASEEMKCQALEIPYSGDELSMLLLLPQHPYNGFDNLVKTITGSRLKNLINSMSHRELWVTIPKFKVEQEFELSNVLQKMGLRSMFNPVFTDLSGFTGKKDLTVDAVYHKSYIKVNEEGTEAAVTTSILLSRVARPGGITRFVADRPFLYLIRHVQSNVILFMGTVKSPQY*** |
| CliPInSrp02 | XP_023216488 | 0 | Serpin | MMKIFLLLFCSIFLIANA**QQEIKKLASTSNEFCFSLLKTFPINQNVFFSPANIYLSLGMLYAGARGSTAETMQSVLGYSDEGNIHLAFSNLINLLTSQSDEYRIELANALVYQESFHISPQFKEILQIYYGALVKELDFEESAEEALEEINQWVEENTNRKIPKFLDELPPDLVMILLNAVYFKGIWQKQFDPELTQDAIFYNDGVNKVTVSMMNIKDHLPYVWYPQKWLNAVELPYKGEDISMLIILPFRHDKLDEIEKNLDEESLKDIISLLKILPNKIDVSIPKFKLEDCRKLKSNLTYLGLDDIFNRNADFTGINNDYDLLVSEIFHKATIEVNEEGSEIAAVSGIELVPESLIGIVYANHPFLFFIRDLRTNMILFAGRVTRL*** |
| CliPInSrp03 | XP_023236972 | 0 | Serpin | MQLLLLLLLFAISLILPFIEG*YGRGVSSGISAFIESNNNFAFALYKDLLKEKNLVISPWSISRGLASIYLGAQNYTKREMEEVLFKNEAGVTGKEMIVSYGHLER***LLKRKAQVDLTTFNAAMIQQGSPVSETYKHRLFHYFNSILYDLDMANHGKLVRDWINILVEIKTNGLIKDILTKVPTSDTILLLLNGIHYKGEWVQKFDPELTTVSTFYNIDKVPVQAAMMLSTSNFTYRYSHLDDVHVLRLPLQGKFAMTFVMPGENGSLEEVGKTLNYKTLQRIISEEATPKMIKLSLPKFKLESKEDLAEALIDLGMHTLFSPVNADLGGIDQNKGLFLKDVIHQATVEVSEEGVEAAATTLLGVESRLGAIYIPFNRPFMFLIEDLDTGLIIFMGHLSDCTNVCAVPASDDQQKLNSDSF*** |
| CliPInSrp04 | XP_023216515 | 0 | Serpin | MWLTNQVTTFVVLFTLFPSKNPYIEG*QEQVKYTERRSHSESLVLANNKLSFKLLKTMENGKNIFFSPFSIHSALSMLNEGAKGVTSQEIREVLGYDITGVNSNTTSRDFNVLLRLVESFGPEYQLQVANVLLTQSNYPVFKEFVTKISEDFKAFVKNINFETGEDSVREINNWVNDATGGKIKSIIKEIQEDTKALILNAVYFKGSWENPFDEKNTEDFIFYNNGINPVNSPIMSR***EISKCMYTRNTEEGYHAVSLPYKGNDVEMIIILPLPKYTINDIRLSDEKMEEIILSMRNFKVFVKLPKFTIEYFRDLKEDLITLGMKQAFTNYANLSGINEDKRLFVRSVLHKAIIEVNERGSVATGTTAVIVGTRISPVLFICDHPFLFVIRHRKTRMNLFIGQINQL*** |
| Other Venom Components | | | | |
| CAP superfamily | | | | |
| ID | ID reference protein | E-value | Sub-classification | Amino acid sequence |
| CliOthCAP01 | XP_023212786 | 0 | CAP | MAKVQLLLLALFFQPFSSFA**CHYSKLSYRHTMCIYNPHACPNSQLLKSGGLTHKDKALIVRIHNRIRSSVASGWVSGLPPASNMRVMMWDNELAQIAQRWADQCTEGHDQYRNTRRFSVGQNVALQWTYSHRDLKWKNRPDWNSSINLWAKELAQFGFPRSYINPFHFDGNVGHYTQMIWGSTYTIGCGYAYYKHPYKGYTKIYVCNYGPGGNIIGGKMYEESRGGKKCTNPKLILSKQYSGLCEKRSLYKRMRTHHKSSKSTAGRRKQRKTRVRTKQLL*** |
| CliOthCAP02 | XP_023241578 | 0 | CAP | MKGTLFFDISPKLIFLLIYLCSSVLS**DCPALYKRYSHEHTFCKTKNQKCHVKKWGVSEDDREIIIDLHNKVRNNIATGKDQSGRLPAAGDMLEMEWDDELAQIAQKLADQCVFKHDCDDCRKVENFDVGQNIYTSTITAVKPPESFWVDAIRSWYSEIYRFTPDFIKPFTSDHATGHFTQMAWSTTWRVGCGYVLYEKRRDSWTQLYVCNYGPAGNIDDSEMYKVGKPCDKCPGNTCCGAHCKTRKPSSYLGLCKVLNGRGPDFDETDFGNFIFNCDFRPESSSDCNSKVEGSNKWQTRQIISDVYKTVVLNGGESSSLKFSSNIQSKNGFCLTVSFRKGPNVAGTKSDSKFDLQLERRGSAPLSFELDSEGNQWLPYSMGIPMNQPMQINLKFSVPKGSPAQYLDVNYVRARPGVCK*** |
| CliOthCAP03 | XP_023216533 | 1.00E-156 | CAP | MNLSRNMTDLAAFRMNERYVSINNNNNKLYRNQSLPANNPIKSRPFDPNAIKQQMYTRHNNYRYLHNSPLLCWSAELAEFSQVWANKIAEKGYLQYSENPSLGENIVIVDLQECPTGEEIVDKWYEERKYYDYDKPGWSKSTFHFSQMIWRSSSEIGVGVQKFKNKNCAAIVVNYKPCGNDNLPGEYKKNVLMPKPVNMKDIKKRNSCQNSF* |
| CliOthCAP04 | XP_023228306 | 0 | CAP | *MAATYVR***QCVKKPDCYKHNYTDDTQMEQNFKAVVYNKNYEVKDPILRFANIIKEWAFEIKDLPGSIVQNFQPDQSPENDWVNLFRATTYKVGCGIVTFQHDNEKFKEIYACNYAPAKLKRGEEIYKKGKTDWCTECPAEMKCQRKWFRLCAPEDDIDDEPNEILWQCDFSIGAMKQCEYVIQCSKDWKTVCGLHTCHEEITTNKPKSSLLFMTPILVKDQACLRFNYKKNHLPTSQRESTLTAIAVWNNGKNYTSLKIDEDVNTWMPYSLKIPVKNKKIQVGFVVRKVGNSEGQTISIQNLVILSGSC*** |
| CliOthCAP05 | XP_023242168 | 0 | CAP | MIFAVLFLSMTISELVVS**QSCPAIYRRLSKNHSYCMSSTCKVIAGGKVSESDKQTILKVHNELRSKLATGKETQYQKLPSAANMMEMEWDDELAAIAQAHANQCKFEHDSGDQRAVGNFSVGQNLFQSSGSLSINWNGVKMWYTSEVKYFHPEYNNPFQFQSTYGHFSQVIWAKTWKVGCGLAGYEENGVKKVLYTCNYGPGGNSKGSEVYQVGSPCSACPKNTKCSDTYPGLCKSLTSDGPQPSRPSSSDYLLYCDFSNEDPQACKDVKMTGSRSFSTQKVYTGKYVTAVFNAGEKMTINFGKFQHKDGLCAFLIGRFGPNVAGEKAGSFVSFHFAAPGLIFPDGMKETKISSSWHTIGILMQSDTEMEVSYTFEVNAGAPPQYFEFKEYGVKGGKCP*** |
| CliOthCAP06 | XP_023234678 | 0 | CAP | MYNLNSQCDKKYSDITPDHSMCQPKNEGCKRLRSGYMQKYTVLKVHNELRNNIRSYNQRFPLATNMLKMEWDDELYEIAKHHVLRCIEQPDCSKCHQTGGTHVEQNFEVLSYPNNYEIDTPAGRFRDVIKKWASEISGLPRKIIKKFLSAESPKKNWVNIFRATTYKVGCDSINYNVDNKTYKEIYVCNYTPATLTEGEEIYKRGLSCSECPDGMGCDTFYKRLCAPIKTTTSSTIDALITVPASTTAASTATAAVPTTTASVPTTTAAAPTITAPPSTTVSASAVSSTLTNINADNLTSTTITTITTFQTTFINNEILWECDFSIGTKKQCEFEMQCYKNWKTICGFEDCYQEIIIHKPKSSLLFIMPISIEDQACLIFDYKKEYLSIAQDKSRLIAVAVWNEGENYAAVIIDDDANIWTMVSLLIPVRNQEIQIGFIVRKPNNSEGQKISIRNVHVLNGSC* |
| CliOthCAP07 | XP_023230542 | 0 | CAP | MEKANENRNLYHGRKKGISKLRPPQLTTNKFGFVKESCVAEKAEFEQLEKQSKVFCKENVPKTEFKQCDLKLGDPVCIGGVKKGLLRYYGETKFADGIWCGIELDEPVGKNNGIIEGVQYFQCKENHGIFAPVSKVQLISNAELSHNAQQPVSSNFRVDNFQQVHKFSYKNLTDFRRTSSPKESLSHLTFCSDQESRIPFPNYVGTKKDSHSEQCLSFVLRRNSGFGKDNKNSSVKELKDCENLCSDTFVRNEKEESAKIMPSSLLIDKTMHSTESLTVAFPETNAKANLTHLLDKDDLNSTFTLDKPAEISETTIVKDQDALNTTFDVDLSPINDNFNKLSDPCLKLISDLNSTFTLETTTEVEKDKEEPKENECNFLKSEKKLSFEDAFDINDIDDDQPSLDESLGILTPNQMKDFSIDQQGVIVFDGDMFRIPKMASCDNIDDIPEDEVKETLSHLNDPYVQILAENRENDFESADLLYESRTLPKSTKITIDNNDEFDEVSKNSRQTSTPYGNTRTLKSEFPSSPLIGSSEFNMGIDSYDKNLKLVMKDQTKTDTTLGDDIITDNKNIERKINEIDQEEIPSCSFIHNITFSNQIDGDDVNFDSELKSLNNDKEKVSAISNENLQISIENLNMQHISDNNCNNAPLTIDSEISENNFLTNNIPICSDTNVLMKSDIEVIKVERPASTYTTGSTDTGYQEDGDFDVQSEISAAVPSPCSEIPTHSIYNSNRNMGLEDNERHQIEMSDSDFFTDSGGVGFTTESEMETDGECKMVSDFTVESNGMDTVVERKNQNEDQRSAILTTKQDDTLKEIISENVSEDLAKDVTVSQTVTSSLNSVCDSFTT |
| Insuline Growth Factor Binding Protein | | | | |
| ID | ID reference protein | E-value | Sub-classification | Amino acid sequence |
| CliOthIGF01 | XP_023217449 | 2.00E-28 | IGFBP | MLCFMILFGLIVSSYC**IPCSCDKKSCKPVTNCKFGMVKDGCNCCQVCAKGVGESCGGRFNVHGICADDLECVYSRSLTNLEKLKRGGTCIGA*** |
| CliOthIGF02 | XP_023234366 | 8.00E-29 | IGFBP | MKKFFFIICLCFAIFIDISA**LDCKECQRNQCDDKTEEECLAGLVTDLCDCCLVCGKGENEDCGGTFGMLGKCGTGLYCKTENEEDVYSDGICQKIE*** |
| CliOthIGF03 | XP_023234364 | 7.00E-67 | IGFBP | MTFKFFTFLLVSVCLYSMAVS**LSCLPCDKSACPPLSEADCPVGIVLEGGCGCCQVCGKNVGETCGGPWNIQGNCGIGLVCVKPPPPAEDLHVHEFNSIGKCQLKH*** |
| CliOthIGF04 | XP_023234740 | 1.50E-68 | IGFBP | MNLYFVFTLCMLFCMENTISA**LTCLACDQNTCEKKTEEDCPAGLTRDVCECCVICAKDIGERCGGIWNMYGKCGRNLKCVNPSNPLTTFDYANEYGICLPIRE*** |
| CliOthIGF05 | XP_023234363 | 3.00E-66 | IGFBP | MDLRLLTFLLVSVCLYSVVVS**LSCRPCDKSACAPVKEADCPVGISSDACGCCQRCAQNVGEKCGGPWKVYGKCGKGLICVKPPAPEEVNPSLYEFNSKGTCQLKN*** |
| CliOthIGF06 | XP_023217417 | 1.00E-63 | IGFBP | MLRFVILLSLIGSLYA**LSCPCWEWTEKQLKEYCPDISNCPLGLTHDSCGCCQECEKALGEVCGGPWFTSGRCGKGLRCQTDDGNEVSDEDYPGTLDDGTCVTV*** |
| CliOthIGF07 | XP_023234355 | 8.00E-71 | IGFBP | MASRLSIFIVLLSGLLDVGRT**LTCIPCEEATCINKTEEECPVGTVYNTCGCCKVCAKNVGEICDGPYKVYGQCGRGLICVKPSPPPGIDSFLHYFNIEGICQVNNEKL*** |
| CliOthIGF08 | XP_023234354 | 8.00E-48 | IGFBP | MSRIFLCIFFCAGLLLCVET**RKCLTCDKSKCDEKKESDCPAGLVLNKCNCCLVCGKALNEPCGGGYRNYGRCGRGLVCKPDSTSPDNASTCQKA*** |
| CliOthIGF09 | XP_023233099 | 0 | IGFBP | MKRTARAAAFYLLLVVGPLSA*QSTPAARLGSEVER***CSPCRCPEEPPSCPRGVAVVTDGCGCCPICGRQQGEACDRVQLCDSGRRLRCHYRRPRDPTGICQVKQGRSCLVAGRVYPDGETFKLDCRTQCTCQNGTYGCVSLCPHENIRPSGNCRNPQLVPLRSACCREWLCETSVWEHKEPDCERYSSEWSPCSVSCGAGWSSRVTNHNAECRMRKESRVCQIRPCQETTGPPAVGHHTRRNHLCRATVKSSSPVRIVDDRNCSSVKLYEPKFCGRCR***GRRCCRPRLSTTVEMTFDCSSSDREEGSPLRLTRDYMWIVKCVCDDRC** |
| CliOthIGF10 | API81348 | 9.00E-19 | IGFBP | MRTLNILLIFIVIASTNA**CDFCFNYECPPPPEDCPFGTVLDGCGCCLVCAKGEGESCGGVWDVEGICAEGLICMERSRIYR***GVKDLPGICRKLKQ** |
| La1-like peptides | | | | |
| ID | ID reference protein | E-value | Sub-classification | Amino acid sequence |
| CliOthLa101 | XP_023215406 | 8.00E-66 | SVWC | MLNTLIALLALGTLANC**YTFNSTVRMRDGKCVYGSELKEDREDWYNPDFCERLTCRIVEDKAYIIITDCGVPSSPNPNCKIEKKEGNYPDCCPRIICP*** |
| CliOthLa102 | XP_023215422 | 8.00E-69 | SVWC | MAFVTKLYTCGICVCLLVQFLYA**AILLTPQEVGPGVCIDKNGNKHELKDVWTDNDRCERHKCVMIRGIRHIKTYRCGVIDVPEGCKMIRGEGPYPQCCPDIEC*** |
| CliOthLa103 | XP_023241610 | 3.00E-78 | SVWC | MNYLILFSLMSVLVVVNS**YTARKNVPNVNGKCDINGVLVKSGDTYYEEDACEAWICSASSSPTAYKVLDDGTVQPVYNKKAQVEILGCGVATVEKNGKTCHVQTTTGIYPECCNGPEVCP*** |
| CliOthLa104 | XP_023215405 | 7.00E-67 | SVWC | MVFLTKLYTCIICVCLLVQFLHA**AILHTPQEVGPGPCIDKNGIKRKLKEIWTDNERCEKHRCVVIRGIRNIKTYKCTVIDNPEGCTIIKREGPYPRCCPDIQC*** |
| CliOthLa105 | XP_023215405 | 2.00E-48 | SVWC | MVFLTKLYTCIICVCLLVQFLHA**AILHTPQEVGPGPCIDKNGIKRKLKEIWTDNERCTVIDNPEGCTIIKREGPYPRCCPDIQC*** |
| CliOthLa106 | XP_023241612 | 2.00E-49 | SVWC | MIYIFLIFLMCVVYEVNS**YTATITVPKVNGKCEINGKLFKRGEKYMSKQKCEVWTCLKKSPRKNGKLDKKLALVNVKGCRKIWMDIDSVNDCFFKSTTGKYPDCCFGEQECGERNFAKNNDFFDIMFE*** |
| Undefined peptides | | | | |
| ID | ID reference protein | E-value | Sub-classification | Amino acid sequence |
| CliOthUnd01 | AMX81473 | 2.00E-27 | KIP/CIP | MFQVLTLSCLIFSYIYSCQG*EDEEGRLFFNFIFSDEGRK***LLRCFGTFGFSYSMKTDIRSKMEAQEKLCNCTSAAIKTT*** |
| CliOthUnd02 | XP_023228376 | 7.00E-49 | Undefined | *MFTTINIALRR***IIIVTPILFLYLMAELYMPFVEAQFFHHHFNITACIVCDEGVNYSVRNFMCCLFSSKCCGEEKFKD*** |
| CliOthUnd03 | P0DL69 | 5.00E-11 | Undefined | MNKIVALLLLTVLVLTVVPISEVES**KYCYNDDDCKSECMVVKYCQYGTCYCKGN***GK** |
| CliOthUnd04 | XP_023227575 | 3.00E-107 | Undefined | MKTSMDMTPLLMLFIFAIVLSPTNS**IGIPDIIPIDPNVIPIDPNVIPIDPNVIPLDPLKDLSKTMDMYCGLSCAEKRDFRFCLIENGLQTVLDFAKNCAQGLQFFPTTDEITEFVCKLTEASPEVFGKFLKCHSSAQKTFSITNPNVYLIIAKCLETSKTC*** |
| CliOthUnd05 | XP_023229377 | 8.00E-81 | Undefined | MRLHRGSSLLTFIFFLFILTITFS*MPHYSFRDKR***SGISDQRLAELETLINLAKQKNRGRPPIAFGVIDPLKV***GKRKRSNDVTEMDDLRELFDDPLKEKEYAENARFWDLMTDLRRYTN** |
| Other transcripts matched by LC-MS/MS | | | | |
| ID | ID reference protein | E-value | Sub-classification | Amino acid sequence |
| CliEnzAML01 | XP_023225708 | 0 | Alpha amylase | MVQMVTILLFLFLQAVLC**SYYEPNTVPGKSVFVHLFEWRWKDVADECEQFLGPFGFGGVQISPPNENGIVWEPFWNKEIKRPWFERYQPVSYKLGTRSGTESEFREMVRRCNKAGVRIYVDAVINHMTGNIGKGQGTAGSHFDPGALQYYGVPYGPSDFNNDKCHSQSGNIENYQDKHQVRDCRLSGLADLNLGKQYVRDKITEYLNYLIDIGVAGFRFDAAKHMWPSDIKALRDRLKNLNTEFFPPNTRPFVFQEVIDLGGGEAAKADEYLHIGRVTEFRYGKHLGDIIRKNYDQRLKYLKNFGEEWGMVPGGNAITFIDNHDNQRGHGAGGFGTILTFFESRMYKMAVAFMLAWPYGLPRVMSSYQWPRHIEHGKDKNDWIGPPHDDNYNIKPVIRNSDMTCGNGWVCEHRWRQIYNMVKFRNVAGFEPVDYWWDNNYHQIAFGRKGKGFLVINNDNHPVDQNFLTGLPAGTYCDVISGNLENNSCTGKKVNVGNDGRAQIFVDNNWEDPMLAIHIEAKLK*** |
| CliEnzTGa01 | XP_023224970 | 0 | Protein-glutamine gamma-glutamyltransferase | *MATRPRGSNYGRSRNMDPEKEHKLREMERLREEYLKRKREEMLREQALKSGGKTLEVDVVELYAKDNALDHNTSRYDLLNGKDAPAIFRRGQLFYMCIRFKR***NFDLSQDKIKLIFLIGPQPQVSKGTLVQLPITGNQSFTKPKSQWDIRLHHQEQAIIHLQVQIATKAIVGVWSLNIETTHPSSTVPSHFQASKNIYILFNPWCKDDAVYMEDEACRNEYVLNESGKIFVGSHKSPKGRRWIYGQFSSAALPAAMAILEASPLDYVGRANPVKVVRTVSAMINSEDDNGVLKGRWDGSYDDGTAPWIWTGSTAILEEYLRNGGNAVKYGQCWVFAGVCTTVCRALGIPCRPVTNFVSAHDTDDTLTIDKFFDPKGEKMELNDDSIWNYHVWNDCWMTRPDLPPGYGGWQAVDATPQETSDGVYQTGPASLEAVRRGEVGFSYDSPFVFSEVNADIIHWQKDDDAPIGWKKLKTNKYHVGRFILTKKVGVEDHKGDTDVENIINLYKNVEGTAEERIAIRNAATYGNFTELFEMPATGEEDIFFDMLEIDQIMIGEPFDIVLKIENLSNSVRTIHTVMSANTVYYTGINAHVVKKEIRKLVLNPRQKETLTIKVTSKEYYDKLVDYSMMKIYAMATVKETKQTWTEEDDFAVEKPKLKLEVSGRIQVGKPFDLVASFVNPLDRILQDCVFIIEGPGLSEPHRFYFRDIDPKETAVYKESFVPSKEGKRNIVVMFSSRQLIEVQGSMEVEVTP*** |
| CliOthIGI01 | XP_023222952 | 5.00E-172 | Inmunoglobulin I-set | MKNWISLATIIVFLSAMIGSSLS*RVISNKRTIGNSAHQNVFRRMKR***SAEFLKFHKKPPSSIRLLTGSNRVLECEAGASPSPMIVYWLKDGKKIDEESSTDEVINGDVESYTITMVKSRLYLDCVTPDDAGEYTCVIENAYEKKSKNVKVEIMESEEDLCVDREISSSARINLWTTRMLKTQGYDVRLICRSEGSPTPKITWLDTEENPISDSNKYKLMENGDLIIYDLKWNDMGHYTCLSENSRGSDTAVLFLYPLLPENKD*** |
| CliOthLTR01 | XP_023218207 | 0 | Vitellogenin | LINKHQTLTFVLFSQISDADLQDIVKKECRHSSDSESFLQPGHVYKYRYHSVGSIAFAGVNDRSSEIELECSVTVSAAVNCLYNLELSGCKTSAGGGPNGNLQYWVKDRDLSELTEYPVLFSLNNGKLGSIFALPQDPIYSVNIKRGIISGFSLSKSSADGKERTQRDVHGNCPRQTNSISDQKYSSHKIINRCQFPSRPNWKLSPFSLFWNTSFAQVLIRSSVDCDYDVNSGQMKLEKVSCHEKHILLPHSSKNTEVSVQANIKYHLTLESAKSALFKQRYQLNEMRATGIEMEYEKIEEPTTQPPDFLQNAQDILSDLVLFSQEEVQIRAAGLFDELLHLVRQSTDLIPFIDSVTACNFIKGVPKCNKVQKQLALQFLKDAISQCNTGPCMKGFRHLVVRDQISRMYLNLVLISLTNIPIPNPTYIEEILAICKHTEMKLCWLTLGTAIYKYNQEHSEIPQAVSDAVSYLTSYISDDCNVEDVTFPSDYTTADKNEYLLTVIKTIGNIGDTARVAHNTVVKQLYSCAAGKETSLEVSVAAIKAMYRMKPNNYIHKKLTYLMRDTTRAVGVRLAAYDVLVNLFDDDDLAKEIALLLREEKSIQVKCYIASDVQMIEQEHVGSTKTDTFADKLKEHFKAEGIYLEDIRCSPIHHSSSYRDSSFYDFHFLPKEMRGFGSKYEHKVIFETLSALPYSSTFNYTVQLFSKYYNLFETGITAKNWEEFGIWIRRQLSDSNINVGQWVITNFIEVVRKLGIKGLIPVVSNFGGEELSFVYPDQKVADNLRKVVKQFLDANIKQKPSLEIYLKIFGNELAFVTLNGIFSFLTTQYHWISNQNIGESLRKGIEYDYTRTLKIGESYHHVPTSMGLPLTWGSNAINIISPRIKLDATENVGDQTQHLELVHSNSYSVIDELILEFPTVTKIGLRGNTSTHASWNADLKFHHSEAHDFHEITSKVPEKKQKYFELYRRNQEIFGDKIEDIPIDETFIKENDGCTGKNFNEITGVKLCHYKYYPNLPDELKTSTFLFGRIESKYSLEKYDSEFKYSTLQISTPRNNEKGSFEIKLKNSAPGSKYTREYDIDLKVNSETKEYNLGVTHKDFPDFALTAYRTKIHNDDHVETGVKHYWLLQCHADKKYEMKYNVNEQTESKPIHQGHKGHHKRSAKSKEFAASTRERTLDFETPYNIYRWNSKLFTHHGVKKAEALLTYENLADGWKWPAKYLPDSMWETDKKAWLHFEGELEIEKGESDRFHRHSIGRLSSPKRSVEIVTHKMKTPENLHLQANATVIEKPSGKFLSFINGSYHRSEFNARGTWKRAHEFKLVIPRYSWDIRAEREISDSESSSSAVYTRYRMVPKTEEGKSYVGEPIQLDNFDLEEVHIKYKGHIKHKHVENQLLPQKLRETDYIRNKDQRYPGVETIIDASLKFPNENKEESVTIKGHLLRVYEDKPRSFDIFRLSHQRPVGSELSLTGVDTLANIRREIKTVTGYKHDWVKTLVMHNDAIYRNNEKKTDYNLNLLWGEKEKCEYIDLKHDFQSSFYNFDSTILLECKPRTKAYNLEININTNSPEWDMLNSKTRQVFYLERHLEGWETMNFSHPAINIDAEGKIGMDFEGPFYDKKYHIKSKTDVLPSYEITYSSKLTSTPYMELNIEVPEDGFKNIKYRTDIARDGSGFSIVGTHVHTDKPEEVKENLVFALKLLSPFIISLKGYEDLKFSIEVAEKLHPRLGLLADKIEAIVKDRNHPINVLIKSLKKQSKKQEEFYRNAFEKLKENRKEIRELLYPFIEPTVEMIRNIRREADERTKQAQDNYESFTVAANAFKNIDYQGYANKAVTQIPQESIEAIKAYITSRENNIVKYQYTGPHPFYWQNLFQIPQPWEGTYRSPVFYLLPHLHKEILFKRRYIVSPIYKTAMIFGTSHMYTFDGKMYEFPDFPGSDCTYMLAHDVRESTFSVLLSEQKLHILFPEVTVTLDRDNKIYLNNSRQESGVPIETPNGKVFVTREGGIVSINSIGLTVLCDSQRSFCIFILDPLYHSGTIGLLGNADGEAYNDFSLPDGKHVETSAELARGYEVSNRKRCRNIQEKQKPEFTKDTQTRCSSSFPHLSTACAAYLGIEEDLFLEACRWDVSQGKDACISSDAFAGYCTVRGMKSTPCSSKPSWYLKTVNSAKKLEVILVIEEYHKMFASGLKGLDSLFSAIHDEFKKNGYESIIFSVIGYGGKGNHWDPHIYTPDSNIWHSKAELISHLENSLKFEGEHKGNTLEAIKYATTILPFDMQASRIMLLFTDHDTNTYLCGLKLQYLEDLLSKYSLTLYTFSDFESVDKGKKVFGLKSDGKVLHQGKKETYMDYPETEAAKLSADTEGSILLKRYVEANEPRQFFKTAAEQFYAKVEEESSKCRKCIWKLNNWGDIQNECSVADECKDK* |
| CliOthSCO01 | XP_023233098 | 1.00E-114 | Sco-spondin | *ITSSPSTAFTPSTPSSTTIFPERRGCERQKLSQLLYNVSSTANHDTTQYTYGIGVHFYSSEMNDNHHINIYINYARKIALIENAEDKDLLLALTYSIDNRTWTIMSKVIYLKEKYIFSDLSYQANYFTFVFLPQNNVNEIQNEIFFCSDTDCPKLNTTSCQESCHEKNLTCNGTMCVDKTNCPCIENGHIYSPKTHITINCTNQVCINGSFQSFDISCSSKNITCKEPEVKKFNRETCNCTCLCEKPNHGCITKDPEVQCIREVGICDNITDCVDGSDESCCPVSLGNETTCNFHDEQCTFVPSCRVRR***NFIIGFNKQIRSCQICDGILFRERGKQRFSIRYQFHPFSSAISVEINEYRFIMRTEFEVDVRNSETMDKYTITLSRSDSLKVVENEYQFKVVANAVVFKSLKYGFKVIWRENNMVDILPEKCLLGRTEGLCGYYVNYVNGTLIKEYRTQNGSCGVNSTEFCDDWEAFTGSCNGITVNWEKEYSSIKKGNKFCKELRNDIGEACNIEAIDFSPESYNAIEESCGYAIFSNCSDTHTDRECRCAAIRYLLSETQCDQDDGKLLGSNGCETCEKGFSWKPCSFDYTCDDFHLKKPQKGVPGCFCPKTEVKIKEECLPPKKCRNCECSIFGDPNYRTFDGRDFRFQGSCPGPYVLVQAKSSSKLPFFKILGIIEKCGPNTNFTCTTGIVIEYNKTRLEVYANKTVFVNQTEFTSYCEDVDCPINGMTLRNATQLNEFQINLHESEVDVSYVLRNAGRHQLRSLSIKFSLPMYSKTTEGLCGTCNRNDSDDFALRDGTVTDDIETFAFDWIEETDKSSCRIYSTQNCTWNTNITKCSYENKNCSFVDDKHAYRRYYQSCVEDLKCSDVIFDSQICKYKLLYAEDCCKYGVSTEDWLYDEGCDIIECKTNQTYRCVDICKTKCPGERDERCEHGKVIEMICACPDGYVESNNECIKEEDCENCLLKRKIDLPEGSINVDGCKNQKSIINLGNCEGGCNTTSKYQYESNGVTYTKNCSCCIPYRYKQVAIKLYCDKNDTTVNHFIKEPAECRCEQCNLQNK*** |
| CliOthSFU01 | XP_023230592 | 0 | Somatomedin | MDLWKLIVFASVLTGICRG**RTVSYEELVSLRADCDPIDKCNTTKENLEVPSRSCQCDDSCAFYGDCCLDAPNRQAMERMKTRNICLKKNNFQGYFAVSNCKRNWRDLETRRKCEIPNAEDPYTLIPVMSQRSKITYRNRYCASCNDDNRDLEFWKIGVTCRQLGANLSYDFPNDLVYNSYLKTWGVFLPNQTFLRCKLVPVPPENTENLRSCYSNTISTCSTNWKDESIKEKCESYTAIILARHRIKYRNVHCAVCNGVNVTDLNCLYVSSRELARTFVKIPGTPNIVCLFSVGNCSCENKVYDAAFRKCRELTCGLPFLELKNGKCVHKS*** |
