# Supplementary material for: Dissecting Toxicity: The Venom Gland Transcriptome and the Venom Proteome of the Highly Venomous Scorpion Centruroides limpidus (Karsch, 1879)
Source: Toxins (Basel). 2019 Apr 30;11(5):247. doi: 10.3390/toxins11050247 (PMC6563264; doi:10.3390/toxins11050247)
Supplement: Supplementary file 1 [file toxins-11-00247-s001.zip › toxins-494793-supplemenraty materials/toxins-494793-Supple Table S2.docx]

| **Supplementary Table S2.** The 192 venom-related transcripts obtained from the *C. limpidus* venom gland and their reference proteins. ID from UniProt or GenBank. | | | |
| --- | --- | --- | --- |
| Transcript ID | Reference protein | | |
|  | Annotation | ID | Source/function |
| α-NaScTx | | | |
| CliNaTAlp01 | alpha-like toxin CsEv5 | XP_023235403 | Genome/not tested |
| CliNaTAlp02 | alpha-like toxin CsEv5 | XP_023235403 | Genome/not tested |
| CliNaTAlp03 | alpha-like toxin CsEv5 | XP_023210703 | Genome/not tested |
| CliNaTAlp04 | alpha-toxin Cn12 | XP_023230636 | Genome/not tested |
| CliNaTAlp05 | alpha-toxin Cn12-like | XP_023242001 | Genome/not tested |
| CliNaTAlp06 | alpha-toxin Cn12-like | XP_023242001 | Genome/not tested |
| CliNaTAlp07 | alpha-toxin CsE5 | XP_023242920 | Genome/not tested |
| CliNaTAlp08 | alpha-toxin CsE5 | XP_023242920 | Genome/not tested |
| CliNaTAlp09 | alpha-toxin CsE5 | XP_023242920 | Genome/not tested |
| CliNaTAlp10 | alpha-toxin CsE5-like | XP_023228500 | Genome/not tested |
| CliNaTAlp11 | alpha-toxin CvIV4-like | XP_023238168 | Genome/not tested |
| CliNaTAlp12 | alpha-toxin CvIV4-like | XP_023238168 | Genome/not tested |
| CliNaTAlp13 | alpha-toxin CvIV4-like | XP_023228501 | Genome/not tested |
| CliNaTAlp14 | alpha-toxin Acra5 isoform X1 | XP_023216724 | Genome/not tested |
| CliNaTAlp15 | Alpha toxin CvIV4 | F8UWP3 | Venom/Induces pain in mammals |
| CliNaTAlp16 | Uncharacterized protein | A0A0U1TZ19 | cDNA library/not tested |
| β-NaScTx | | | |
| CliNatBet01 | beta-neurotoxin Css9 | XP_023241641 | Genome/not tested |
| CliNatBet02 | beta-neurotoxin Css9 | XP_023241641 | Genome/not tested |
| CliNatBet03 | beta-neurotoxin Css9 | XP_023241641 | Genome/not tested |
| CliNatBet04 | beta-neurotoxin Css9-like | XP_023235256 | Genome/not tested |
| CliNatBet05 | beta-neurotoxin Css9-like | XP_023235256 | Genome/not tested |
| CliNatBet06 | beta-neurotoxin Css9-like | XP_023235257 | Genome/not tested |
| CliNatBet07 | beta-neurotoxin Css9-like isoform X1 | XP_023235252 | Genome/not tested |
| CliNatBet08 | beta-toxin CeII8 | XP_023242923 | Genome/not tested |
| CliNatBet09 | beta-toxin CeII8-like | XP_023237823 | Genome/not tested |
| CliNatBet10 | beta-toxin CeII8-like | XP_023237823 | Genome/not tested |
| CliNatBet11 | beta-toxin CeII8-like | XP_023237823 | Genome/not tested |
| CliNatBet12 | beta-toxin Cn5-like | XP_023229318 | Genome/not tested |
| CliNatBet13 | beta-toxin Im-2-like | XP_023223307 | Genome/not tested |
| CliNatBet14 | ikitoxin-like | XP_023209741 | Genome/not tested |
| CliNatBet15 | ikitoxin-like | XP_023209741 | Genome/not tested |
| CliNatBet16 | ikitoxin-like | XP_023209741 | Genome/not tested |
| CliNatBet17 | ikitoxin-like | XP_023243062 | Genome/not tested |
| CliNatBet18 | beta-neurotoxin RjAa1 | XP_023240702 | Genome/not tested |
| CliNatBet19 | beta-neurotoxin RjAa12f | XP_023211611 | Genome/not tested |
| CliNatBet20 | beta-neurotoxin RjAa12f | XP_023211611 | Genome/not tested |
| CliNatBet21 | beta-neurotoxin RjAa12f | XP_023211611 | Genome/not tested |
| CliNatBet22 | toxin Acra I-2-like | XP_023209523 | Genome/not tested |
| CliNatBet23 | toxin Acra I-2-like | XP_023209523 | Genome/not tested |
| CliNatBet24 | toxin Acra I-2-like | XP_023209523 | Genome/not tested |
| CliNatBet25 | toxin Cn11-like | XP_023240701 | Genome/not tested |
| CliNatBet26 | toxin CngtIII-like | XP_023209923 | Genome/not tested |
| CliNatBet27 | toxin Tpa8-like | XP_023213214 | Genome/not tested |
| CliNatBet28 | CsE1x | Q95WD3 | cDNA library/not tested |
| CliNatBet29 | CsEI | P01491 | Venom/Toxic for chicks |
| CliNatBet30 | CsEv1d | P01492 | Venom/Toxic for chicks |
| CliNatBet31 | Cll2b | P59899 | Venom and cDNA/Active on Na^+^ and Ca^2+^ channels of vertebrates (mammals and chicks). |
| CliNatBet32 | Cll2b | P59899 | Venom and cDNA/Active on Na^+^ and Ca^2+^ channels of vertebrates (mammals and chicks). |
| CliNatBet33 | Cll5b | Q7Z1K7 | cDNA library/not tested |
| CliNatBet34 | Cll6 | Q7Z1K5 | cDNA library/not tested |
| CliNatBet35 | Cll7 | P59865 | cDNA library/not tested |
| CliNatBet36 | Cl13 | C0HK69 | Venom/Inhibits Na^+^ channels Nav1.4/SCN4A, Nav1.5/SCN5A and Nav1.6/SCN8A. Weakly inhibits Na^+^ channel Nav1.2/SCN2A. Lethal to mice. |
| CliNatBet37 | Cll9 | Q8WRY4 | cDNA library/not tested |
| CliNatBet38 | Toxin Cn11 | P58296 | Venom/Not toxic for mammals but affect insects and crustaceans. |
| CliNatBet39 | Toxin Cn11 | P58296 | Venom/Not toxic for mammals but affect insects and crustaceans. |
| CliNatBet40 | Toxin Cn11 | P58296 | Venom/Not toxic for mammals but affect insects and crustaceans. |
| CliNatBet41 | hypothetical protein | A0A0U1SEW9 | cDNA library/not tested |
| CliNatBet42 | altitoxin | P0C1B5 | Venom/Toxic for mammals. |
| CliNatBet43 | Lipolysis-activating peptide 1-alpha chain | D9U299 | cDNA library/not tested |
|  |  |  |  |
| KScTx | | | |
| CliKtxAlp01 | potassium channel blocker pMeKTx10-2b | A0A088DAY7 | cDNA library/not tested |
| CliKtxAlp02 | potassium channel blocker pMeKTx21-1 | A0A088D9V0 | cDNA library/not tested |
| CliKtxAlp03 | Cobatoxin-1 | O46028 | Venom/Blocker of Kv1.1/KCNA1, Kv1.2/KCNA2, Kv1.3/KCNA3, shaker channel, affects K_Ca_ channels. |
| CliKtxAlp04 | Cobatoxin-1 | O46028 | Venom/Blocker of Kv1.1/KCNA1, Kv1.2/KCNA2, Kv1.3/KCNA3, shaker channel, affects K_Ca_ channels. |
| CliKtxAlp05 | Cobatoxin-1 | O46028 | Venom/Blocker of K^+^ channels Kv1.1/KCNA1, Kv1.2/KCNA2, Kv1.3/KCNA3, shaker channel, affects K_Ca_ channels. |
| CliKtxAlp06 | Slotoxin | P0C182 | Venom/Blocker of K^+^ channels dSlo, Kv1.1/KCNA1, Kv11.1/KCNH2/ERG1, Kir2.2/KCNJ12, Shaker-IR, and voltage-independent/Ca^2+^-activated SK1/KCNN1, SK2/KCNN2 and SK3/KCNN3 |
| CliKtxAlp07 | Noxiustoxin-2 | Q9TXD1 | Venom/Paralyzing activity in crickets, not toxic for mammals and crustaceans |
| CliKtxAlp08 | Noxiustoxin-2 | Q9TXD1 | Venom/Paralyzing activity in crickets, not toxic for mammals and crustaceans |
| CliKtxAlp09 | Toxin Ce5 | P0C165 | Venom/Weak blocker of Kv1.3/KCNA3 channels |
| CliKtxAlp10 | CllTx1 | P45629 | Venom/Inhibitor of K_v_ channels. Inhibits transient potassium current *in vitro.* |
| CliKtxAlp11 | toxin KTx8-like | XP_023238900 | Genome/not tested |
| CliKtxAlp12 | BmKK4 | Q95NJ8 | Venom/Inhibits potassium current from rat hippocampal neurons in a concentration-depending way. |
| CliKtxAlp13 | Neurotoxin Ts-kappa | P56219 | Venom/Blocks KCa2.2/KCNN2, KCa2.3/KCNN3. Blocker of ERG1/Kv11.1/KCNH2. Moderately inhibits Kv1.1/KCNA1 and Nav1.7/SCN9A. Inhibits with low potency Kv11.1/KCNH2/ERG1 and Kv1.2/KCNA2 channels. |
| CliKtxAlp14 | Potassium channel toxin alpha-KTx 4.5 | Q5G8B6 | Venom/Inhibits with low potency Kv1.1/KCNA1, Kv1.2/KCNA2, Kv1.3/KCNA3 and Kv11.1/KCNH2/ERG1. |
| CliKtxAlp15 | Potassium channel toxin alpha-KTx 4.5 | Q5G8B6 | Venom/Inhibits with low potency Kv1.1/KCNA1, Kv1.2/KCNA2, Kv1.3/KCNA3 and Kv11.1/KCNH2/ERG1. |
| CliKtxAlp16 | Potassium channel toxin alpha-KTx 4.5 | Q5G8B6 | Venom/Inhibits with low potency Kv1.1/KCNA1, Kv1.2/KCNA2, Kv1.3/KCNA3 and Kv11.1/KCNH2/ERG1. |
| CliKtxBet01 | potassium channel toxin TdiKIK isoform X2 | XP_023220228 | Genome/not tested |
| CliKtxBet02 | scorpine-like peptide Tco 41.46-2 precursor | XP_023220230 | Genome/not tested |
| CliKtxGam01 | CllErg1 | Q86QV0 | Venom/not tested |
| CliKtxGam02 | CnErg1 | Q86QT3 | Venom/Has activity on ERG K^+^ channel from rats and humans, does not occlude the channel pore. |
| CliKtxGam03 | potassium channel toxin gamma-KTx 1.1-like | XP_023241648 | Genome/not tested |
| CliKtxDel01 | Delta-KTx 3.1 precursor | P0DJ50 | cDNA library/Affects Kv1.3 and inhibits trypsin, but not chymotrypsin or elastase. |
| CliKtxDel02 | isoinhibitor K-like | XP_023217495 | Genome/not tested |
| CliKtxDel03 | hemolymph trypsin inhibitor B-like isoform X2 | XP_015905918 | Genome/not tested |
| CliKtxLam01 | phi-buthitoxin-Hj1a | F1CIZ6 | cDNA library/not tested |
| CliKtxLam02 | potassium channel blocker pMeKTx30-1 | A0A088DAF5 | cDNA library/not tested |
| Host Defense Peptides | | | |
| CliHDPDef01 | defensin-1 | Q6GU94 | Hemolymph/No hemolytic or antimicrobial activity. |
| CliHDPDef02 | defensin-1 precursor | AIX87626 | Venom gland transcriptome/not tested |
| CliHDPDef03 | defensin-1 precursor | AIX87626 | Venom gland transcriptome/not tested |
| CliHDPDef04 | defensin-1 precursor | AIX87626 | Venom gland transcriptome/not tested |
| CliHDPDef05 | defensin-1 precursor | AIX87626 | Venom gland transcriptome/not tested |
| CliHDPDef06 | defensin-1 precursor | AIX87626 | Venom gland transcriptome/not tested |
| CliHDPND201 | venom toxin meuTx20 | A0A146CJE0 | Venom gland transcriptome/not tested |
| CliHDPND401 | ToAP2 peptide | A0A1D3IXJ5 | Venom/*In vitro* antimicrobial activity against *Mycobacterium massiliense*. |
| CliHDPND402 | TsAP2 | S6D3A7 | Venom/Antibacterial, antifungal, anticancer and hemolytic activities. |
| CliHDPAni01 | anionic peptide | XP_023227050 | Genome/not tested |
| Metalloproteases | | | |
| CliEnzMtp01 | A disintegrin and metalloproteinase with thrombospondin motifs | XP_023221573 | Genome/not tested |
| CliEnzMtp02 | A disintegrin and metalloproteinase with thrombospondin motifs | XP_023210991 | Genome/not tested |
| CliEnzMtp03 | A disintegrin and metalloproteinase with thrombospondin motifs | XP_023217439 | Genome/not tested |
| CliEnzMtp04 | A disintegrin and metalloproteinase with thrombospondin motifs | XP_023216256 | Genome/not tested |
| CliEnzMtp05 | zinc metalloproteinase-disintegrin-like atrolysin-A | XP_023227092 | Genome/not tested |
| CliEnzMtp06 | uncharacterized protein LOC111618883 | XP_023216275 | Genome/not tested |
| CliEnzMtp07 | venom metalloproteinase 2-like | XP_023243712 | Genome/not tested |
| CliEnzMtp08 | venom metalloproteinase 3-like isoform X1 | XP_023234770 | Genome/not tested |
| CliEnzMtp09 | uncharacterized protein LOC111634066 | XP_023234515 | Genome/not tested |
| CliEnzMtp10 | venom metalloproteinase antarease TserMP_A-like | XP_023213460 | Genome/not tested |
| CliEnzMtp11 | venom metalloproteinase antarease TserMP_A-like isoform X3 | XP_023216482 | Genome/not tested |
| CliEnzMtp12 | venom metalloproteinase antarease-like TpachMP_A | XP_023234498 | Genome/not tested |
| CliEnzMtp13 | venom metalloproteinase antarease-like TpachMP_A | XP_023234503 | Genome/not tested |
| CliEnzMtp14 | venom metalloproteinase antarease-like TserMP_B, partial | XP_023234510 | Genome/not tested |
| CliEnzMtp15 | venom metalloproteinase antarease-like TserMP_B, partial | XP_023234510 | Genome/not tested |
| CliEnzMtp16 | venom metalloproteinase antarease-like TserMP_B, partial | XP_023234510 | Genome/not tested |
| CliEnzMtp17 | venom metalloproteinase antarease-like TtrivMP_A | XP_023228637 | Genome/not tested |
| CliEnzMtp18 | venom metalloproteinase antarease-like TtrivMP_A | XP_023240574 | Genome/not tested |
| CliEnzMtp19 | venom metalloproteinase antarease-like TtrivMP_A | XP_023234508 | Genome/not tested |
| CliEnzMtp20 | venom metalloproteinase antarease-like TtrivMP_A isoform X1 | XP_023234500 | Genome/not tested |
| CliEnzMtp21 | venom metalloproteinase antarease-like TtrivMP_A, partial | XP_023214049 | Genome/not tested |
| CliEnzMtp22 | venom metalloproteinase antarease-like TtrivMP_A, partial | XP_023214049 | Genome/not tested |
| CliEnzMtp23 | astacin-like metalloprotease toxin 3 | XP_023229533 | Genome/not tested |
| CliEnzMtp24 | angiotensin-converting enzyme-like isoform X2 | XP_023209358 | Genome/not tested |
| Serine proteases | | | |
| CliEnzSeP01 | aclotting factor B-like isoform X2 | XP_023238707 | Genome/not tested |
| CliEnzSeP02 | chymotrypsin-like protease precursor | XP_023239597 | Genome/not tested |
| CliEnzSeP03 | plasminogen-like | XP_023233218 | Genome/not tested |
| CliEnzSeP04 | proclotting enzyme-like precursor | XP_023227283 | Genome/not tested |
| CliEnzSeP05 | serine protease 48-like | XP_023210062 | Genome/not tested |
| CliEnzSeP06 | serine proteinase stubble-like | XP_023209481 | Genome/not tested |
| CliEnzSeP07 | transmembrane serine protease 9-like precursor | XP_023237211 | Genome/not tested |
| CliEnzSeP08 | transmembrane serine protease 9-like precursor | XP_023230910 | Genome/not tested |
| CliEnzSeP09 | uncharacterized protein LOC111628841 | XP_023228456 | Genome/not tested |
| CliEnzSeP10 | uncharacterized protein LOC111628841 | XP_023228456 | Genome/not tested |
| CliEnzSeP11 | uncharacterized protein LOC111630971 | XP_023230909 | Genome/not tested |
| CliEnzSeP12 | uncharacterized protein LOC111638400 | XP_023239874 | Genome/not tested |
| CliEnzSeP13 | venom protease-like | XP_023243443 | Genome/not tested |
| CliEnzSeP14 | transmembrane serine protease precursor 11D-like | XP_023239572 | Genome/not tested |
| Phospholipases | | | |
| CliEnzPA201 | phospholipase A2-like | XP_023236869 | Genome/not tested |
| CliEnzPA202 | phospholipase A2-like isoform X1 | XP_023234362 | Genome/not tested |
| CliEnzPA203 | phospholipase A2-like isoform X1 | XP_023234380 | Genome/not tested |
| CliEnzPA204 | phospholipase A2, membrane associated-like | XP_023231606 | Genome/not tested |
| CliEnzPA205 | uncharacterized protein LOC111640561 | XP_023242353 | Genome/not tested |
| CliEnzPA206 | uncharacterized protein LOC111640561 | XP_023242353 | Genome/not tested |
| CliEnzPLD01 | phospholipase D-like precursor | XP_023237656 | Genome/not tested |
| 5´Nucleotidases | | | |
| CliEnz5nc01 | 5´nucleotidase-like | XP_023233208 |  |
| CliEnz5nc02 | 5NUC-like protein | XP_023236967 | Genome/not tested |
| CliEnz5nc03 | uncharacterized protein LOC106478895 | XP_013794928 | Genome/not tested |
| CliEnzHya01 | Hyaluronidase 1-like | XP_023226974 | Genome/not tested |
| Ascaris-type protease inhibitors | | | |
| CliPInTIL01 | venom peptide BmKAPI-like | XP_023235660 | Genome/not tested |
| CliPInTIL02 | venom peptide SjAPI-2-like | XP_023229070 | Genome/not tested |
| CliPInTIL03 | VP5.1 | A0A0U1SJ92 | Venom gland transcriptome/not tested |
| CliPInTIL04 | uncharacterized protein LOC111619629 | XP_023217168 | Genome/not tested |
| CliPInTIL05 | uncharacterized protein LOC111625674 | XP_023224657 | Genome/not tested |
| CliPInTIL06 | inducible metalloproteinase inhibitor protein-like isoform X3 | XP_023217141 | Genome/not tested |
| CliPInTIL07 | uncharacterized protein LOC111637548 | XP_023238824 | Genome/not tested |
| CliPInTIL08 | keratin-associated protein 5-1-like isoform X2 | XP_023217773 | Genome/not tested |
| CliPInTIL09 | keratin-associated protein 5-1-like isoform X2 | XP_023217773 | Genome/not tested |
| CliPInTIL10 | keratin-associated protein 5-1-like isoform X2 | XP_023217773 | Genome/not tested |
| CliPInTIL11 | keratin-associated protein 5-1-like isoform X2 | XP_023217773 | Genome/not tested |
| CliPInTIL12 | keratin-associated protein 5-1-like isoform X2 | XP_023217773 | Genome/not tested |
| CliPInTIL13 | allergen Api m 6-like | XP_023227691 | Genome/not tested |
| CliPInTIL14 | mucin-2-like, partial | XP_023210698 | Genome/not tested |
| Kunitz- type protease inhibitors | | | |
| CliPInKun01 | tissue factor pathway inhibitor-like precursor | XP_023217494 | Genome/not tested |
| CliPInKun02 | kunitz-type serine protease inhibitor Vur-KIn-like | XP_023217492 | Genome/not tested |
| Serpins | | | |
| CliPInSrp01 | leukocyte elastase inhibitor-like | XP_023234887 | Genome/not tested |
| CliPInSrp02 | uncharacterized protein LOC111619073 | XP_023216488 | Genome/not tested |
| CliPInSrp03 | serpin B6-like | XP_023236972 | Genome/not tested |
| CliPInSrp04 | serpin B3-like | XP_023216515 | Genome/not tested |
| CAP | | | |
| CliOthCAP01 | Venom allergen 5-like | XP_023212786 | Genome/ not tested |
| CliOthCAP02 | Venom allergen 5-like | XP_023241578 | Genome/ not tested |
| CliOthCAP03 | Golgi-associated plant pathogenesis-related protein 1-like isoform X1 | XP_023216533 | Genome/ not tested |
| CliOthCAP04 | CRISP/Allergen/PR-1-like | XP_023228306 | Genome/ not tested |
| CliOthCAP05 | CRISP/Allergen/PR-1-like | XP_023242168 | Genome/ not tested |
| CliOthCAP06 | uncharacterized protein LOC111634195 | XP_023234678 | Genome/ not tested |
| CliOthCAP07 | restin homolog isoform X3 | XP_023230542 | Genome/ not tested |
| IGFBP | | | |
| CliOthIGF01 | venom protein 302-like | XP_023217449 | Genome/not tested |
| CliOthIGF02 | venom protein 302-like | XP_023234366 | Genome/not tested |
| CliOthIGF03 | venom protein 302-like | XP_023234364 | Genome/not tested |
| CliOthIGF04 | venom protein 302-like | XP_023234740 | Genome/not tested |
| CliOthIGF05 | venom protein 302-like | XP_023234363 | Genome/not tested |
| CliOthIGF06 | venom protein 302-like | XP_023217417 | Genome/not tested |
| CliOthIGF07 | venom protein 302-like | XP_023234355 | Genome/not tested |
| CliOthIGF08 | insulin-like growth factor-binding protein-related protein 1 | XP_023234354 | Genome/not tested |
| CliOthIGF09 | connective tissue growth factor-like | XP_023233099 | Genome/not tested |
| CliOthIGF10 | venom toxin | A0A1L4BJ69 | Venom gland transcriptome/not tested |
| La1-like peptides | | | |
| CliOthLa101 | venom protein 30.1-like | XP_023215406 | Genome/not tested |
| CliOthLa102 | toxin-like protein 14 isoform X1 | XP_023215422 | Genome/not tested |
| CliOthLa103 | toxin-like protein 14 | XP_023241610 | Genome/not tested |
| CliOthLa104 | toxin-like protein 14 | XP_023215405 | Genome/not tested |
| CliOthLa105 | toxin-like protein 14 | XP_023215405 | Genome/not tested |
| CliOthLa106 | uncharacterized protein LOC111639894 | XP_023241612 | Genome/not tested |
| Undefined | | | |
| CliOthUnd01 | venom peptide meuPep26 | A0A146CJ24 | Venom gland transcriptome/not tested |
| CliOthUnd02 | uncharacterized protein LOC111628764 | XP_023228376 | Genome/not tested |
| CliOthUnd03 | Orphan peptide CllNtx | P0DL69 | Venom/Not toxic to mammals or insects. No antimicrobial activity. |
| CliOthUnd04 | uncharacterized protein LOC111628085 | XP_023227575 | Genome/not tested |
| CliOthUnd05 | uncharacterized protein LOC111629712 | XP_023229377 | Genome/not tested |
