# Supplementary material for: Dissecting Toxicity: The Venom Gland Transcriptome and the Venom Proteome of the Highly Venomous Scorpion Centruroides limpidus (Karsch, 1879)
Source: Toxins (Basel). 2019 Apr 30;11(5):247. doi: 10.3390/toxins11050247 (PMC6563264; doi:10.3390/toxins11050247)
Supplement: Supplementary file 1 [file toxins-11-00247-s001.zip › toxins-494793-supplemenraty materials/toxins-494793-Supple Table S3.docx]

| Supplementary Table S3. Mass fingerprint results. The 395 masses identified in the *C. limpidus* venom by LC-MS, sorted by C18-RT-HPLC Retention Times (RT). | |
| --- | --- |
| RT (min) | **Molecular weight (Da)** |
| 20-40 | 2569.08, 2609.1, 2747.16, 2779.32, 2826.66, 3423.85, 4241.03, 4479.15, 4497.15, 4758.88, 4759.9, 4831.96, 6172.87, 6463.85, 7137.92, 7317.12, 7434.15, 7539.4, 7560.4, 7576.36, 7701.48, 7773.54, 7846.55 |
| 40-60 | 949.6, 991.5, 1002.64, 1406.82, 1690.05, 1764.04, 1794.06, 3420.29, 3737.04, 3765.68, 4104.8, 4224.1, 6173.88, 6573.96, 6946.15, 6988.01, 7048.98, 7226.19, 7240.2, 7338.1,7351.14, 7384.3, 7470.24, 7882.56, 7944.54, 8355.66 |
| 60-80 | 801.34, 818.37, 879.48, 891.52, 907.58, 942.54, 1032.64, 1077.52, 1154.64, 1501.68, 1518.7, 1523.66, 1530.8, 1536.88, 1564.9, 1602.88, 1816.04, 1968.84, 1975.89, 2014.94, 2036.91, 2052.2, 2075.28, 2691.16, 2779.32, 2801.11, 3402.26, 3418.28, 3434.35, 3456.23, 3458.28, 3626.7, 3730.67, 3783.85, 3788.67, 3878.75, 3971.52, 3983.76, 3985.75, 4002.72, 4009.44, 4016.73, 4021.69, 4023.69, 4030.56, 4046.88, 4081.56, 4104.8, 4140.76, 4189.87, 4208.1, 4285.11, 4303.96, 4338.99, 4354.01, 4363.15, 4382.05, 4390.98, 4433.1, 4497.15, 4556.22, 4577.1, 4688.3, 4742.9, 4758.88, 4771.35, 4830.96, 4831.96, 4865.16, 4915.16, 4951.91, 5071.2, 6002.65, 6461.89, 6857.08, 6963.05, 6972.95, 7015.18, 7073.07, 7173.05, 7357.23, 7855.46, 8038.62, 8085.45, 8284.58, 8355.66, 9032.65, 9121.98 |
| 80-100 | 903.46, 907.58, 914.46, 942.54, 949.6, 955.42, 984.54, 993.38, 1002.64, 1024.62, 1032.64, 1218.72, 1235.74, 1240.7, 1242.75, 1256.66, 1261.68, 1279.8, 1412.88, 1434.86, 1489.81, 1498.82, 1518.7, 1530.8, 1536.88, 1541.86, 1544.86, 1558.84, 1690.05, 1811.04, 1816.04, 1818.04, 2014.94, 2028.99, 2190.06, 2198.07, 2422.48, 2483.43, 2511.52, 2548.04, 2569.08, 2747.16, 2826.66, 3294.72, 3382.39, 3420.29, 3855.52, 3865.6, 3896.56, 4189.87, 4199, 4224.1, 4479.15, 5322.9, 5338.88, 5651.68, 6068.7, 6074.04, 6087.96, 6259.71, 6385.74, 6462.85, 6480.85, 6482.88, 6499.85, 6514.79, 6536.82, 6793.95, 6795.95, 6802.96, 6815.96, 6817.92, 6837.03, 6844.92, 6887.16, 6891, 6943.93, 6945.11, 6988.01, 7048.98, 7073.07, 7111.14, 7154.95, 7173.05, 7227.22, 7232.22, 7240.2, 7257.18, 7267.4, 7272.16, 7273.21, 7279.14, 7296.17, 7315.16, 7316.15, 7317.12, 7352.15, 7357.23, 7373.16, 7383.24, 7384.3, 7432.1, 7433.28, 7436.35, 7462.08, 7507.43, 7518.31, 7539.4, 7551.48, 7582.38, 7603.68, 7613.35, 7615.38, 7649.53, 7655.55, 7665.2, 7674.3, 7689.48, 7701.48, 7711.04, 7743.55, 7745.46, 7753.53, 7755.48, 7768.38, 7846.55, 7874.53, 7882.56, 7944.54, 8513.35, 10154.34, 10155.3, 10902.1 |
| 100-120 | 1289.6, 1412.88, 1414.9, 1434.86, 1604.9, 2231.36, 2747.16, 2877.25, 2922.32, 3018.68, 3612.36, 3651.65, 3737.04, 3834.52, 3928.8, 3948.73, 3952.8, 3969.51, 3980.85, 3991.5, 4006.75, 4014.7, 4197.95, 4241.03, 4257.05, 4281.95, 4286.16, 4297.96, 4397.4, 4516.1, 4538.25, 4743.95, 4759.9, 4775.35, 4791.35, 4815.95, 4865.16, 4879.18, 5071.2, 5321.94, 5322.9, 5420.87, 5536.68, 6068.68, 6172.87, 6173.88, 6446.82, 6536.82, 6589.95, 6655.92, 6780.95, 6784.96, 6791.96, 6815.96, 6844.92, 6871.00, 6895.92, 6943.93, 7043.1, 7050.05, 7137.92, 7184.17, 7336.1, 7338.1, 7349.16, 7351.14, 7379.16, 7399.26, 7413.15, 7469.05, 7470.24, 7476.51, 7479.05, 7486.82, 7570.4, 7583.76, 7598.35, 7613.35, 7615.38, 7640.34, 7649.53, 7655.55, 7665.2, 7674.3, 7689.48, 7701.48, 7711.04, 7743.55, 7745.46, 7753.53, 7755.48, 7767.3, 7768.38, 7773.54, 7841.3, 7846.55, 7874.53, 7882.56, 7889.6, 7904.52, 7944.54, 7960.56, 7973.52, 8354.64, 8513.35, 10154.34, 10155.3, 10902.1, |
| 120-140 | 1077.52, 1086.58, 1098.36, 1786.02, 2043.2, 2052.2, 2645.08, 2816.36, 2819.25, 2821.24, 2862.63, 2865.6, 2880.76, 3432.32, 3434.35, 3765.68, 3985.75, 4030.56, 4031.88, 4033.8, 4052.56, 4073.52, 4171.15, 4297.96, 4338.99, 4397.4, 4743.95, 5338.92, 5354.88, 5436.84, 6259.71, 6385.74, 6461.89, 6478.33, 6589.95, 6793.95, 6795.95, 6837.06, 6853.08, 6870.08, 6871, 6891, 6895.92, 6901.09, 6905.1, 6927.95, 6945.11, 6959.9, 6972, 6972.95, 7067.01, 7077.12, 7112.49, 7208.16, 7257.18, 7269.12, 7339.12, 7414.15, 7419.24, 7430.22, 7434.15, 7459.14, 7567.44, 7576.36, 7577.43, 7735.56, 7753.53, 7768.38, 7849.56, 7973.52, 8201.8, 8354.64, 8513.35, 9126.06, 9367.12, 9605.47, 9763.46, 9824.43, 10142.34, 10154.34, 10155.3, 10902.1, 16038.81, 16052.84 |
| 140-160 | 2609.1, 2746.14, 2859.24, 3728.7, 4001.75, 4118.95, 4173.15, 4177.96, 4178, 4199, 4257.05, 6791.96, 6963.05, 7108.12, 7229.22, 7300.14, 7318.08, 7334.1, 7414.15, 7459.14, 7462.08, 7485.33, 7488.1, 7495.38, 7501.76, 7503.24, 7507.43, 7510.15, 7550.4, 7551.48, 7560.4, 7567.44, 7576.36, 7577.43, 7665.2, 7674.3, 7735.56, 7753.53, 7768.38, 7849.56, 7904.52, 7973.52, 8137.28, 8143.74, 8158.8, 8354.64, 8513.35, 8514.95, 9125.83, 9126.06, 9367.12, 9605.47, 9763.46, 9824.43, 10142.34, 10154.34, 10155.3, 10861.13, 10902.1, 11584.23, 16038.81, 16052.84 |
| 160-180 | 4433.1, 6463.85, 6478.33, 6709.85, 7082.1, 7154.95, 7208.16, 7263.15, 7269.12, 7419.24, 7546.38, 7561.41, 7577.43, 7592.76, 7687.48, 7706.46, 7743.55, 10902.1, 12947.2 |
| 180-200 | 6877.95, 7553.36, 7566.42, 7613.35, 7640.34, 7690.4, 10142.34, 10861.13 |
| 200-220 | 6780.95, 7566.42, 12832.3, 12931.28, 12947.2, 13781.04, 14098.08, 14193.68, 14275.84, 14357.28, 14482.44, 16038.81, 16052.84, 16068.86, 17130.7, 18969.9, 19017.9, 19069.8 |
| 220-240 | 1133.54, 1136.6, 1154.64, 1218.72  2016.38 |
