# Supplementary material for: Dissecting Toxicity: The Venom Gland Transcriptome and the Venom Proteome of the Highly Venomous Scorpion Centruroides limpidus (Karsch, 1879)
Source: Toxins (Basel). 2019 Apr 30;11(5):247. doi: 10.3390/toxins11050247 (PMC6563264; doi:10.3390/toxins11050247)
Supplement: Supplementary file 1 [file toxins-11-00247-s001.zip › toxins-494793-supplemenraty materials/toxins-494793-Supple Table S4.docx]

| **Supplementary Table S4.** LC-MS/MS results. The 52 transcripts that were identified in venom by LC-MS/MS. The parameters generated by the Proteome Discoverer software are shown for each identified fragment. With lower-case letters are shown amino acids with posttranslational modifications identified by the software | | | | | | | | | |  |
| --- | --- | --- | --- | --- | --- | --- | --- | --- | --- | --- |
| **ID** | **Score** | **Coverage** | **No of peptides** | **MW (kDa)** | **Peptide sequence** | **Xcorr** | **Z** | **MH+(Da)** | **m/z** | |
| **NaScTx** | | | | | | | | | |  |
| CliNaTxAlp03 | 2661.99 | 75.41 | 6 | 6.5 | ISCVISGK | 2.88 | 2 | 863.46391 | 432.2356 | |
|  |  |  |  |  | FCDTECK | 2.08 | 2 | 959.35771 | 480.18256 | |
|  |  |  |  |  | KDGYPVDNK | 3.64 | 2 | 1035.51006 | 518.25867 | |
|  |  |  |  |  | KASSGYCYSLSCYCEGLPENAK | 4.43 | 3 | 2544.07999 | 848.69818 | |
|  |  |  |  |  | ISCVISGKFCDTECK | 0.29 | 2 | 1803.84294 | 902.42511 | |
|  |  |  |  |  | ASSGYCYSLSCYCEGLPENAK | 3.72 | 2 | 2415.98613 | 1208.4967 | |
| CliNaTxAlp04 | 1584.14 | 98.51 | 6 | 7.2 | AGSDYGYCYWWTCYCQHVAEGTVLWGDSGTGPCMS | 1.2 | 3 | 4081.60434 | 1361.20789 | |
|  |  |  |  |  | DGYPIASNGCKFGCSGLGENNPTCNHVCEK | 0.3 | 3 | 3342.43467 | 1114.81641 | |
|  |  |  |  |  | DGYPIASNGCK | 2.56 | 2 | 1182.5064 | 591.75684 | |
|  |  |  |  |  | RDGYPIASNGCK | 4.46 | 2 | 1338.60862 | 669.80768 | |
|  |  |  |  |  | FGCSGLGENNPTCNHVCEK | 5.36 | 3 | 2179.89219 | 727.30225 | |
|  |  |  |  |  | RDGYPIASNGCKFGCSGLGENNPTCNHVCEK | 0.39 | 3 | 3499.49961 | 1167.17139 | |
| CliNaTxAlp07 | 267.51 | 50 | 2 | 7.2 | KDGYPVNSGNCK | 2.78 | 2 | 1338.61174 | 669.80768 | |
|  |  |  |  |  | NVSCYCQGLPDNEPIMKSGR | 0.23 | 2 | 2343.99858 | 1172.50293 | |
| CliNaTxAlp08 | 1649.32 | 90.48 | 6 | 7.1 | TSGKCKGK | 0.36 | 2 | 865.47222 | 433.23975 | |
|  |  |  |  |  | YECLRDDYCK | 0.73 | 3 | 1421.57938 | 474.53131 | |
|  |  |  |  |  | KKDGYIVDSGNCK | 3.88 | 3 | 1483.71943 | 495.24484 | |
|  |  |  |  |  | DGYIVDSGNCK | 2.05 | 2 | 1227.52873 | 614.26801 | |
|  |  |  |  |  | KADDGYCFLGK | 4.66 | 2 | 1273.58684 | 637.29706 | |
|  |  |  |  |  | FSCYCYGLPDNSPIK | 4.94 | 2 | 1820.79534 | 910.90106 | |
| CliNaTxAlp11 | 36.91 | 95.16 | 4 | 6.9 | CWCYGLPDSVTTEENCKGKR | 0.41 | 2 | 2460.03008 | 1230.51831 | |
|  |  |  |  |  | NDYPVDENGCKIACQENAPCAAACKFNK | 1.88 | 4 | 3246.36001 | 812.3457 | |
|  |  |  |  |  | IACQENAPCAAACKFNKAEGGVCPWSYR | 0.2 | 3 | 3218.42942 | 1073.48132 | |
|  |  |  |  |  | AEGGVCPWSYR | 3.46 | 2 | 1281.56658 | 641.28693 | |
| CliNaTxAlp13 | 49.21 | 74.6 | 5 | 7.3 | NCKFECWK | 0.48 | 2 | 1172.50395 | 586.7547 | |
|  |  |  |  |  | NAYCDKLCK | 0.51 | 2 | 1172.50395 | 586.75323 | |
|  |  |  |  |  | AESGYCYGWNLSCYCEGLPDDEPIKTSDR | 4.9 | 3 | 3442.42527 | 1148.14661 | |
|  |  |  |  |  | RAESGYCYGWNLSCYCEGLPDDEPIK | 6.99 | 3 | 3139.32126 | 1047.11194 | |
|  |  |  |  |  | AESGYCYGWNLSCYCEGLPDDEPIK | 0.65 | 3 | 2983.21982 | 995.07813 | |
| CliNaTxAlp15 | 764.2 | 50.91 | 5 | 6.2 | DGYPVTSDKYSLPCWHSK | 1.74 | 3 | 2139.97849 | 713.99768 | |
|  |  |  |  |  | ICILLK | 2.34 | 2 | 759.47875 | 380.24301 | |
|  |  |  |  |  | YSLPCWHSK | 2.17 | 2 | 1177.54643 | 589.27533 | |
|  |  |  |  |  | VCNRICILLK | 0.3 | 2 | 1289.7105 | 645.35889 | |
|  |  |  |  |  | YSLPCWHSKVCNR | 0.3 | 2 | 1706.75737 | 853.88318 | |
| CliNaTxBet01 | 182.65 | 89.23 | 4 | 7.6 | LACYCEGLPNWAK | 4.36 | 2 | 1581.7199 | 791.36212 | |
|  |  |  |  |  | DGYPMDHKGCKISCVINNQYCETECVK | 1.33 | 4 | 3324.30117 | 831.83075 | |
|  |  |  |  |  | KGYCYFWK | 0.97 | 2 | 1151.53362 | 576.27124 | |
|  |  |  |  |  | VWDRATNKCR | 0.22 | 2 | 1305.64336 | 653.32532 | |
| CliNaTxBet05 | 6759.82 | 96.83 | 11 | 7.1 | DGYAMR | 1.5 | 2 | 712.30718 | 356.65723 | |
|  |  |  |  |  | GKSGYCYFLKQSCYCAQLPDDK | 0.23 | 2 | 2689.12041 | 1345.06384 | |
|  |  |  |  |  | DGYAMRSDGCTIPCLFDNSFCNRK | 0.47 | 3 | 2884.25333 | 962.08929 | |
|  |  |  |  |  | KCIEQK | 1.13 | 2 | 805.42302 | 403.21497 | |
|  |  |  |  |  | VYPSATNK | 1.72 | 2 | 879.45445 | 440.23087 | |
|  |  |  |  |  | SGYCYFLK | 3.25 | 2 | 1037.47502 | 519.24115 | |
|  |  |  |  |  | VYPSATNKCR | 0.28 | 2 | 1195.56572 | 598.28625 | |
|  |  |  |  |  | SDGCTIPCLFDNSFCNRK | 4.78 | 3 | 2190.93619 | 730.9837 | |
|  |  |  |  |  | QSCYCAQLPDDK | 3.94 | 2 | 1484.6115 | 742.81012 | |
|  |  |  |  |  | QSCYCAQLPDDKVYPSATNK | 5.33 | 3 | 2345.05002 | 782.35486 | |
|  |  |  |  |  | SDGCTIPCLFDNSFCNR | 5.02 | 2 | 2062.84165 | 1031.92615 | |
| CliNaTxBet06 | 24.74 | 67.69 | 5 | 7.5 | LACYCEGLPDWAK | 1.66 | 3 | 1582.72904 | 528.24786 | |
|  |  |  |  |  | LACYCEGLPDWAKVWER | 2.89 | 2 | 2152.95732 | 1076.98254 | |
|  |  |  |  |  | IPCVINNRFCEIECVNALK | 0.53 | 3 | 2351.09873 | 784.37109 | |
|  |  |  |  |  | IPCVINNR | 0.2 | 2 | 985.52385 | 493.26556 | |
|  |  |  |  |  | KGYCYFWK | 0.97 | 2 | 1151.53362 | 576.27124 | |
| CliNaTxBet08 | 130.12 | 65.08 | 4 | 7.4 | YNCWKNTYCDKLCK | 1.35 | 2 | 1953.81035 | 977.40698 | |
|  |  |  |  |  | KGESGYCWWNLSCWCIGLPDNTNTK | 6.98 | 3 | 3046.33298 | 1016.11584 | |
|  |  |  |  |  | GESGYCWWNLSCWCIGLPDNTNTK | 0.33 | 3 | 2918.23258 | 973.41693 | |
|  |  |  |  |  | LCKEKK | 0.34 | 2 | 805.45781 | 403.23254 | |
| CliNaTxBet10 | 206.48 | 67.24 | 5 | 6.6 | IKNDYPVR | 1.86 | 2 | 1005.51641 | 503.26184 | |
|  |  |  |  |  | CWCYGLPDNIDTEK | 3.67 | 2 | 1770.74211 | 885.87469 | |
|  |  |  |  |  | NDYPVRNGCRIK | 1.33 | 3 | 1492.72331 | 498.2442 | |
|  |  |  |  |  | IKNDYPVRNGCR | 0.69 | 3 | 1492.72234 | 498.24509 | |
|  |  |  |  |  | AEGGVCPWSYR | 3.46 | 2 | 1281.56658 | 641.28693 | |
| CliNaTxBet12 | 70.37 | 28 | 3 | 8.4 | RFACWCAGLPK | 0.47 | 2 | 1365.65239 | 683.32983 | |
|  |  |  |  |  | RFACWCAGLPKSTPTWPLPNK | 3.12 | 3 | 2488.18924 | 830.06793 | |
|  |  |  |  |  | FACWCAGLPKSTPTWPLPNK | 4.09 | 2 | 2332.09062 | 1166.54895 | |
| CliNaTxBet13 | 1537.03 | 87.1 | 5 | 6.8 | VWCVINSK | 2.49 | 2 | 1005.51848 | 503.26263 | |
|  |  |  |  |  | KDGYPMDNK | 3.67 | 2 | 1067.48039 | 534.2442 | |
|  |  |  |  |  | VWCVINSKSCDNTCK | 0.42 | 2 | 1872.79253 | 936.8999 | |
|  |  |  |  |  | VWDRATNKC | 0.52 | 2 | 1149.55315 | 575.28021 | |
|  |  |  |  |  | SGYCYTLGLACWCEGLPSDAK | 6.28 | 2 | 2408.03472 | 1204.521 | |
| CliNaTxBet19 | 23.44 | 30.36 | 2 | 6.3 | ISCVINDK | 2.71 | 2 | 948.48131 | 474.74429 | |
|  |  |  |  |  | KDGYPMDSK | 1.44 | 2 | 1040.47136 | 520.73932 | |
| CliNaTxBet20 | 211.16 | 56.14 | 3 | 6.3 | SGSCYSLK | 1.12 | 2 | 901.40672 | 451.207 | |
|  |  |  |  |  | KDGYPVDNK | 3.64 | 2 | 1035.51006 | 518.25867 | |
|  |  |  |  |  | ISCVINDTFCDNECK | 3.46 | 2 | 1874.76689 | 937.88708 | |
| CliNaTxBet21 | 18240.1 | 87.3 | 4 | 6.8 | KEGYPMNSK | 3.88 | 2 | 1053.50176 | 527.25452 | |
|  |  |  |  |  | GLPEDVEVWDSATNK | 5.91 | 2 | 1659.78716 | 830.39557 | |
|  |  |  |  |  | IGCVIGNTFCDTECK | 5.56 | 2 | 1773.75871 | 887.383 | |
|  |  |  |  |  | ASSGSCWTFGLACWCK | 5.22 | 2 | 1877.77641 | 939.39063 | |
| CliNaTxBet26 | 353.71 | 73.85 | 5 | 7.5 | NCNMLCKHRNR | 0.38 | 2 | 1502.67522 | 751.84149 | |
|  |  |  |  |  | ECWCEGMPESTPTWPIPGK | 4.66 | 2 | 2261.9666 | 1131.48645 | |
|  |  |  |  |  | CTNKTGERNCNMLCK | 0.3 | 2 | 1903.78899 | 952.39813 | |
|  |  |  |  |  | ECWCEGMPESTPTWPIPGKK | 3.97 | 3 | 2390.05918 | 797.35876 | |
|  |  |  |  |  | GSRGYCYNRECWCEGMPESTPTWPIPGK | 0.28 | 3 | 3375.42331 | 1125.81262 | |
| CliNaTxBet29 | 684.03 | 72.06 | 5 | 7.9 | LGENKYCNR | 0.25 | 2 | 1154.53349 | 577.77039 | |
|  |  |  |  |  | GGNYGYCYGFGCYCEGLADSTQTWPLPNK | 2.53 | 3 | 3335.38987 | 1112.46802 | |
|  |  |  |  |  | HRGGNYGYCYGFGCYCEGLADSTQTWPLPNK | 0.82 | 3 | 3630.48923 | 1210.83362 | |
|  |  |  |  |  | KDGYLVDK | 3.17 | 2 | 937.50841 | 469.25839 | |
|  |  |  |  |  | GGNYGYCYGFGCYCEGLADSTQTWPLPNKR | 6.84 | 3 | 3491.48838 | 1164.50098 | |
| CliNaTxBet30 | 2320.84 | 92.31 | 6 | 7.3 | KDGYLVSR | 3.31 | 2 | 937.50939 | 469.25839 | |
|  |  |  |  |  | YCHIECTSMNHR | 5.53 | 3 | 1607.64823 | 536.55426 | |
|  |  |  |  |  | LSCAPMIGDR | 3.28 | 2 | 1119.52629 | 560.26678 | |
|  |  |  |  |  | GMPENAEVYPLPNK | 3.91 | 2 | 1558.75456 | 779.88092 | |
|  |  |  |  |  | GDEGYCYLLGCYCR | 3.61 | 2 | 1785.70073 | 893.354 | |
|  |  |  |  |  | GDEGYCYLLGCYCRGMPENAEVYPLPNKSC | 0.59 | 3 | 3589.50656 | 1197.17798 | |
| CliNaTxBet31 | 10506.63 | 95.45 | 8 | 7.6 | QQYGKGAGGYCYAFGCWCTHLYEQAVVWPLPKK | 2.87 | 5 | 3922.8288 | 785.37158 | |
|  |  |  |  |  | QQYGKGAGGYCYAFGCWCTHLYEQAVVWPLPK | 4.55 | 4 | 3794.73183 | 949.43842 | |
|  |  |  |  |  | YECYK | 0.32 | 2 | 762.31273 | 381.66 | |
|  |  |  |  |  | ECKQQYGK | 0.39 | 2 | 1040.47136 | 520.73932 | |
|  |  |  |  |  | LGDNDYCLR | 3.98 | 2 | 1125.49883 | 563.25238 | |
|  |  |  |  |  | KEGYLVNHSTGCK | 5.51 | 2 | 1492.71819 | 746.86243 | |
|  |  |  |  |  | GAGGYCYAFGCWCTHLYEQAVVWPLPK | 7.61 | 3 | 3190.44101 | 1064.15186 | |
|  |  |  |  |  | GAGGYCYAFGCWCTHLYEQAVVWPLPKK | 4.87 | 4 | 3318.53244 | 830.38843 | |
| CliNaTxBet33 | 12260.75 | 78.79 | 5 | 7.3 | AKNQGGSYGYCYSFACWCEGLPDSTPTYPLPNKSCS | 0.44 | 3 | 4124.67807 | 1375.5636 | |
|  |  |  |  |  | AKNQGGSYGYCYSFACWCEGLPDSTPTYPLPNK | 4.23 | 3 | 3789.62802 | 1263.88086 | |
|  |  |  |  |  | KEGYLVNK | 3.42 | 2 | 950.53203 | 475.76801 | |
|  |  |  |  |  | YGCFWLGK | 3.48 | 2 | 1030.48027 | 515.7439 | |
|  |  |  |  |  | NQGGSYGYCYSFACWCEGLPDSTPTYPLPNK | 6.85 | 3 | 3589.51279 | 1197.1803 | |
| CliNaTxBet37 | 1541.78 | 71.64 | 6 | 7.9 | YYQSAHGYcYAFAcWcTHLYEQAVVWPLPNKR | 5.54 | 4 | 4038.83291 | 1010.46368 | |
|  |  |  |  |  | YYQSAHGYcYAFAcWcTHLYEQAVVWPLPNK | 4.33 | 3 | 3882.73276 | 1294.91577 | |
|  |  |  |  |  | LRYYQSAHGYcYAFAcWcTHLYEQAVVWPLPNKR | 4.09 | 5 | 4308.01588 | 862.409 | |
|  |  |  |  |  | LRYYQSAHGYcYAFAcWcTHLYEQAVVWPLPNK | 1.47 | 5 | 4151.91785 | 831.18939 | |
|  |  |  |  |  | LGDNDYcVR | 2.1 | 2 | 1111.48296 | 556.24512 | |
|  |  |  |  |  | YTcAKLGDNDYcVREcR | 0.62 | 3 | 2179.89017 | 727.30157 | |
| CliNaTxBet38 | 27.03 | 85 | 5 | 6.8 | LACYCEAVPDNVK | 2.68 | 2 | 1538.69316 | 769.85022 | |
|  |  |  |  |  | ARDGYPVDEK | 1.52 | 2 | 1149.55315 | 575.28021 | |
|  |  |  |  |  | WCNSACHYR | 2.51 | 3 | 1253.49195 | 418.50217 | |
|  |  |  |  |  | LSCVINDKWCNSACHYRGAK | 0.77 | 2 | 2440.09404 | 1220.55078 | |
|  |  |  |  |  | WCNSACHYRGAKYGYCYSGK | 0.24 | 2 | 2488.06133 | 1244.5343 | |
| CliNaTxBet39 | 548.78 | 80.95 | 5 | 7 | LSCVINNK | 2.87 | 2 | 947.49529 | 474.25128 | |
|  |  |  |  |  | WCNSACHSR | 3.02 | 3 | 1177.4607 | 393.15842 | |
|  |  |  |  |  | ARDGYPVDEK | 1.52 | 2 | 1149.55315 | 575.28021 | |
|  |  |  |  |  | GCKLSCVINNKWCNSACHSR | 0.38 | 2 | 2451.06241 | 1226.03174 | |
|  |  |  |  |  | YGYCYTGGLACYCESVPDDVK | 6.72 | 2 | 2477.00908 | 1239.00842 | |
| CliNaTxBet40 | 67.15 | 95.24 | 6 | 7 | LWTSETNTC | 1.19 | 2 | 1111.48357 | 556.24481 | |
|  |  |  |  |  | ECYCEGVPDNVK | 2.85 | 2 | 1469.60149 | 735.30438 | |
|  |  |  |  |  | YGYCYVAGR | 3.04 | 2 | 1108.48552 | 554.74664 | |
|  |  |  |  |  | GCKLSCFMNHEPCRK | 0.47 | 2 | 1923.85215 | 962.42645 | |
|  |  |  |  |  | ARDGYPVDEK | 1.52 | 2 | 1149.55315 | 575.28021 | |
|  |  |  |  |  | LSCFMNHEPCRKACVSR | 0.74 | 2 | 2152.95708 | 1076.98218 | |
| **KScTx** | | | | | | | | | |  |
| CliKTxAlp15 | 392.84 | 91.89 | 6 | 4 | CSSSSECWPACK | 2.78 | 2 | 1458.54131 | 729.77429 | |
|  |  |  |  |  | AVGTFQGKCMNGGCK | 0.24 | 2 | 1632.66643 | 816.83685 | |
|  |  |  |  |  | VFIDKK | 2.33 | 2 | 749.45409 | 375.23068 | |
|  |  |  |  |  | AVGTFQGK | 1.94 | 2 | 807.43431 | 404.22095 | |
|  |  |  |  |  | KAVGTFQGK | 2.9 | 2 | 935.53191 | 468.26892 | |
|  |  |  |  |  | KAVGTFQGKCMNGGCK | 0.48 | 2 | 1758.82598 | 879.91663 | |
| CliKTxBet01 | 794.59 | 53.73 | 4 | 7.5 | EIMNKIKK | 0.54 | 2 | 1003.58159 | 502.29443 | |
|  |  |  |  |  | GAWDKLTSK | 0.21 | 2 | 1005.51933 | 503.26331 | |
|  |  |  |  |  | FCEDHCAAK | 0.39 | 2 | 1137.44316 | 569.22522 | |
|  |  |  |  |  | SEYACPVIEK | 2.97 | 2 | 1195.56389 | 598.28601 | |
| CliKTxBet02 | 2234.29 | 31.34 | 3 | 7.6 | LLSLVVPEGQLR | 3.78 | 2 | 1323.79802 | 662.40265 | |
|  |  |  |  |  | KILQMVVHK | 3.93 | 2 | 1095.66924 | 548.33826 | |
|  |  |  |  |  | ILQMVVHK | 2.64 | 2 | 967.57414 | 484.29086 | |
| **HDP** | | | | | | | | | |  |
| CliHDPND201 | 65.69 | 80.43 | 4 | 5.3 | MWKSKLAK | 0.98 | 2 | 1007.55107 | 504.27914 | |
|  |  |  |  |  | EMIKDYANR | 2.36 | 2 | 1139.55046 | 570.27838 | |
|  |  |  |  |  | FGGFLKKMWK | 0.86 | 2 | 1241.6884 | 621.34747 | |
|  |  |  |  |  | VLEGPQEEAPPAE | 2.83 | 2 | 1365.65227 | 683.33051 | |
| CliHDPND401 | 910.64 | 75 | 4 | 5.8 | LLPSVFK | 1.79 | 2 | 803.502 | 402.25464 | |
|  |  |  |  |  | LFQRKK | 0.24 | 2 | 819.52526 | 410.26627 | |
|  |  |  |  |  | RDLEDLYDPYQR | 0.25 | 3 | 1582.73325 | 528.24927 | |
|  |  |  |  |  | FLGSLFSLGSK | 4.1 | 2 | 1155.64067 | 578.32397 | |
| **Enzymes** | | | | | | | | | |  |
| CliEnzHya01 | 1654.91 | 69.63 | 23 | 44.3 | IVIFYENQLGK | 3.9 | 2 | 1323.72832 | 662.36768 | |
|  |  |  |  |  | GHCYWPDEPFTSWK | 4.76 | 2 | 1809.76628 | 905.38678 | |
|  |  |  |  |  | AWWIDAR | 1.73 | 2 | 917.46269 | 459.23495 | |
|  |  |  |  |  | DVMAPTIATVVLNTNR | 5.25 | 2 | 1714.91533 | 857.9613 | |
|  |  |  |  |  | YPYIDPTKGDVNGGLLQVADLKEHLK | 4.24 | 4 | 2883.52089 | 721.63568 | |
|  |  |  |  |  | YPYIDPTKGDVNGGLLQVADLK | 7.95 | 3 | 2377.23099 | 793.08405 | |
|  |  |  |  |  | LAEDMRPDAGWCYYYFPDCYNYNGK | 7.49 | 3 | 3169.28098 | 1057.1001 | |
|  |  |  |  |  | MPVFKPTNISCKCKGYTGR | 0.85 | 2 | 2244.07256 | 1122.54016 | |
|  |  |  |  |  | VLVNQGEGFNGDKIVIFYENQLGK | 5.12 | 3 | 2682.37937 | 894.7984 | |
|  |  |  |  |  | RMLGQIASLGLDGAIIWGSSYHVLTKSQCELTATYVK | 1.41 | 4 | 4084.0375 | 1021.76483 | |
|  |  |  |  |  | VSKDDITKFIPNPK | 0.3 | 2 | 1601.89043 | 801.44885 | |
|  |  |  |  |  | EWMVKTLKLAEDMRPDAGWCYYYFPDCYNYNGK | 1.04 | 4 | 4217.78774 | 1055.20239 | |
|  |  |  |  |  | VLVNQGEGFNGDKIVIFYENQLGKYPYIDPTK | 2.18 | 4 | 3658.86464 | 915.47162 | |
|  |  |  |  |  | SQCELTATYVKDVMAPTIATVVLNTNR | 5.26 | 3 | 2995.5226 | 999.18109 | |
|  |  |  |  |  | INITQDLTSHKVLVNQGEGFNGDK | 0.25 | 3 | 2627.30912 | 876.44122 | |
|  |  |  |  |  | KIEETAIK | 0.21 | 2 | 931.55907 | 466.28317 | |
|  |  |  |  |  | RMLGQIASLGLDGAIIWGSSYHVLTK | 0.27 | 2 | 2803.47051 | 1402.22107 | |
|  |  |  |  |  | INITQDLTSHK | 0.22 | 2 | 1269.70452 | 635.3559 | |
|  |  |  |  |  | VYWEVPSFLCSK | 4.15 | 2 | 1514.73381 | 757.87109 | |
|  |  |  |  |  | MPVFKPTNISCKCK | 0.7 | 2 | 1710.84551 | 855.92603 | |
|  |  |  |  |  | MLGQIASLGLDGAIIWGSSYHVLTK | 7.27 | 3 | 2630.40451 | 877.47235 | |
|  |  |  |  |  | FDGIGVIDWESWRPSWDFNWGK | 5.68 | 3 | 2697.25083 | 899.75665 | |
|  |  |  |  |  | LSWLWNQSTALCPSIYTQESHIK | 7.61 | 3 | 2762.35709 | 921.45825 | |
| CliEnzSep14 | 176.72 | 17.27 | 2 | 31.6 | FANPGEFPWmVFIR | 4.42 | 2 | 1726.84245 | 863.92487 | |
|  |  |  |  |  | LTDELNcSGFLISPSYVLTAAHcmIRPLEEMWAK | 4.06 | 4 | 3968.90883 | 992.98267 | |
| CliEnzMtp15 | 49.7 | 38.78 | 9 | 44 | LFRNKEK | 0.54 | 2 | 935.52989 | 468.26859 | |
|  |  |  |  |  | CPASDGFIMGDISGNNRNKFSTCSLENIK | 0.79 | 3 | 3234.49839 | 1078.83862 | |
|  |  |  |  |  | EKNAALYINEEGLLEIKGMLTSK | 0.31 | 4 | 2582.28531 | 646.32666 | |
|  |  |  |  |  | QYDSFANRSDTIMFITRR | 0.32 | 3 | 2237.12033 | 746.37848 | |
|  |  |  |  |  | GMLTSKLRIEPYESQDTLR | 0.31 | 3 | 2237.12088 | 746.37848 | |
|  |  |  |  |  | VELIGVRTYTDENEPAFVKESLFSGTDTFEK | 0.2 | 3 | 3521.70188 | 1174.57214 | |
|  |  |  |  |  | AFGNDMELK | 0.25 | 2 | 1040.47136 | 520.73932 | |
|  |  |  |  |  | YYGQIIENILGLAFVGGACDPCKK | 7.84 | 3 | 2686.33683 | 896.11713 | |
|  |  |  |  |  | YYGQIIENILGLAFVGGACDPCK | 2.9 | 2 | 2558.24265 | 1279.62573 | |
| CliEnzMtp19 | 67.55 | 67.4 | 8 | 25.3 | MGILHDDNTLSLNYYGMAYLGSVCSSRYK | 1.09 | 3 | 3329.51035 | 1110.5083 | |
|  |  |  |  |  | VLGITSFTEETEPDFIEK | 4.72 | 2 | 2055.01543 | 1028.01147 | |
|  |  |  |  |  | DDECIVVEYLVLTESNVTK | 0.71 | 3 | 2226.10416 | 742.70715 | |
|  |  |  |  |  | HFSDHIERAAIIAHESGHLLGSQHDGEEPK | 0.93 | 4 | 3318.53261 | 830.38843 | |
|  |  |  |  |  | MYYLEPATGLAADADIIMLLVDRK | 4.15 | 3 | 2714.37925 | 905.4646 | |
|  |  |  |  |  | MYYLEPATGLAADADIIMLLVDR | 3.38 | 3 | 2586.28434 | 862.7663 | |
|  |  |  |  |  | NTFSSCSK | 0.35 | 2 | 931.39592 | 466.2016 | |
|  |  |  |  |  | SLTEYISVTYTAVQNIIDTLELGIK | 1.03 | 2 | 2784.49541 | 1392.75134 | |
| CliEnzMtp20 | 185.01 | 50.84 | 11 | 26.2 | VGVAQDDSDYNER | 0.45 | 2 | 1469.60161 | 735.30444 | |
|  |  |  |  |  | LLGIQTFK | 1.75 | 2 | 919.55901 | 460.28314 | |
|  |  |  |  |  | NCPFNDGYIMGSDDHKVNK | 1.07 | 3 | 2212.90299 | 738.30585 | |
|  |  |  |  |  | NCPFNDGYIMGSDDHK | 0.2 | 2 | 1886.75578 | 943.88153 | |
|  |  |  |  |  | HLDPVDLVVNMGK | 4.16 | 2 | 1436.7564 | 718.88184 | |
|  |  |  |  |  | NCPFNDGYIMGSDDHKVNKFK | 0.32 | 2 | 2488.0623 | 1244.53479 | |
|  |  |  |  |  | QADIIMLIITR | 3.83 | 2 | 1286.75029 | 643.87878 | |
|  |  |  |  |  | NDTGLAKQADIIMLIITRK | 0.58 | 3 | 2115.16001 | 705.72559 | |
|  |  |  |  |  | QADIIMLIITRK | 0.6 | 2 | 1414.8654 | 707.93732 | |
|  |  |  |  |  | CIVIEYLCVTESNFTKRFNTDK | 0.2 | 3 | 2737.32785 | 913.11414 | |
|  |  |  |  |  | ALTEYVTLLFTGVQNLLETLNLGIK | 3.79 | 3 | 2763.55716 | 921.85724 | |
| CliEnzMtp21 | 203.33 | 48.01 | 11 | 36 | IVEVINDGK | 0.68 | 2 | 987.52971 | 494.26849 | |
|  |  |  |  |  | DGTVKFTAMGLAYLASVCKK | 5.52 | 3 | 2160.11917 | 720.71124 | |
|  |  |  |  |  | TEPPYIEESEIPGHPQNLNPYDLVEHMGK | 4.46 | 3 | 3333.57383 | 1111.86279 | |
|  |  |  |  |  | DADMIMLLVTR | 3.13 | 2 | 1277.65837 | 639.33282 | |
|  |  |  |  |  | DDQCIVVECLVVTESAFTK | 5.29 | 2 | 2213.04668 | 1107.02698 | |
|  |  |  |  |  | VDTVAHESNHLLGSLHDGER | 0.44 | 2 | 2186.08525 | 1093.54712 | |
|  |  |  |  |  | SFLNDAVMQMDVNEEMEKVATMAR | 0.22 | 5 | 2778.1822 | 556.44226 | |
|  |  |  |  |  | SDIVNRYKFSECSK | 0.25 | 2 | 1733.81328 | 867.41028 | |
|  |  |  |  |  | YYCSHATGLAK | 2.93 | 3 | 1270.5875 | 424.20068 | |
|  |  |  |  |  | FTAMGLAYLASVCK | 5.34 | 2 | 1531.76579 | 766.38654 | |
|  |  |  |  |  | IVEVINDGKSFLNDAVMQMDVNEEMEK | 1.03 | 3 | 3099.42771 | 1033.82703 | |
| CliEnzMtp23 | 807.37 | 80 | 23 | 30.1 | IVEVINDGK | 0.68 | 2 | 987.52971 | 494.26849 | |
|  |  |  |  |  | AEDCPDTDGYIMGNRR | 0.22 | 2 | 1886.75578 | 943.88153 | |
|  |  |  |  |  | DGTVKFTAMGLAYLASVCKK | 5.52 | 3 | 2160.11917 | 720.71124 | |
|  |  |  |  |  | LLGIDPFTKETEPAYIEESAIPGHPNYLYADHIVDR | 5.73 | 4 | 4084.03883 | 1021.76483 | |
|  |  |  |  |  | DADLIMLIITRK | 2.66 | 2 | 1401.81279 | 701.41003 | |
|  |  |  |  |  | DDQCIVVECLVVTESAFTK | 5.29 | 2 | 2213.04668 | 1107.02698 | |
|  |  |  |  |  | YYCSHATGLAKDADLIMLIITRK | 3.79 | 4 | 2653.3854 | 664.10181 | |
|  |  |  |  |  | YYCSHATGLAKDADLIMLIITR | 4.88 | 3 | 2525.29282 | 842.43579 | |
|  |  |  |  |  | SFLNDAVMQMDVNEEMEKVATMAR | 0.22 | 5 | 2778.1822 | 556.44226 | |
|  |  |  |  |  | ALTEYVTVMYTGVQNLIDTLEMGIKVR | 0.21 | 3 | 3075.59214 | 1025.8689 | |
|  |  |  |  |  | MGKYYCSHATGLAKDADLIMLIITR | 0.24 | 3 | 2857.47416 | 953.1629 | |
|  |  |  |  |  | YYCSHATGLAK | 2.93 | 3 | 1270.5875 | 424.20068 | |
|  |  |  |  |  | LLGIDPFTK | 2.74 | 2 | 1003.58275 | 502.29382 | |
|  |  |  |  |  | DADLIMLIITR | 4.29 | 2 | 1273.7199 | 637.36359 | |
|  |  |  |  |  | VDTVAHETAHLLGSPHDGEPK | 5.4 | 3 | 2210.08152 | 737.36536 | |
|  |  |  |  |  | FTAMGLAYLASVCK | 5.34 | 2 | 1531.76579 | 766.38654 | |
|  |  |  |  |  | ETEPAYIEESAIPGHPNYLYADHIVDR | 5.03 | 3 | 3099.47011 | 1033.82703 | |
|  |  |  |  |  | NATNKFKFSECSK | 0.23 | 2 | 1561.73625 | 781.37177 | |
|  |  |  |  |  | AEDCPDTDGYIMGNR | 1.83 | 2 | 1713.7022 | 857.35474 | |
|  |  |  |  |  | ALTEYVTVMYTGVQNLIDTLEMGIK | 7.53 | 3 | 2802.42862 | 934.81439 | |
|  |  |  |  |  | DADLIMLIITRKMGELK | 0.21 | 2 | 1976.07549 | 988.54138 | |
|  |  |  |  |  | IVEVINDGKSFLNDAVMQMDVNEEMEK | 1.03 | 3 | 3099.42771 | 1033.82703 | |
|  |  |  |  |  | AEDCPDTDGYIMGNRRNATNK | 0.21 | 2 | 2415.98662 | 1208.49695 | |
| CliEnzMtp24 | 25.21 | 4.79 | 2 | 71.5 | DRYESPTFLQDLENLWK | 3.39 | 3 | 2154.05271 | 718.68909 | |
|  |  |  |  |  | IAFLPFGYILDK | 2.91 | 2 | 1396.7874 | 698.89734 | |
| CliEnzAML01 | 42.4 | 14.15 | 4 | 60 | NVAGFEPVDYWWDNNYHQIAFGR | 4.41 | 3 | 2798.27469 | 933.42975 | |
|  |  |  |  |  | DKITEYLNYLIDIGVAGFR | 3.94 | 2 | 2200.16753 | 1100.5874 | |
|  |  |  |  |  | GHGAGGFGTILTFFESR | 3.79 | 3 | 1753.86637 | 585.29364 | |
|  |  |  |  |  | mAVAFmLAWPYGLPR | 2.92 | 2 | 1754.87627 | 877.94177 | |
| CliEnzTGa01 | 51.57 | 5.99 | 2 | 85.5 | IQVGKPFDLVASFVNPLDR | 4.97 | 3 | 2115.1622 | 705.72559 | |
|  |  |  |  |  | RGEVGFSYDSPFVFSEVNADIIHWQK | 3.56 | 4 | 3027.46645 | 757.62207 | |
| **Protease inhibitors** | | | | | | | | | |  |
| CliPInTIL14 | 175.91 | 5.78 | 8 | 212.9 | SDEcIIENVLDcFQTGK | 5.38 | 2 | 2027.90666 | 1014.45697 | |
|  |  |  |  |  | LKSDEcIIENVLDcFQTGK | 4.97 | 3 | 2269.08487 | 757.03314 | |
|  |  |  |  |  | LYGLcGVLNGDISDDLFSR | 4.55 | 2 | 2114.02007 | 1057.51367 | |
|  |  |  |  |  | IFTSLQDIWIIPVDK | 3.69 | 2 | 1787.99382 | 894.50055 | |
|  |  |  |  |  | ETVLVTVESFLR | 3.48 | 2 | 1392.77422 | 696.89075 | |
|  |  |  |  |  | VDLGEVYFLVGMMYR | 2.72 | 2 | 1791.88005 | 896.44366 | |
|  |  |  |  |  | WDGKETVLVTVESFLR | 2.61 | 3 | 1878.99808 | 627.00421 | |
|  |  |  |  |  | RFEIYVNNEEETIPYIGQEVLIDK | 3.11 | 3 | 2911.46994 | 971.1615 | |
| **Other venom components** | | | | | | | | | |  |
| CliOthCAP02 | 20.45 | 54.08 | 11 | 44.4 | FTPDFIKPFTSDHATGHFTQMAWSTTWR | 0.98 | 3 | 3329.51186 | 1110.5083 | |
|  |  |  |  |  | RDSWTQLYVCNYGPAGNIDDSEMYK | 0.21 | 3 | 3000.2468 | 1000.75378 | |
|  |  |  |  |  | RGSAPLSFELDSEGNQWLPYSMGIPMNQPMQINLKFSVPK | 0.47 | 4 | 4541.10463 | 1136.03162 | |
|  |  |  |  |  | VEGSNKWQTRQIISDVYK | 0.86 | 3 | 2153.0643 | 718.35962 | |
|  |  |  |  |  | GPDFDETDFGNFIFNCDFRPESSSDCNSK | 3.88 | 3 | 3404.37522 | 1135.46326 | |
|  |  |  |  |  | GSAPLSFELDSEGNQWLPYSMGIPMNQPMQINLK | 1.08 | 4 | 3794.73183 | 949.43842 | |
|  |  |  |  |  | KVENFDVGQNIYTSTITAVKPPESFWVDAIR | 4.89 | 4 | 3524.81093 | 881.95819 | |
|  |  |  |  |  | VENFDVGQNIYTSTITAVKPPESFWVDAIR | 1.61 | 3 | 3396.71115 | 1132.90857 | |
|  |  |  |  |  | LPAAGDMLEMEWDDELAQIAQK | 1.31 | 2 | 2474.15801 | 1237.58264 | |
|  |  |  |  |  | DSWTQLYVCNYGPAGNIDDSEMYKVGKPCDK | 0.3 | 3 | 3612.60678 | 1204.87378 | |
|  |  |  |  |  | NNIATGKDQSGR | 0.21 | 2 | 1261.61833 | 631.31281 | |
| CliOthCAP05 | 419.12 | 73.23 | 16 | 42 | KVLYTCNYGPGGNSK | 0.55 | 2 | 1659.78581 | 830.39655 | |
|  |  |  |  |  | SLTSDGPQPSRPSSSDYLLYCDFSNEDPQACK | 5.81 | 3 | 3621.57492 | 1207.86462 | |
|  |  |  |  |  | DGLCAFLIGR | 3.67 | 2 | 1121.57732 | 561.2923 | |
|  |  |  |  |  | LSKNHSYCMSSTCKVIAGGK | 0.54 | 3 | 2244.07706 | 748.6972 | |
|  |  |  |  |  | LATGKETQYQK | 0.57 | 2 | 1267.67119 | 634.33923 | |
|  |  |  |  |  | RLSKNHSYCMSSTCK | 0.21 | 2 | 1874.8206 | 937.91394 | |
|  |  |  |  |  | DGLCAFLIGRFGPNVAGEKAGSFVSFHFAAPGLIFPDGMK | 0.48 | 5 | 4198.0763 | 840.42108 | |
|  |  |  |  |  | YFHPEYNNPFQFQSTYGHFSQVIWAK | 5.73 | 4 | 3235.50547 | 809.63361 | |
|  |  |  |  |  | AGSFVSFHFAAPGLIFPDGMKETK | 3.55 | 3 | 2554.27713 | 852.09723 | |
|  |  |  |  |  | AGSFVSFHFAAPGLIFPDGMK | 6.31 | 3 | 2196.09702 | 732.70355 | |
|  |  |  |  |  | FEHDSGDQRAVGNFSVGQNLFQSSGSLSINWNGVKMWYTSEVK | 0.31 | 5 | 4822.25782 | 965.25739 | |
|  |  |  |  |  | LPSAANMMEMEWDDELAAIAQAHANQCKFEHDSGDQR | 9.94 | 4 | 4218.76821 | 1055.44714 | |
|  |  |  |  |  | LPSAANMMEMEWDDELAAIAQAHANQCK | 0.45 | 3 | 3177.36069 | 1059.79175 | |
|  |  |  |  |  | GSEVYQVGSPCSACPKNTKCSDTYPGLCK | 0.98 | 4 | 3251.36391 | 813.59644 | |
|  |  |  |  |  | VHNELRSKLATGK | 0.51 | 3 | 1453.81255 | 485.27704 | |
|  |  |  |  |  | VYTGKYVTAVFNAGEKMTINFGK | 0.42 | 2 | 2554.29668 | 1277.65198 | |
| CliOthLa106 | 142.04 | 11.72 | 2 | 14.9 | IWMDIDSVNDcFFK | 5.77 | 2 | 1789.79473 | 895.401 | |
|  |  |  |  |  | KIWMDIDSVNDcFFK | 5.1 | 2 | 1917.88567 | 959.44647 | |
| CliOthUnd03 | 362.89 | 93.1 | 3 | 3.4 | YCQYGTCYCK | 2.92 | 2 | 1402.51872 | 701.763 | |
|  |  |  |  |  | KYCYNDDDCKSECMVVK | 3.73 | 3 | 2213.89505 | 738.63739 | |
|  |  |  |  |  | YCYNDDDCKSECMVVK | 0.6 | 3 | 2085.81461 | 695.94183 | |
|  |  |  |  |  | YCQYGTCYCK | 0.26 | 2 | 1403.52519 | 702.26624 | |
| CliOthUnd04 | 51.46 | 32.3 | 3 | 17.9 | FcLIEnGLQTVLDFAK | 3.91 | 2 | 1868.94744 | 934.97736 | |
|  |  |  |  |  | TFSITNPNVYLIIAK | 3.49 | 2 | 1693.95122 | 847.47925 | |
|  |  |  |  |  | NcAQGLQFFPTTDEITEFVcK | 2.12 | 2 | 2505.14409 | 1253.07568 | |
| CliOthUnd05 | 33.78 | 68.42 | 2 | 14 | LAELETLINLAK | 4.51 | 2 | 1327.78227 | 664.39478 | |
|  |  |  |  |  | GRPPIAFGVIDPLK | 3.43 | 3 | 1479.86789 | 493.96082 | |
| CliOthIGI01 | 57.53 | 14.12 | 2 | 29.8 | GSDTAVLFLYPLLPENKD | 4.39 | 2 | 1992.03081 | 996.51904 | |
|  |  |  |  |  | VLEcEAGASPSPMIVYWLK | 4.36 | 2 | 2150.0667 | 1075.53699 | |
| CliOthSFU01 | 134.73 | 9.97 | 2 | 38 | IPGTPNIVcLFSVGNcScENK | 4.19 | 3 | 2366.0936 | 789.36938 | |
|  |  |  |  |  | ELTcGLPFLELK | 4.14 | 2 | 1419.7542 | 710.38074 | |
| CliOthLTR01 | 140.31 | 8.93 | 11 | 278.2 | SYVGEPIQLDNFDLEEVHIK | 4.46 | 3 | 2345.1652 | 782.39325 | |
|  |  |  |  |  | NQEIFGDKIEDIPIDETFIK | 4.35 | 3 | 2364.19645 | 788.737 | |
|  |  |  |  |  | AAGLFDELLHLVR | 4.34 | 3 | 1453.81702 | 485.27719 | |
|  |  |  |  |  | LGSIFALPQDPIYSVNIK | 3.31 | 2 | 1975.08757 | 988.04742 | |
|  |  |  |  |  | ELLYPFIEPTVEMIR | 3.17 | 2 | 1849.97795 | 925.49261 | |
|  |  |  |  |  | GLIPVVSNFGGEELSFVYPDQK | 2.79 | 2 | 2395.21855 | 1198.11292 | |
|  |  |  |  |  | QSTDLIPFIDSVTAcNFIK | 2.66 | 2 | 2169.08891 | 1085.0481 | |
|  |  |  |  |  | nGYESIIFSVIGYGGK | 2.66 | 2 | 1704.8488 | 852.92804 | |
|  |  |  |  |  | LAAYDVLVNLFDDDDLAK | 2.45 | 2 | 2010.00749 | 1005.50739 | |
|  |  |  |  |  | YQYTGPHPFYWQNLFQIPQPWEGTYR | 2.37 | 3 | 3316.56589 | 1106.19348 | |
|  |  |  |  |  | IGESYHHVPTSMGLPLTWGSNAINIISPR | 2.11 | 4 | 3147.60561 | 787.65686 | |
| CliOthSCO01 | 27.76 | 3.86 | 2 | 121.3 | YGVSTEDWLYDEGcDIIEcK | 5.46 | 2 | 2452.03301 | 1226.52014 | |
|  |  |  |  |  | DGTVTDDIETFAFDWIEETDK | 2.83 | 2 | 2447.08159 | 1224.04443 | |
